# Neural Activity Alterations and Their Association With Neurotransmitter and Genetic Profiles in Schizophrenia: Evidence From Clinical Patients and Unaffected Relatives

**DOI:** 10.1111/cns.70218

**Published:** 2025-02-09

**Authors:** Zixuan Guo, Shu Xiao, Shilin Sun, Ting Su, Xinyue Tang, Guanmao Chen, Pan Chen, Ruoyi Chen, Chao Chen, Jiaying Gong, Zibin Yang, Li Huang, Yanbin Jia, Ying Wang

**Affiliations:** ^1^ Medical Imaging Center First Affiliated Hospital of Jinan University Guangzhou China; ^2^ Institute of Molecular and Functional Imaging Jinan University Guangzhou China; ^3^ Department of Medical Imaging The Affiliated Guangdong Second Provincial General Hospital of Jinan University Guangzhou China; ^4^ Department of Radiology The Affiliated Brain Hospital of Guangzhou Medical University Guangzhou China; ^5^ Department of Radiology Six Affiliated Hospital of Sun Yat‐Sen University Guangzhou China; ^6^ Department of Psychiatry First Affiliated Hospital of Jinan University Guangzhou China

**Keywords:** first‐degree relatives, gene expression, neurotransmitter, resting‐state functional MRI, schizophrenia

## Abstract

**Background:**

The pattern of abnormal resting‐state brain function has been documented in schizophrenia (SCZ). However, as of yet, it remains unclear whether this pattern is of genetic predisposition or related to the illness itself.

**Methods:**

A systematical meta‐analysis was performed to identify resting‐state functional differences in probands and their high‐risk first‐degree relatives of schizophrenia (FDRs‐SCZ) using Seed‐based d Mapping software. Subsequently, spatial associations between postmortem gene expression and neurotransmitters distribution data and neural activity alterations were conducted to uncover neural mechanisms underlaying FDRs‐SCZ and SCZ from a multidimensional perspective.

**Results:**

A total of 13 studies comprising 503 FDRs‐SCZ and 605 healthy controls (HCs) and 129 studies comprising 6506 patients with SCZ and 6982 HCs were included. Compared to HCs, FDRs‐SCZ displayed increased spontaneous functional activity in the bilateral anterior cingulate cortex/medial prefrontal cortex (ACC/mPFC); patients with SCZ showed decreased spontaneous functional activity in the bilateral ACC/mPFC, bilateral postcentral gyrus, and right middle temporal gyrus as well as increased spontaneous functional activity in the bilateral striatum. The altered functional activity in FDRs‐SCZ and SCZ shared similar spatial associations with genes enriched in potassium ion transmembrane transport, channel activity, and complex. The FDRs‐SCZ and SCZ‐related brain functional patterns were additionally associated with dopaminergic, serotonergic, and cholinergic neurotransmitter distribution.

**Conclusions:**

SCZ‐related resting‐state functional, neuroimaging transcriptomes, and neurotransmitters abnormalities may exist in high‐risk unaffected FDRs‐SCZ, rather than just in overt SCZ. The study extended the evidence that altered brain function, along with their spatial correlations to genetics and neurotransmitter systems, may associate with genetic vulnerability for SCZ.

## Graphical abstract

Altered resting‐state brain function, along with their spatial correlations to genetics and neurotransmitter systems, may associate with genetic vulnerability for schizophrenia.

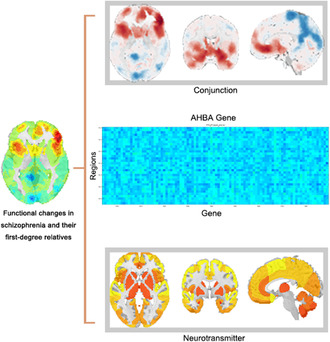

## 1 Introduction

Schizophrenia (SCZ) is a severe psychiatric disorder, and has a substantial genetic risk and effect, with an estimated heritability of 80% [1, 2]. As would be expected in disorders with prominent genetic determinants, probands and their non‐psychotic first‐degree relatives of SCZ (FDRs‐SCZ) experience impaired brain function [3, 4]. Researching FDRs‐SCZ is vital as it allows the investigation of SCZ‐related traits without the confounding effects of antipsychotic medication or the disease's long‐term impact [5]. Despite extensive research over many years, the underlying neuro‐pathophysiology across probands and their unaffected FDRs‐SCZ as well as how brain function abnormalities relate to genetic susceptibility to SCZ remain poorly understood.

Over the past few decades, functional magnetic resonance imaging (fMRI) has shed light on the neurobiological underpinnings of SCZ and its familial risk factors, using the blood‐oxygen‐level‐dependent (BOLD) signal to indirectly measure neural activity [6, 7]. The advent of fMRI has enabled the detection of subtle functional alterations in patients with SCZ and has facilitated the elucidation of the relationship between brain abnormalities and the diverse clinical presentations of patients [8]. Task‐based fMRI meta‐analyses have identified abnormal functional activation in FDRs‐SCZ, especially in areas like the anterior cingulate cortex/medial prefrontal cortex (ACC/mPFC) and the precuneus during cognitive tasks [3, 4]. These findings suggest a genetic predisposition to SCZ that is independent of medication or disease state [9]. Therefore, analyzing the brain function of unaffected FDRs‐SCZ could reveal the roots of the brain dysfunctions observed in patients with SCZ [9]. Furthermore, patients with SCZ show more widespread abnormal brain activation during emotional and cognitive tasks [10, 11, 12]. Advances in neuroimaging have highlighted the significance of resting‐state fMRI, which records the brain's intrinsic activity without task engagement [13, 14, 15]. Resting‐state functional imaging techniques mainly include (fractional) amplitude of low‐frequency fluctuations (fALFF/ALFF), regional homogeneity (ReHo), and cerebral blood flow (CBF) [arterial spin labeling (ASL), positron emission tomography (PET), and single‐photon emission computed tomography (SPECT)] [16, 17]. Together, these modalities offer a comprehensive evaluation of disorder‐related impacts on psychiatric conditions like SCZ [18, 19] and major depressive disorder [20, 21]. Recent meta‐analyses have reported altered spontaneous functional activity in regions such as the striatum, inferior frontal gyrus (IFG), ACC/mPFC, and insula in SCZ [18, 19]. However, previous meta‐analyses had limitations; for example, one study with 16 studies focused only on drug‐free adolescents and adults with SCZ using a liberal uncorrected threshold [19]. Another study with 46 studies had omitted literature using activation likelihood estimation (ALE) software [18]. A complementary meta‐analysis using Seed‐based d Mapping (SDM‐PSI) software could reduce false‐positive findings, [22, 23] provide more reliable results through family‐wise error (FWE) correction for multiple comparisons [24], and allow for a conjunction analysis between two targeted groups [25, 26]. More importantly, these studies failed to answer the question of whether these abnormal resting‐state function patterns are related to the illness itself or to genetic vulnerability to the disorder. Consequently, a comprehensive meta‐analysis that thoroughly characterizes resting‐state brain function across patients with SCZ, unaffected FDRs‐SCZ, and healthy controls (HCs) is urgently needed.

Advances in neuroimaging transcriptomes have enabled spatial correlations between whole brain gene expression, as documented in the Allen Human Brain Atlas (AHBA), and neuroimaging changes across multiple psychiatric disorders [27, 28, 29]. This bridges the gap between microscale disorder‐related gene expression and macroscale functional alterations [27, 30]. Additionally, exploring the spatial relationships between functional imaging modalities and neurotransmitter systems derived from nuclear imaging could offer a cross‐modal assessment of the disorder [31]. A pure correlation between functional modalities and gene expression or neurotransmitters would yield valuable insights into both unaffected FDRs‐SCZ and their affected probands.

This study aims to thoroughly characterize resting‐state static spontaneous brain function patterns (ALFF/fALFF, ReHo, and CBF) across patients with SCZ, unaffected FDRs‐SCZ, and HCs, employing a conservative FWE correction. Initially, we conducted separate voxel‐based functional meta‐analyses for FDRs‐SCZ and SCZ using SDM‐PSI software to identify functional brain alterations [32]. Subgroup analyses were conducted to further investigate functional brain alterations in various stages of SCZ. Finally, we performed spatial correlations between these functional alterations and gene expression/neurotransmitter systems in both FDRs‐SCZ and SCZ. Based on previous evidence, we hypothesized that unaffected high‐risk FDRs‐SCZ and SCZ would exhibit shared brain functional alterations. Additionally, genetic and neurotransmitter disruptions associated with SCZ were also expected to be present in high‐risk FDRs‐SCZ.

## 2 Materials and Methods

### 2.1 Literature Search

Comprehensive and systematic searches of the PubMed, Embase, SinoMed, Web of Science, Chinese National Knowledge Infrastructure, and WanFang databases were performed to identify eligible studies published through November 03, 2023. The current study was conducted with reference to the Preferred Reporting Items for Systematic Reviews and Meta‐Analyses (PRISMA) guidelines [33] and has been registered with PROSPERO database (registration number: CRD42024547650). The medical subject heading (MeSH) keywords has been provided in Supporting Information.

### 2.2 Study Selection Criteria

Studies were included if they met the following criteria: (i) published as original work in an English or Chinese peer‐reviewed journal; (ii) involved unaffected FDRs‐SCZ or patients with a primary diagnosis of SCZ; (iii) compared resting‐state functional activity between FDRs‐SCZ or SCZ and HCs at the whole‐brain level; (iv) reported peak coordinates of results in three‐dimensional standard coordinates (Talairach or Montreal Neurological Institute [MNI]); and (v) provided details that could be obtained through a reasonable request to the corresponding author if not reported in the original manuscripts.

Studies were excluded for the following reasons: (i) FDRs‐SCZ or patients with SCZ had comorbid neurological or other physical diseases; (ii) patients were diagnosed with schizoaffective disorder; (iii) three‐dimensional coordinates in stereotactic space were unobtainable; (iv) data overlapped with another included publication, in which case only the most recent or largest sample study was included; (v) absence of an HCs group; (vi) studies reported only region of interest (ROI) findings rather than whole‐brain results; and (vii) studies reported only follow‐up data without baseline data.

### 2.3 Quality Assessment and Data Extraction

We used a 10‐point checklist previously used by several meta‐analyses [34, 35, 36] to assess the quality and completeness of each study (details in the Table S1). This checklist was divided into 3 categories: participants (items 1–4), methods for image acquisition and analysis (items 5–8), and results and conclusions (items 9 and 10). Two authors (T.S. and Y.L.P.) searched the literatures, evaluated, and selected study independently, and the third investigator (Y.W.) would make the final decision if three has any discrepancies.

For each included study, we extracted the peak coordinates of differences and the information of the first author, year of publication, sample size, gender, mean age, illness duration, drug treatment status, PANSS scores, and imaging methodology parameters (i.e., Imaging technique, statistic software and correction methods).

### 2.4 Data Analysis

#### 2.4.1 Main Meta‐Analysis

We first conducted a meta‐analysis of resting‐state functional activity differences between FDRs‐SCZ or SCZ patients and HCs by using the SDM‐PSI software (version 6.21, https://www.sdmproject.com/). The SDM‐PSI, a voxel‐based meta‐analysis software, can control the results of independent studies and all the information included in the study can be used in the same map [37]. To better understand how voxel‐based meta‐analyses work, it should first be noted that all neuroimaging studies report included in our study show the, from each cluster of significant differences, the coordinates of the ‘voxel’ (i.e., the 3‐dimensional pixel) of brain regions where the difference is maximum between the FDRs‐SCZ or SCZ and controls [37]. First, we created the signed differential map of the potential upper and lower bounds of possible effect sizes for each study based on the reported peak coordinates, the level of statistical significance, and *t*‐values extracted from each original study [38]. Second, different specific masks for functional MRI are used to improve the accuracy of statistical parameter mapping (t‐maps). Third, imputed effect size maps were then consolidated in a standard random‐effects model weighting sample size (i.e., studies with larger sample size or lower variability contribute more) and controlling results for moderators of imaging factors [38, 39]. Finally, images were imputed for each included study, and statistical significance was assessed via a subject‐based permutation test [32]. We carried out as described in the SDM‐PSI tutorial and previous publications [32, 40] and used MRIcron software package (www.mricro.com/mricron/) to visualize SDM‐PSI maps. The analyses were conducted using the threshold‐free cluster enhancement (TFCE) approach with 5000 permutations to correct for multiple comparisons of results of each modality separately, and results were considered significant at TFCE‐corrected *p* < 0.05 with cluster extend ≥ 10 voxels.

For an exploratory analysis, regional gray matter volume (GMV) differences between FDRs‐SCZ and HCs and between SCZ and HCs were also analyzed using SDM‐PSI software with a similar procedure (see Supporting Information).

#### 2.4.2 Conjunction Analysis

We then performed an overlapping of the functional difference based on above meta‐analytic results‐maps to identify brain regions with functional alterations both in FDRs‐SCZ and SCZ using conjunction analysis through the multimodal meta‐analysis in SDM software [41]. The conjunction analysis accounts for the presence of noise in the estimation of the *p*‐values [41, 42].

#### 2.4.3 Subgroup Meta‐Analysis

To explore underlying clinical and methodological heterogeneity, we conducted the meta‐analyses in the following subgroups: different clinical status (i.e., unmedicated first‐episode SCZ studies, first‐episode SCZ studies, unmedicated SCZ studies [included who underwent a medication wash‐out period of longer than 4 weeks], medicated SCZ studies, chronic SCZ studies [those with an illness duration over 2 years] [43]), different functional imaging methodology (i.e., ALFF, fALFF, ReHo and CBF), and analysis procedures (i.e., excluding the studies of insufficient number subjects (*n* < 10) or uncorrected for statistics). Subgroup meta‐analysis was not performed when the number of datasets was insufficient (no less than 5).

### 2.5 Analyses of Heterogeneity, Jackknife Sensitivity, and Publication Bias

The values from peak coordinates were extracted for information to assess the heterogeneity and publication bias. Heterogeneity between studies was examined to assess robustness of findings using the *I* ^2^ statistic, where *I* ^2^ < 50% commonly indicates low heterogeneity [44]. A whole‐brain voxel‐based jackknife sensitivity analysis was performed to evaluate the repeatability of results (i.e., removing one study and repeating the analyses, then putting that study back, and removing another study and repeating the analysis, and so on). If a brain region is still considerable in all or most of the study combinations, the finding is considered highly repeatable. To determine potential publication bias, funnel plots were created for visual inspection and Egger's tests were performed [45]. An asymmetric plot and *p*‐value < 0.05 were suggested a significant bias of publication.

### 2.6 Meta‐Regression Analysis

It should be noted that relevant demographic variables (age, percentage of males) and clinical variables (illness duration and PANSS total scores) may have potential effects on the results. We selected a more conservative threshold of *p* < 0.05, TFCE correction with cluster extend ≥ 10 voxels, and discarding findings in regions other than those detected in the main analyses. Results are reported in MNI space.

### 2.7 Correlations With Gene Expression and Neurotransmitters

Brain gene expression profiles of six human post‐mortem donors from the AHBA database (http://www.brain‐map.org) were acquired [46, 47], encompassing normalized microarray expression data of over 20,000 genes measured at 3702 spatially distinct brain tissue samples [48]. Partial least squares (PLS) regression analyses were employed to examine the correlation between transcriptional patterns and the disparities observed among groups in the primary maps [49] (see Supporting Informations).

Gene enrichment analysis, encompassing both descending and ascending sequences, was conducted to identify enriched Gene Ontology concepts using the Gorilla tool (http://cbl‐gorilla.cs.technion.ac.il/) [50]. The evaluated ontology categories consist of biological process, molecular function, and cellular component. Significance of enrichment analysis was determined using the Benjamini‐Hochberg false discovery rate (FDR)‐corrected *q* < 0.05 threshold [51].

For neurotransmitter analysis, we utilized the JuSpace toolbox (version 1.5, https://github.com/juryxy/JuSpace, default settings, computing option 3) to extract the distribution of neurotransmitters across different brain regions. This allowed us to examine the spatial correlations between abnormal functional changes and neurotransmitter systems based on atlases, which include serotonergic, dopaminergic, gamma‐aminobutyric acid (GABA)‐ergic, and glutamatergic neurotransmission (see Supporting Information) [31].

## 3 Results

### 3.1 Included Studies and Sample Characteristics

**FIGURE 1:**
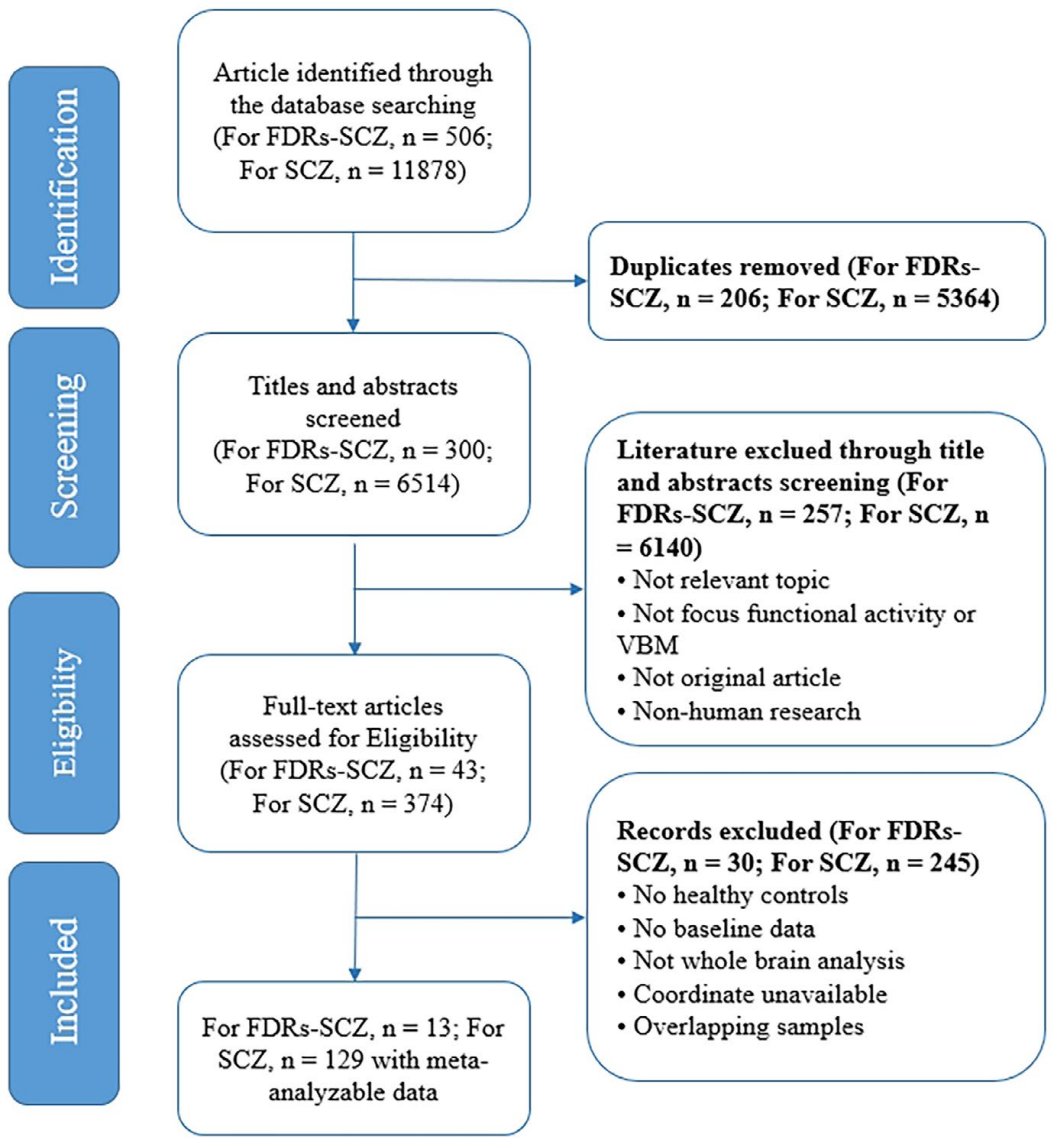
Flow chart of meta‐analysis of resting‐state functional brain activity of patients with schizophrenia and their first‐degree relatives.

A flow diagram of the identification and selection process for included studies is provided in Figure 1. The PRISMA checklist of our meta‐analysis is presented in Table S2. Finally, 15 datasets from 13 studies comprising 503 FDRs‐SCZ and 605 HCs as well as 175 datasets from 129 studies comprising 6506 patients with SCZ and 6982 HCs were identified for functional analysis. There were no significant differences in age and sex distribution between FDRs‐SCZ and HCs. There were no significant differences in age between SCZ and HCs in the included functional studies. However, the sex distribution was significantly different between patients with SCZ and HCs (*χ* ^2^ = 11.24, *p =* 0.001), with more males in patients with SCZ.

The overall demographic and general clinical characteristics of study subjects as well as methodological aspects and quality scores of each included study are summarized in (Tables S3 and S4).

### 3.2 Meta‐Analysis

#### 3.2.1 Main Meta‐Analysis

**FIGURE 2:**
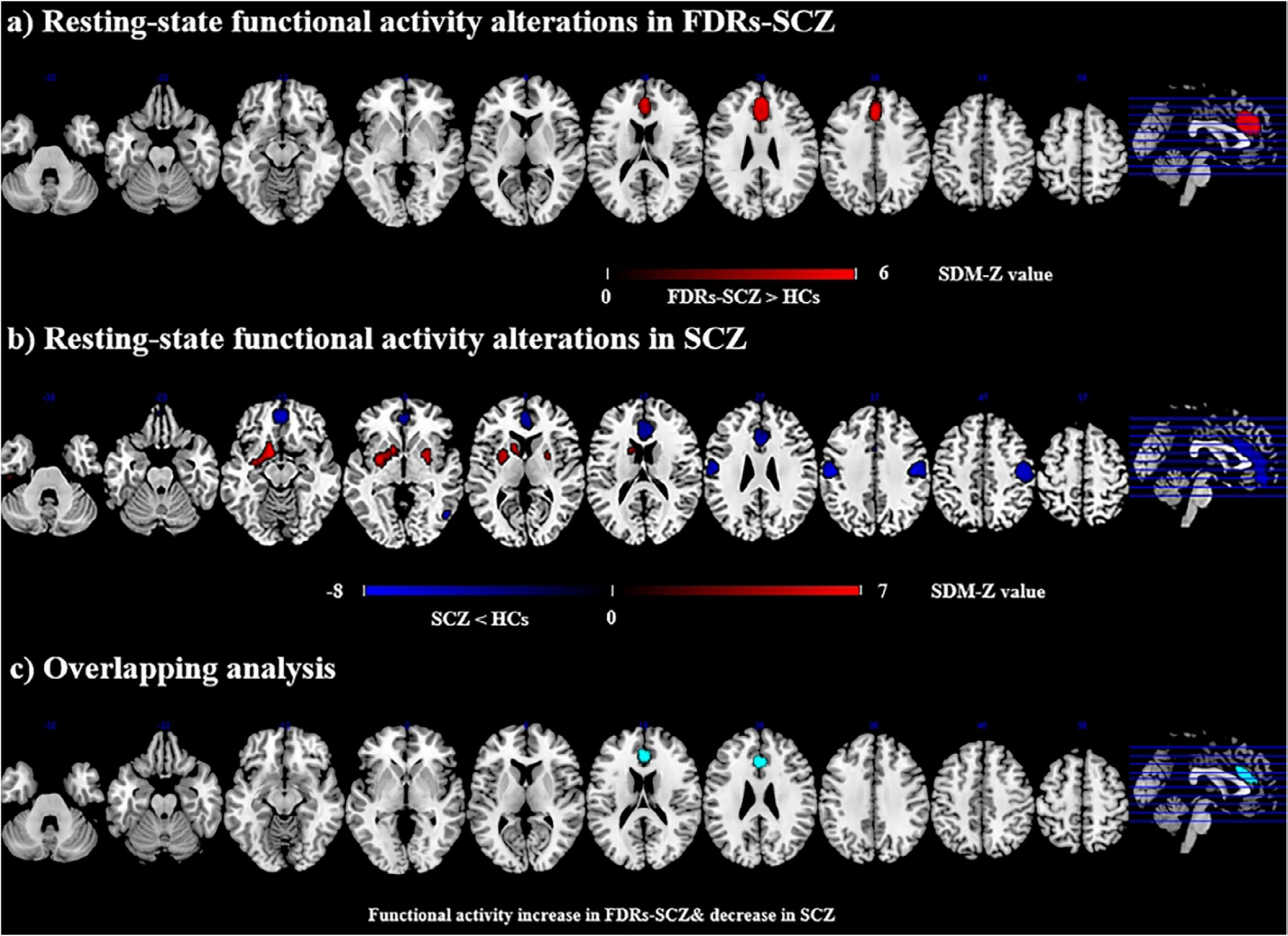
Results of resting‐state functional brain activity differences of FDRs‐SCZ and SCZ. (a) resting‐state functional activity difference between FDRs‐SCZ and HCs. (b) resting‐state functional activity difference between SCZ and HCs. (c) Overlapping of abnormal resting‐state functional brain activity. Areas with decreased resting‐state functional brain activity value are displayed in blue, areas with increased resting‐state functional activity value are displayed in red. The color bar indicates the maximum and minimum SDM‐Z values. FDRs‐SCZ, first‐degree relatives of schizophrenia; SCZ, schizophrenia; HCs, healthy controls; SDM, seed‐based *d* mapping.

Compared to HCs, FDRs‐SCZ displayed increased functional activity in the bilateral ACC/mPFC compared to HCs. No significant decreased functional activity was observed in FDRs‐SCZ (Figure 2a and Table 1). Patients with SCZ showed decreased resting‐state functional activity in the bilateral ACC/mPFC (extending to the bilateral median cingulate cortex [MCC]), bilateral postcentral gyrus, and right middle temporal gyrus (MTG) as well as increased functional activity in the bilateral striatum (Figure 2b and Table 1).

**TABLE 1:** Resting‐state functional brain activity difference.

Local maximum				Cluster	
Peak region	Peak MNI coordinate (*x*, *y*, *z*)	SDM‐*Z* value	*p*	No. of voxels	Breakdown (No. of voxels)
FDRs‐SCZ>HCs					
Left anterior cingulate/paracingulate gyri, BA 24	−2, 28, 24	5.003	< 0.001	960	Left superior frontal gyrus, medial, BA 32 (293) Left anterior cingulate/paracingulate gyri, BA 32 (288) Right anterior cingulate/paracingulate gyri, BA 24 (179) Right superior frontal gyrus, medial orbital, BA 11 (33)
SCZ>HCs					
Left striatum, BA 48	−26, −2, −6	6.559	< 0.001	983	Left striatum, BA 48 (301) Left lenticular nucleus, putamen, BA 48 (185) Left caudate nucleus, BA 25 (36)
Right striatum	24, 2, 2	5.526	0.008	181	Right striatum, BA 48 (95) Right putamen, BA 48 (82)
SCZ<HCs					
Right superior frontal gyrus, medial orbital, BA 11	2, 50, −12	−5.943	< 0.001	1360	Left anterior cingulate/paracingulate gyri, BA 24 (492) Left superior frontal gyrus, medial orbital, BA 11 (283) Right anterior cingulate/paracingulate gyri, BA 24 (264) Right superior frontal gyrus, medial orbital, BA 11 (156) Right median cingulate/paracingulate gyri, BA 24 (87) Left median cingulate/paracingulate gyri, BA 24 (63)
Right postcentral gyrus, BA 3	46, −16, 38	−7.410	< 0.001	742	Right precentral gyrus, BA 6 (358) Right postcentral gyrus, BA 3 (314)
Left postcentral gyrus, BA 3	−58, −18, 36	−7.321	< 0.001	463	Left postcentral gyrus, BA 3 (344) Left precentral gyrus, BA 4 (47)
Right middle temporal gyrus, BA 37	52, −64, 0	−5.751	0.017	77	Right middle temporal gyrus, BA 37 (62)

Abbreviations: FDRs‐SCZ, first‐degree relatives of schizophrenia; HCs, healthy controls; MNI, Montreal Neurological Institute; SCZ, schizophrenia; SDM, the Seed‐based *d* Mapping.

For exploratory analyses, the results of GMV meta‐analyses are presented in (Figures S1 and S2 and Tables S5–S8).

#### 3.2.2 Conjunction Analysis

After overlapping the functional differences in FDRs‐SCZ and patients with SCZ based on the above meta‐analyses, FDRs‐SCZ showed increased functional activity and patients with SCZ showed decreased functional activity in the bilateral ACC/mPFC (Figure 2c and Table 2).

**TABLE 2:** Overlapping analysis of functional difference.

Local maximum		Cluster	
Region	Peak MNI coordinate (*x*, *y*, *z*)	No. of voxels	Breakdown (No. of voxels)
Functional activity increase in FDRs‐SCZ & decrease in SCZ			
Left anterior cingulate/paracingulate gyri	−2, 34, 30	314	Left anterior cingulate/paracingulate gyri (195) Right anterior cingulate/paracingulate gyri (91) Left superior frontal gyrus, medial orbital (4) Right superior frontal gyrus, medial orbital (2)

Abbreviations: FDRs‐SCZ, first‐degree relatives of schizophrenia; HCs, healthy controls; MNI, Montreal Neurological Institute; SCZ, schizophrenia.

#### 3.2.3 Subgroup Meta‐Analysis

In the subgroup analyses of different clinical status, no significant difference was found in the unmedicated first‐episode SCZ subgroup (51 datasets), first‐episode SCZ subgroup (65 datasets), and unmedicated SCZ subgroup (52 datasets). The medicated patients with SCZ (101 datasets) showed decreased resting‐state functional activity in the bilateral ACC/mPFC, right postcentral gyrus, and increased resting‐state functional activity in the left striatum (Figure S3 and Table S9). The chronic patients with SCZ (72 datasets) showed decreased resting‐state functional activity in the right postcentral gyrus, bilateral ACC/mPFC, and right MTG and increased resting‐state functional activity in the left striatum and right IFG (Figure S4 and Table S10).

To avoid missing an exploratory interesting effect, we used a conservative threshold of uncorrected *p* < 0.005 to explore the potential effects of different functional imaging methodology. FDRs‐SCZ showed increased ALFF in the bilateral ACC/mPFC (7 datasets) (Figure S5 and Table S11). Patients with SCZ showed decreased ALFF values in the bilateral postcentral gyrus and increased ALFF values in the left striatum, right IFG, and inferior temporal gyrus (ITG) (51 datasets) (Figure S6a and Table S12); patients with SCZ showed decreased fALFF values in the bilateral ACC/mPFC (extending to the bilateral MCC), calcarine fissure, and increased fALFF values in the left striatum and angular gyrus (29 datasets) (Figure S6b and Table S12); patients with SCZ showed decreased ReHo values in the bilateral postcentral gyrus, ACC/mPFC, right ITG (extending to the right MTG), and precuneus and increased ReHo values in the left striatum and superior frontal gyrus (SFG) (50 datasets) (Figure S6c and Table S12); patients with SCZ showed decreased CBF in the bilateral ACC/mPFC (extending to the bilateral MCC), left SFG (extending to the left middle frontal gyrus [MFG]), and right temporal pole and increased CBF in the bilateral striatum (45 datasets) (Figure S6d and Table S12). Subgroup analyses of ReHo (5 datasets) and fALFF (3 datasets) in FDRs‐SCZ were not conducted due to insufficient datasets.

Moreover, we conducted a subgroup analysis of excluding the studies of an insufficient number of subjects (*n* < 10) or uncorrected for statistics. The results of FDRs‐SCZ (15 datasets) were the same as the main results of FDRs‐SCZ. Patients with SCZ (139 datasets) showed decreased resting‐state functional activity in the bilateral postcentral gyrus and increased resting‐state functional activity in the bilateral striatum (Figure S7 and Table S13).

### 3.3 Analyses of Heterogeneity, Jackknife Sensitivity, and Publication Bias

The heterogeneity analysis found that none of the brain regions with altered function showed significant heterogeneity (*I* ^2^ < 50%) (Table S14). The jackknife sensitivity analyses of the functional meta‐analysis showed high replicability and reliability (Table S14). Funnel plot analysis suggested that the findings were not driven by small or noisy studies, and the potential publication bias was not observed after formal statistic tests (*p* > 0.05) (Table S14).

### 3.4 Meta‐Regression Analysis

In addition, we detected no effect of age and sex distribution on resting‐state functional activities in FDRs‐SCZ. No effect of age, sex distribution, illness duration, and PANSS scores were detected on resting‐state functional activities in SCZ.

### 3.5 Correlations With Gene Expression and Enrichment

**FIGURE 3:**
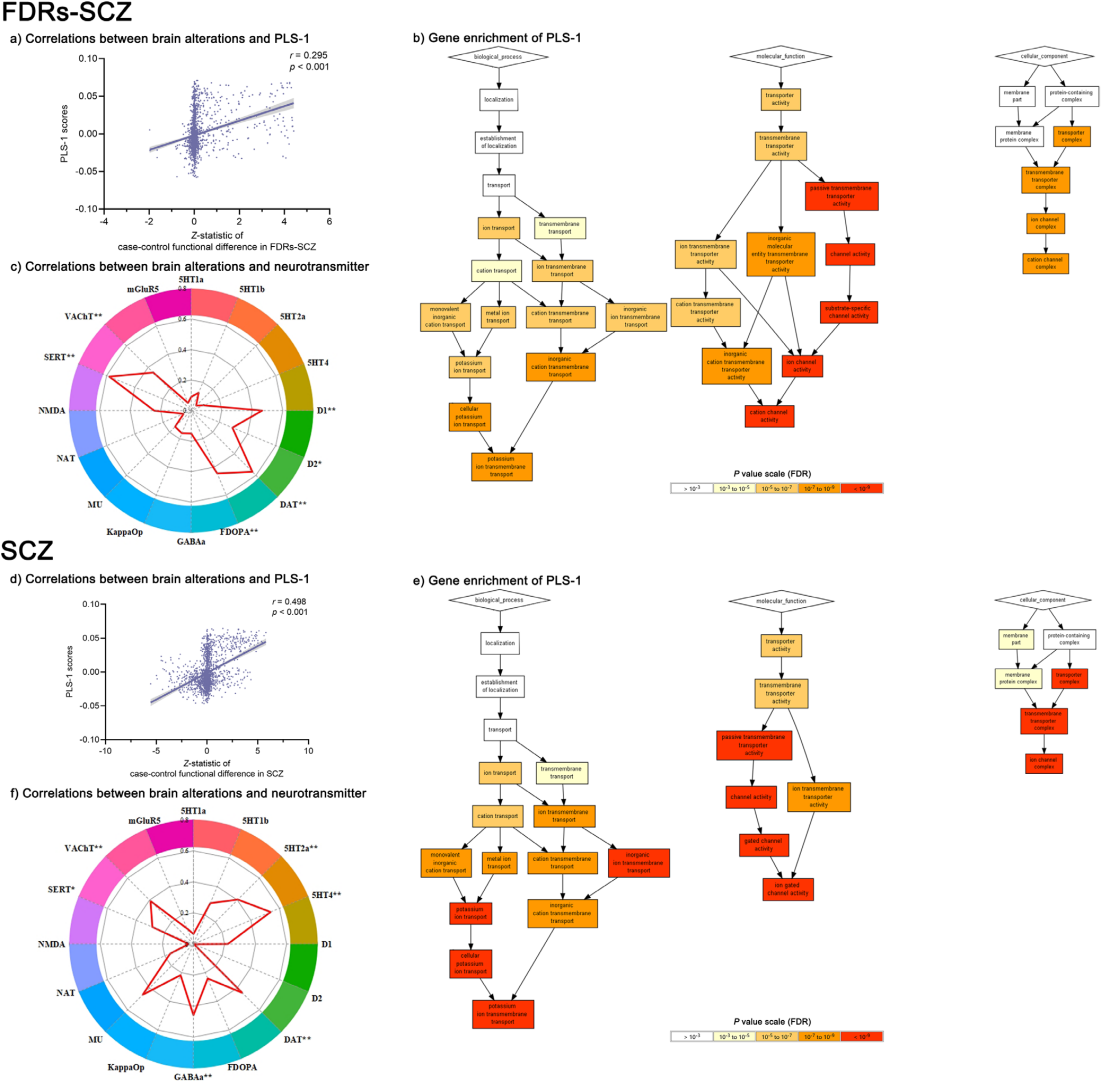
Spatial correlations between functional alterations and gene transcriptional profiles or neurotransmitter distribution in FDRs‐SCZ and SCZ. (a) The PLS‐1 identified a profile of genes that were positively correlated with functional difference in FDRs‐SCZ. (b) Enrichment analysis related to FDRs‐SCZ functional changes. (c) Spatial correlations between functional alterations and neurotransmitter distribution maps in FDRs‐SCZ. (d) The PLS‐1 identified a profile of genes that were positively correlated with functional difference in SCZ. (e) Enrichment analysis related to SCZ functional change. (f) Spatial correlations between functional alterations and neurotransmitter distribution maps in SCZ. *p < 0.05; **p < 0.01. FDRs‐SCZ, first‐degree relatives of schizophrenia; SCZ, schizophrenia; PLS‐1, first component of partial least squares component.

For FDRs‐SCZ, the first component of PLS (PLS‐1) explained 15.36% of the variance in deviation of function (*r* = 0.295, *p* < 0.001, corrected for a 5000 times permutation test for spatial autocorrelation, Figure 3a). In biological process, the enriched genes of functional alterations in FDRs‐SCZ were associated with potassium ion transmembrane transport; in molecular function, the enriched genes were associated with cation channel activity; in the cellular component, the enriched genes were associated with the cation channel complex (Figure 3b, Table S15).

For SCZ, PLS‐1 explained 29.78% of the variance in deviation of function (*r* = 0.498, *p* < 0.001, corrected for a 5000 times permutation test for spatial autocorrelation, Figure 3d). In biological process, the enriched genes of functional alterations in SCZ were associated with potassium ion transmembrane transport; in molecular function, the enriched genes were associated with ion gated channel activity; in the cellular component, the enriched genes were associated with the ion channel complex (Figure 3e; Table S15).

### 3.6 Correlations With Neurotransmitters

On the basis of the above findings in the main meta‐analyses, correlations with the neurotransmitter systems were conducted. The pattern of functional differences in FDRs‐SCZ significantly was associated with dopamine D1 (*r* = 0.464, FDR‐*p* = 0.002), dopamine D2 (*r* = 0.292, FDR‐*p* = 0.033), dopamine transporter (DAT) (*r* = 0.567, FDR‐*p* = 0.002), 6‐fluoro‐(18F)‐L‐3,4‐dihydroxyphenylalanine (FDOPA) (*r* = 0.446, FDR‐*p* = 0.002), serotonin transporter (SERT) (*r* = 0.581, FDR‐*p* = 0.002), vesicular acetylcholine transporter (VAChT) (*r* = 0.353, FDR‐*p* = 0.002) (Figure 3c).

The pattern of functional differences in SCZ was significantly associated with serotonin 5‐hydroxytryptamine receptor subtype 2a (5HT2a) (*r* = −0.405, FDR‐*p* = 0.002), serotonin 5‐hydroxytryptamine receptor subtype 4 (5HT4) (*r* = 0.538, FDR‐*p* = 0.002), DAT (*r* = 0.444, FDR‐*p* = 0.002), GABA (*r* = −0.458, FDR‐*p* = 0.002), SERT (*r* = 0.286, FDR‐*p* = 0.024), and VAChT (*r* = 0.391, FDR‐*p* = 0.002) (Figure 3f).

## 4 Discussion

To our best knowledge, this is the first resting‐state functional meta‐analysis across patients with SCZ, unaffected FDRs‐SCZ, and HCs that integrates neuroimaging, transcriptomes, and neurotransmitter perspectives. The key findings are as follows: (i) FDRs‐SCZ showed increased spontaneous functional activity in the bilateral ACC/mPFC; (ii) patients with SCZ exhibited decreased spontaneous functional activity in the bilateral ACC/mPFC (extending to the bilateral MCC), postcentral gyrus, and right MTG as well as increased functional activity in the bilateral striatum; (iii) gene enrichment analyses indicated shared biological functions in FDRs‐SCZ and SCZ; and (iv) the functional alterations observed in both FDRs‐SCZ and SCZ were spatially linked to the dopaminergic, serotonergic, and cholinergic neurotransmitter systems. The altered resting‐state brain function patterns in FDRs‐SCZ, along with their spatial correlations to genetics and neurotransmitter systems, might relate to genetic vulnerability for SCZ. This study enhances our understanding of the neuro‐pathophysiological mechanisms underlying SCZ spectrum disorders.

In the current study, we identified a convergence of increased functional activity in FDRs‐SCZ and decreased functional activity in SCZ in the bilateral ACC/mPFC. Subgroup analyses showed that patients with chronic SCZ also displayed reduced functional activity in the bilateral ACC/mPFC, whereas unmedicated first‐episode patients with SCZ exhibited no significant alterations compared to HCs. Our findings extended a large number of literature to replicate earlier evidence from ALE methodologies that indicated a reduction in spontaneous functional activity in the ACC/mPFC in SCZ [18]. A previous task‐based meta‐analysis identified increased activation in the ACC/mPFC in FDRs‐SCZ compared to HCs during cognitive processing task [4]. Moreover, our previous voxel‐based morphometry meta‐analysis found decreased gray matter volume in the bilateral ACC/mPFC in SCZ [52]. The ACC/mPFC, crucial components of the limbic system, is essential for integrating cognitive and emotional processes and regulating motivational behaviors [53, 54], which are impaired in SCZ [55]. Postmortem studies have demonstrated decreased mitochondrial, neuronal, and synaptic integrity and density in SCZ, leading to diminished cortical synaptic efficiency [56, 57, 58]. Remarkably, changes in the ACC/mPFC in high‐risk individuals like FDRs‐SCZ precede psychosis onset [57], suggesting a compensatory mechanism that in the opposite direction to the effect of genetic risk, thus nullifying the expected functional activity decrease in the ACC/mPFC [59]. As SCZ progresses from FDRs‐SCZ to first episode and eventually to chronic SCZ, there may be a progressive shift in spontaneous functional activity in the ACC/mPFC, starting with a compensatory increase that progresses to an incompensatable decline. It is widely accepted that changes in brain function typically precede with structural changes. Our exploratory analyses revealed no significant GMV differences between FDRs‐SCZ and HCs, while both functional and structural changes were observed in SCZ in the ACC/mPFC (as detailed in Supporting Information). However, unlike our subgroup findings, previous task‐free meta‐analyses reported scattered and extensive functional activity alterations in unmedicated first‐episode SCZ compared to HCs, impacting areas such as the striatum, thalamus, superior temporal gyrus, IFG, angular gyrus, and insula [18, 19]. This discrepancy may be due to the less conservative thresholds employed in earlier studies, increasing the likelihood of false‐positive results [23]. Overall, our findings highlight the ACC/mPFC as a crucial region associated with genetic vulnerability for SCZ, with functional alterations occurring even before the clinical onset of SCZ.

The present study identified abnormal spontaneous functional activity in the sensorimotor regions of patients with SCZ. Specifically, decreased functional activity was observed in the bilateral postcentral gyrus and right MTG. This finding aligns with previous resting‐state meta‐analyses that reported a reduction in spontaneous functional activity in the postcentral gyrus and MTG in SCZ [34, 60]. The postcentral gyrus is essential for sensory integration, orientation, form perception, and motor control, including action planning and execution [61, 62]. Patients with SCZ often experience psychomotor poverty, impacting fine and gross motor skills, motor speed, facial expressions, and speech production [63]. A previous meta‐analysis noted increased brain activity in the postcentral gyrus and improved cognitive function following cognitive remediation therapy in patients with SCZ [64]. Additionally, auditory hallucinations, a hallmark symptom of SCZ [65], is associated with the MTG, which play a role in auditory processing [66, 67]. Disruptions in the MTG have been linked to the severity of auditory hallucinations in patients with SCZ [66, 67]. These findings suggest that functional alterations in sensorimotor regions may have significant implications in SCZ.

Furthermore, this study also found increased spontaneous functional activity in the bilateral striatum of patients with SCZ. The striatum is integral to motor control, emotion processing, and executive functions [68, 69]. The frontal‐striatal feedback loops are crucial for mediating cognitive and emotional functioning, and the striatum has been implicated in the neural pathology of SCZ [52, 70]. Previous task‐free meta‐analyses have reported reduced spontaneous functional activity in the striatum in patients with SCZ [18, 60]. Additionally, a task‐based meta‐analysis found abnormal activation in patients with SCZ in the striatum during reward‐related tasks, which correlated with the negative symptoms of SCZ [11]. Alongside our findings concerning the ACC/mPFC, this study provides meta‐analytic evidence that deficits in frontal‐striatal circuits may be pivotal in the pathophysiology of SCZ.

The current gene enrichment analysis revealed that the neural substrates of FDRs‐SCZ and SCZ exhibited shared spatial associations with potassium ion transmembrane transport and ion channel activity and complex. Similar to our findings, a previous meta‐analysis also reported that resting‐state brain functional alterations in patients with drug‐naive first‐episode psychosis were spatially associated with ion channel and ion transport [71]. Potassium channels are crucial for modulating neuronal electrical excitability and regulating the duration of action potentials [72]. This modulation is partly influenced by the dopaminergic neurotransmitter, which is a primary target of antipsychotic medications used to treat SCZ. Dysfunctions in potassium channels are considered significant contributors to SCZ development, as they can lead to substantial disturbances in brain function [73, 74, 75]. Therefore, genes associated with cation channel activity that are highly expressed in the prefrontal cortex are considered candidate genes for SCZ [76, 77, 78, 79]. Overexpression of KCNN3, a candidate gene linked to SCZ that encodes the small‐conductance calcium‐activated K (+) channel 3 (SK3) [76, 77], in the forebrain is thought to cause core cognitive deficits associated with SCZ [80, 81]. Animal experiments suggest that targeting the SK3 channel could be a potential therapeutic strategy for improving cognitive functions in patients with SCZ [80, 82]. Additionally, similar gene expression and enrichment abnormalities were observed in unaffected FDRs‐SCZ, supporting the prevailing view that FDRs‐SCZ may carry susceptibility genes similar to those found in patients with SCZ [83].

In terms of aberrant neurotransmitter systems, we found that functional differences in FDRs‐SCZ were spatially associated with the availability of D1, D2, DAT, FDOPA, SERT, and VAChT, and functional differences in SCZ were closely associated with the availability of 5HT2a, 5HT4, DAT, GABA, SERT, and VAChT. The current study indicated that FDRs‐SCZ primarily displayed alterations in dopaminergic neurotransmission, while SCZ involved more extensive impairments across multiple neurotransmitter systems. Supporting our findings, a previous neuroimaging study found that the combination of structural and functional abnormalities was associated with disruptions in the dopaminergic and serotonergic systems in SCZ [18]. Previous vivo imaging and postmortem studies have also reported altered GABA, 5‐HT2a, D1, and D2 receptors binding in the ACC and PFC in schizophrenia spectrum disorders [55, 84, 85, 86]. Synaptic dysfunction is thought to underlie SCZ [87], long‐term changes in brain function can induce synaptic plasticity, the most important of which is the long‐term enhancement and/or inhibition of neurotransmitter release [88]. In turn, altered neurotransmitter release can dynamically modulate information flow in neural circuits [88]. The classic dopamine hypothesis states that overactive dopamine activity leads to the positive symptoms of SCZ [89]. Potassium channels in neurons are crucial for regulating cell firing and communication with adjacent neurons [72], a process partially modulated by the dopaminergic system, which is the primary target of antipsychotic treatments for SCZ. A previous study found that increased presynaptic dopamine synthesis in drug‐naive patients with SCZ was also evident in FDRs‐SCZ [90], suggesting that changes in the dopaminergic system may link to vulnerability to SCZ rather than being exclusive to manifest SCZ [90]. In summary, the interplay of disrupted neurotransmitter systems and aberrant brain function likely contributes to the pathology observed in both SCZ and FDRs‐SCZ.

### 4.1 Limitations

There are some limitations in the present study. First, methodological differences among the included studies, including variations in populations, scanning techniques, parameters, and analysis methods, might act as potential confounders and introduce bias. Second, the present study did not assess the prognosis of the disorder nor did it examine the trajectory and progression of individuals with a genetic high risk for psychosis using MR imaging features [91, 92]. Additionally, the transcriptomic data derived from the AHBA dataset contains tissue samples only from six healthy adult donors. Finally, due to limited data on specific medication therapies, we could not investigate their effects, especially in the chronic SCZ group, where antipsychotic medications may have influenced the observed group differences.

## 5 Conclusions

In conclusion, our meta‐analysis demonstrated that FDRs‐SCZ exhibited increased resting‐state functional activity in the ACC/mPFC, while SCZ exhibited more extensive aberrant resting‐state functional activity in the ACC/mPFC, striatum, and sensorimotor region. Additionally, robust exploratory analyses indicated that neuroimaging transcriptomes and neurotransmitters abnormalities related to SCZ may be present in high‐risk, unaffected FDRs‐SCZ, not just in manifest SCZ. The current study provided multidimensional insights into the neuropathological mechanisms underlying SCZ spectrum disorders.

## Author Contributions

Z.G. and S.X. contributed equally as first authors and took the whole responsibility of literature search, data extraction, data analysis, and manuscript drafting. H.Y. contributed in study design, data interpretation, and major revision of the manuscript. S.S., T.S., X.T., G.C., P.C., R.C., C.C., J.G., and Z.Y. contributed in data collection, data interpretation. and major revision of the manuscript. L.H. contributed in data interpretation. Y.J. and Y.W. contributed as corresponding authors and took the responsibility of collection of all information from the other co‐authors, major revision of the manuscript, and full access to the data, study design, and data interpretation. All authors have read and approved the final version of the submitted manuscript.

## Conflicts of Interest

The authors declare no conflicts of interest.

## Supporting information


Data S1.

## Data Availability

All data generated or analyzed in the present study are included in the published article.
