# Supplementary material for: Neural Activity Alterations and Their Association With Neurotransmitter and Genetic Profiles in Schizophrenia: Evidence From Clinical Patients and Unaffected Relatives
Source: CNS Neurosci Ther. 2025 Feb 9;31(2):e70218. doi: 10.1111/cns.70218 (PMC11807726; doi:10.1111/cns.70218)
Supplement: Supplementary file 1 — Data S1. [file CNS-31-e70218-s001.docx]

**CONTENTS**

**Supplementary methods and results**

**Table S1. Quality assessment checklist (score 0/0.5/1 per item; total score out of 10)**

**Table S2. PRISMA 2020 checklist of our meta-analysis**

**Table S3. Demographic, clinical and imaging characteristics of the included studies of resting-state functional activity for FDRs-SCZ**

**Table S4. Demographic, clinical and imaging characteristics of the included studies of resting-state functional activity for SCZ**

**Table S5. Demographic, clinical and imaging characteristics of the included studies of GMV for FDRs-SCZ**

**Table S6. Demographic, clinical and imaging characteristics of the included studies of GMV for SCZ**

**Table S7. GMV differences between SCZ and HCs**

**Table S8. Overlapping of resting-state functional activity differences and GMV differences in SCZ**

**Table S9. Resting-state functional brain activity differences between medical patients with SCZ and HCs**

**Table S10. Resting-state functional brain activity differences between chronic patients with SCZ and HCs**

**Table S11. Resting-state functional brain activity differences between FDRs-SCZ and HCs in different imaging methodology**

**Table S12. Resting-state functional brain activity differences between SCZ and HCs in different imaging methodology**

**Table S13. Resting-state functional activity alterations in SCZ subgroup (excluded the studies of insufficient number of subjects [n < 10] or uncorrected for statistics)**

**Table S14. Results of heterogeneity, funnel plots and Egger’s test for resting-state functional brain activity and GMV meta-analysis**

**Table S15. Functional enrichment results of the genes related to brain changes in FDRs-SCZ and SCZ**

**Figure S1. GMV differences between SCZ and HCs.**

**Figure S2. Overlapping of abnormal resting-state functional brain activity and GMV in SCZ.**

**Figure S3. Resting-state functional brain activity alterations in medicated patients with SCZ and chronic patients with SCZ.**

**Figure S4. Subgroup of different resting-state functional imaging methodology in FDRs-SCZ.**

**Figure S5. Results of resting-state functional brain activity in SCZ subgroup of different imaging methodology.**

**Figure S6. Results of resting-state functional brain activity and GMV alterations in SCZ subgroup (excluded the studies of insufficient number of subjects [n < 10] or uncorrected for statistics).**

**Supplementary methods and results**

**Supplementary methods**

**Keyword for first-degree relatives of SCZ (FDRs-SCZ)**

(“relative” OR “genetic risk” OR “first-degree relatives” OR “twins” OR “offspring” OR “parents” OR “genetic risk” OR “high risk” OR “liability” OR “family”) AND (“neuroimaging” OR “fMRI” OR “ALFF” OR “amplitude of low-frequency fluctuation” OR “fALFF” OR “fractional amplitude of low-frequency fluctuation” OR “ReHo” OR “regional homogeneity” OR “CBF” OR “cerebral blood flow” OR “PET” OR “positron emission tomography” OR “SPECT” OR “single photon emission computed tomography” OR “ASL” OR “arterial spin labeling”).

**Keyword for SCZ**

(“schizophrenia” OR “schizophrenic disorder” OR “disorder, schizophrenic”) AND (“neuroimaging” OR “fMRI” OR “ALFF” OR “amplitude of low-frequency fluctuation” OR “fALFF” OR “fractional amplitude of low-frequency fluctuation” OR “ReHo” OR “regional homogeneity” OR “CBF” OR “cerebral blood flow” OR “PET” OR “positron emission tomography” OR “SPECT” OR “single photon emission computed tomography” OR “ASL” OR “arterial spin labeling”).

**Literature search of gray matter volume**

Comprehensive and systematic searches were performed to identify studies in the PubMed, Embase, Web of Science, SinoMed, Chinese National Knowledge Infrastructure, and WanFang databases through November 03, 2023, using the keywords related to SCZ (“schizophrenia” OR “schizophrenic disorder” OR “disorder, schizophrenic” OR “relative” OR “genetic risk” OR “first-degree relatives” OR “twins” OR “offspring” OR “parents” OR “genetic risk” OR “high risk” OR “liability” OR “family”) AND (“VBM” OR “morphometry” OR “voxel-based” OR “voxel-wise” OR “voxel-based morphometry” OR “grey matter volume”) and (“neuroimaging” OR “MRI”).

Similar procedures to resting-state functional brain activity analysis, we conducted gray matter volume (GMV) meta-analysis using SDM-PSI software. We performed pooled meta-analyses of all the included studies of GMV.

**Brain gene expression data processing**

**Correlations with gene expression**

For brain gene expression data processing, (1) we updated the probe-to-gene annotations using the latest available National Center for Biotechnology Information (NCBI) data ^1^. (2) A minimum of 50% of samples across all donors were excluded from the analysis using intensity-based filtering. (3) In order to select probes based on the RNA-seq data, we used multiple probes to measure expression levels of a single gene. Moreover, due to the large differences in gene expression between cortical and subcortical regions, including samples from subcortical regions may introduce biases ^2^. (4) To account for potential between-sample differences and donor-specific effects in gene expression, we performed both within-sample cross-gene and within-gene cross-sample normalization by using the scaled robust sigmoid normalization method. We conducted functional meta-analyses within a gray matter mask provided by the SDM-PSI. Consequently, we limited our functional analyses to the samples within this mask, yielding a final sample×gene matrix of 1714×10028. Then, we defined a 3 mm radius sphere centered at the MNI coordinate of this sample and extracted the average *z* value of voxels within the sphere from first-degree relatives of schizophrenia (FDRs-SCZ) or schizophrenia (SCZ) meta-analysis *z* map separately ^3,4^.

Partial least squares (PLS) regression analyses were employed to examine the correlation between transcriptional patterns and the disparities observed among groups in the primary maps ^5^. The PLS components, which are obtained through a linear combination of weighted gene expression values, are ordered according to the explained variances between independent and dependent variables. Prior to the PLS regression, we aligned the gene expression data and between-group difference Z-maps to 1714 voxels due to the sparse distribution of the gene expression data. The first component of PLS (PLS-1) offers an optimal low-dimensional depiction of the covariance within the high-dimensional data matrices ^6^. PLS1 is a linear combination of the predictor variables (i.e., gene expression) that can explain most of the variance in the response variables (i.e., between-group difference Z-maps of fMRI) ^7^. Here, in our PLS 1 model, the gene expression data of the brain voxels (1714 voxels × 10,028 genes) were set as the predictor variables X, and the Z values of the functional maps (1714 voxels × 1 statistics) were set as the response variables Y. Subsequently, the genes were arranged based on their adjusted weight, which signifies their respective contributions to the PLS regression component. We verified our correlation results between brain and transcriptional maps using BrainSMASH toolkit ^8^. There are 5,000 surrogate maps that can be generated to preserve spatial autocorrelations of functional and structural changes in the brain. Finally, we calculated the null distribution of correlations between gene expression and surrogate brain maps, then compared them with our empirical results.

**Correlations with neurotransmitter**

Specifically, we used the main results (*p*-value < 0.05, FWE-corrected) maps derived from SDM-PSI software as input for spatial correlation with the following neurotransmitter as previously suggested ^9,10^: (1) serotonin 5-hydroxytryptamine receptor subtype 1a (5HT1a, map: 5HT1a_WAY_HC36), (2) serotonin 5-hydroxytryptamine receptor subtype 1b (5HT1b, map: 5HT1b_P943_HC22), (3) serotonin 5-hydroxytryptamine receptor subtype 2a (5HT2a, map: 5HT2a_ALT_HC19), (4) serotonin 5-hydroxytryptamine receptor subtype 4 (5HT4, map: 5HT4_sb20), (5) dopamine D1 (D1, map: D1_SCH23390_c11), (6) dopamine D2 (D2, map: D2_RACLOPRIDE_c11), (7) dopamine transporter (DAT, map: DAT_DAT-SPECT), (8) 6-fluoro-(18F)-L-3,4-dihydroxyphenylalanine (FDOPA, map: FDOPA_f18), (9) gamma-aminobutyric acid type a (GABAa, map: GABAa_FLUMAZENIL_c11), (10) (KappaOp, map: KappaOp _LY2795050_hc10_ShokriKojori), (11) μ-opioid receptor (MU, map: MU_CARFENTA-NIL_c11), (12) noradrenaline transporter (NAT, map: NAT_MRB_c11), (13) N-methyl-d-aspartate (NMDA, map: NMDA_ge179_29hc_galovic2021), (14) serotonin transporter (SERT, map: SERT_DASB_HC30), (15) vesicular acetylcholine transporter (VAChT, map: VAChT_feobv_hc18), (16) metabotropic glutamate receptor 5 (mGluR5, map: mGluR5_abp_hc73). Exact permutation-based p-values (with 10,000 permutations) were computed for all analyses, and Bonferroni correction (*p*-value < 0.05/13) was carried out to take into account the overall number of associations tested ^10^.

**Supplementary results**

A total of 19 studies with 19 experiments comprising 1156 FDRs-SCZ and 1067 healthy controls (HCs) were included in GMV meta-analysis. There were no significant differences in age and sex distribution between FDRs-SCZ and HCs. Unfortunately, no significant GMV alteration was observed in FDRs-SCZ compared to HCs (*p*<0.05, TFCE correction with cluster extend is 10 voxels).

A total of 124 studies with 136 experiments comprising 5041 patients with SCZ and 5521 HCs were included in GMV meta-analysis. There were no significant differences in age and sex distribution between patients with SCZ and HCs. Patients with SCZ showed decreased GMV in the bilateral IFG (inferior frontal gyrus) (extending to the bilateral insula, amygdala, putamen, superior temporal gyrus [STG] and middle temporal gyrus [MTG]), anterior cingulate cortex/medial prefrontal cortex (ACC/mPFC), and median cingulate cortex (MCC). No significantly increased GMV was observed in patients with SCZ (Figure S1, Table S7).

In addition, overlapping analysis indicated that patients with SCZ showed increased resting-state functional activity and decreased GMV in the left putamen, as well as decreased resting-state functional activity and decreased GMV in the bilateral ACC/mPFC (extending to the bilateral MCC) (Figure S2 and Table S8).

**Table S1. Quality assessment checklist (score 0/0.5/1 per item; total score out of 10)***

| Category 1: Participants |
| --- |
| 1. Patients were evaluated prospectively, specific diagnostic criteria were applied, and demographic data were reported. |
| 2. Healthy comparison participants were evaluated prospectively, psychiatric and medical illnesses were excluded. |
| 3. Important variables (e.g., age, sex, illness duration, onset, medication status, comorbidity, severity of illness) were checked either by stratification or statistically. |
| 4. Sample size per group > 10. |
| Category 2: Methods for image acquisition and analysis |
| 5. Whole brain analysis was automated with no a priori regional selection. |
| 6. Coordinates reported in a standard space. |
| 7. The imaging technique used was clearly described so that it could be reproduced. |
| 8. Measurements were clearly described so that they could be reproduced. |
| Category 3: Results and conclusions |
| 9. Statistical parameters for significant and important nonsignificant differences were provided. |
| 10. Conclusions were consistent with the results obtained and the limitations were discussed. |
| *When criteria were partially met, 0.5 points were awarded. |

**

 Table S2. PRISMA 2020 checklist of our meta-analysis**

| **Section** **and**  **Topic** | **Item** **#** | **Checklist** **item** | **Location** **where** **item** **is** **reported** |
| --- | --- | --- | --- |
| **TITLE** | | |  |
| Title | 1 | Identify the report as a systematic review. | page 1 |
| **ABSTRACT** | | | page 2 |
| Abstract | 2 | See the PRISMA 2020 for Abstracts checklist . |  |
| **INTRODUCTION** | | |  |
| Rationale | 3 | Describe the rationale for the review in the context of existing knowledge. | page 4-7 |
| Objectives | 4 | Provide an explicit statement of the objective(s) or question(s) the review addresses. | page 7 |
| **METHODS** | | |  |
| Eligibility criteria | 5 | Specify the inclusion and exclusion criteria for the review and how studies were grouped for the syntheses. | page 8-9 |
| Information  sources | 6 | Specify all databases, registers, websites, organisations, reference lists and other sources searched or consulted to identify studies. Specify the date when each source was last searched or consulted. | page 8 |
| Search strategy | 7 | Present the full search strategies for all databases, registers and websites, including any filters and limits used. | page 8-9 |
| Selection process | 8 | Specify the methods used to decide whether a study met the inclusion criteria of the review, including how many reviewers screened each record and each report retrieved, whether they worked independently, and if applicable, details of automation tools used in the process. | page 10 |
| Data collection  process | 9 | Specify the methods used to collect data from reports, including how many reviewers collected data from each report, whether they worked independently, any processes for obtaining or confirming data from study investigators, and if applicable, details of automation tools used in the process. | page 9 |
| Data items | 10a | List and define all outcomes for which data were sought. Specify whether all results that were compatible with each outcome domain in each study were sought (e.g. for all measures, time points, analyses), and if not, the methods used to decide which results to collect. | page 9-13 |
|  | 10b | List and define all other variables for which data were sought (e.g. participant and intervention characteristics, funding sources). Describe any assumptions made about any missing or unclear information. | page 9-13 |
| Study risk of bias assessment | 11 | Specify the methods used to assess risk of bias in the included studies, including details of the tool(s) used, how many reviewers assessed each study and whether they worked independently, and if applicable, details of automation tools used in the process. | page 11-12 |
| Effect measures | 12 | Specify for each outcome the effect measure(s) (e.g. risk ratio, mean difference) used in the synthesis or presentation of results. | page 11-12 |
| Synthesis  methods | 13a | Describe the processes used to decide which studies were eligible for each synthesis (e.g. tabulating the study intervention characteristics and comparing against the planned groups for each synthesis (item #5)). | page 11-12 |
|  | 13b | Describe any methods required to prepare the data for presentation or synthesis, such as handling of missing summary statistics, or data conversions. | page 13 |
|  | 13c | Describe any methods used to tabulate or visually display results of individual studies and syntheses. | page 11 |
|  | 13d | Describe any methods used to synthesize results and provide a rationale for the choice(s). If meta-analysis was performed, describe the model(s), method(s) to identify the presence and extent of statistical heterogeneity, and software package(s) used. | page 11 |
|  | 13e | Describe any methods used to explore possible causes of heterogeneity among study results (e.g. subgroup analysis, meta-regression). | page 11-12 |
|  | 13f | Describe any sensitivity analyses conducted to assess robustness of the synthesized results. | page 13 |
| Reporting bias  assessment | 14 | Describe any methods used to assess risk of bias due to missing results in a synthesis (arising from reporting biases). | page 13 |
| Certainty  assessment | 15 | Describe any methods used to assess certainty (or confidence) in the body of evidence for an outcome. | page 13 |
| Study selection | 16a | Describe the results of the search and selection process, from the number of records identified in the search to the number of studies included in the review, ideally using a flow diagram. | page 13 |
|  | 16b | Cite studies that might appear to meet the inclusion criteria, but which were excluded, and explain why they were excluded. | page 13 |
| Study  characteristics | 17 | Cite each included study and present its characteristics. | page 13 |
| Risk of bias in  studies | 18 | Present assessments of risk of bias for each included study. | page 16 |
| Results of individual studies | 19 | For all outcomes, present, for each study: (a) summary statistics for each group (where appropriate) and (b) an effect estimate and its precision (e.g. confidence/credible interval), ideally using structured tables or plots. | page 13-18 |
| Results of  syntheses | 20a | For each synthesis, briefly summarize the characteristics and risk of bias among contributing studies. | page 13-18 |
|  | 20b | Present results of all statistical syntheses conducted. If meta-analysis was done, present for each the summary estimate and its precision (e.g. confidence/credible interval) and measures of statistical heterogeneity. If comparing groups, describe the direction of the effect. | page 16 |
|  | 20c | Present results of all investigations of possible causes of heterogeneity among study results. | page 16 |
|  | 20d | Present results of all sensitivity analyses conducted to assess the robustness of the synthesized results. | page 16 |
| Reporting biases | 21 | Present assessments of risk of bias due to missing results (arising from reporting biases) for each synthesis assessed. | page 16 |
| Certainty of  evidence | 22 | Present assessments of certainty (or confidence) in the body of evidence for each outcome assessed. | page 16 |
| **DISCUSSION** | | | page 18 |
| Discussion | 23a | Provide a general interpretation of the results in the context of other evidence. | page 23 |
|  | 23b | Discuss any limitations of the evidence included in the review. | page 23 |
|  | 23c | Discuss any limitations of the review processes used. | page 23 |
|  | 23d | Discuss implications of the results for practice, policy, and future research. | page 24 |
| **OTHER** **INFORMATION** | | |  |
| Registration  and protocol | 24a | Provide registration information for the review, including register name and registration number, or state that the review was not registered. | page 8 |
|  | 24b | Indicate where the review protocol can be accessed, or state that a protocol was not prepared. |  |
|  | 24c | Describe and explain any amendments to information provided at registration or in the protocol. |  |
| Support | 25 | Describe sources of financial or non-financial support for the review, and the role of the funders or sponsors in the review. | page 24 |
| Competing  interests | 26 | Declare any competing interests of review authors. | page 24 |
| Availability of  data, code and  other materials | 27 | Report which of the following are publicly available and where they can be found: template data collection forms; data extracted from included studies; data used for all analyses; analytic code; any other materials used in the review. |  |


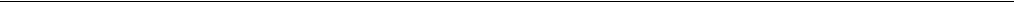


**Table S3. Demographic, clinical and imaging characteristics of the included studies of resting-state functional activity for FDRs-SCZ**

| **Study** | **Demographic characteristics** | | | | **Imaging characteristics** | | | | **Quality**  **score^** |
| --- | --- | --- | --- | --- | --- | --- | --- | --- | --- |
|  | **Participants, n** | | **Mean age, year** | | **Imaging technique*** | **Scanner** | **Software** | **Threshold** |  |
|  | **FDRs-SCZ** | **HCs** | **FDRs-SCZ** | **HCs** |  |  |  |  |  |
| Guo2014^11^ | 46 | 50 | 22.96 | 23.48 | fALFF | MRI 3.0 | DPARSF | P<0.01 GRF corrected | 10.0 |
| Guo2015^12^ | 46 | 46 | 22.96 | 23.30 | ALFF, fALFF | MRI 3.0 | REST | P<0.005 GRF corrected | 10.0 |
| Guo2017^13^ | 28 | 40 | 22.86 | 23.28 | fALFF | MRI 3.0 | DPARSF | P<0.005 GRF corrected | 10.0 |
| Liao2012^14^ | 13 | 13 | 24.92 | 25.31 | ReHo | MRI 1.5 | SPM2, | P<0.005 uncorrected | 9.5 |
| Liu2015^15^ | 27 | 27 | 25.56 | 27.44 | ALFF, ReHo | MRI 1.5 | REST | P<0.05 FWE corrected | 10.0 |
| Lui2015^16^ | 38 | 28 | 36.00 | 37.00 | ALFF | MRI 3.0 | REST | P<0.05 AlphaSim corrected | 10.0 |
| Tang2015^17^ | 28 | 44 | 20.64 | 20.55 | ALFF | MRI 3.0 | REST | P<0.05 corrected | 10.0 |
| Chen2013^18^ | 28 | 44 | 20.64 | 20.55 | ALFF | MRI 3.0 | DPARSF | P<0.05 corrected | 9.5 |
| Tian2014^19^ | 55 | 29 | 50.00 | 52.00 | ALFF | MRI 3.0 | REST | P<0.01 corrected | 10.0 |
| Wang2009^20^ | 118 | 100 | 55.73 | 40.51 | ReHo | MRI 3.0 | REST | P<0.001 uncorrected | 9.0 |
| Wang2016^21^ | 25 | 29 | 19.56 | 19.45 | ALFF | MRI 3.0 | DPARSF | P<0.05 Bonferroni corrected | 10.0 |
| Wang2018^22^ | 28 | 40 | 22.90 | 23.30 | ReHo | MRI 3.0 | REST | P<0.05 corrected | 10.0 |
| Wang2021^23^ | 23 | 115 | NA | NA | ReHo | MRI 3.0 | REST | P<0.05 GRF corrected | 8.5 |

*Abbreviations:* FDRs-SCZ, first-degree relatives of probands with schizophrenia; HCs, healthy controls; NA, not available; fMRI, functional magnetic resonance imaging; ALFF, amplitude of low-frequency fluctuation; fALFF, the fractional amplitude of low-frequency fluctuation; ReHo, Regional Homogeneity; SPM, statistical parametric mapping; FWE, family wise error; GRF, gaussian random field; DPARSF, data processing assistant for resting-State fMRI software; REST, the resting-state fMRI data analysis toolkit.

**Table S4. Demographic, clinical and imaging characteristics of the included studies of resting-state functional activity for SCZ**

| **Study** | **Demographic characteristics** | | | | **Clinical characteristics (SCZ patients)** | | | | | | **Imaging characteristics** | | | | **Quality**  **score^** |
| --- | --- | --- | --- | --- | --- | --- | --- | --- | --- | --- | --- | --- | --- | --- | --- |
|  | **Participants, n** | | **Mean age, year** | | **Illness Duration**  **(months)** | **Medication** | **FE**  **/Chronic** | **PANSS**  **total**  **score** | **PANSS**  **positive**  **score** | **PANSS**  **negative score** | **Imaging technique*** | **Scanner** | **Software** | **Threshold** |  |
|  | **SCZ** | **HCs** | **SCZ** | **HCs** |  |  |  |  |  |  |  |  |  |  |  |
| Algumaei2022^24^ | 70 | 70 | 37.9 | 35.8 | NA | NA | NA | NA | NA | NA | ALFF | MRI 3.0 | DPABI | p<0.05 FDR corrected | 8.0 |
|  | 70 | 70 | 37.9 | 35.8 | NA | NA | NA | NA | NA | NA | ALFF | MRI 3.0 | DPABI | p<0.05 FDR corrected | 8.0 |
|  | 70 | 70 | 37.9 | 35.8 | NA | NA | NA | NA | NA | NA | ALFF | MRI 3.0 | DPABI | p<0.05 FDR corrected | 8.0 |
| Alonso2017^25^ | 19 | 20 | 40.1 | 37.8 | 193.3 | Yes | Chronic | NA | 17.9 | 21.5 | ALFF | MRI 3.0 | AFNI | p<0.05 GRF corrected | 9.5 |
|  | 14 | 20 | 36.4 | 37.8 | 96.0 | Yes | Chronic | NA | 11.4 | 14.4 | ALFF | MRI 3.0 | AFNI | p<0.05 GRF corrected | 9.5 |
|  | 19 | 20 | 40.1 | 37.8 | 193.2 | Yes | Chronic | NA | 17.9 | 21.5 | fALFF | MRI 3.0 | FSL | p<0.05 GRF corrected | 9.5 |
|  | 14 | 20 | 36.4 | 37.8 | 96.0 | Yes | Chronic | NA | 11.4 | 14.4 | fALFF | MRI 3.0 | FSL | p<0.05 GRF corrected | 9.5 |
| Andreasen1997^26^ | 17 | 17 | 26.2 | 26.4 | NA | No | FE | NA | 20.2 | 21.4 | PET | PET | NA | NA | 8.5 |
| Bai2016^27^ | 17 | 17 | 26.0 | 28.7 | 40.3 | No | NA | 82.1 | 15.3 | 24.9 | ReHo | MRI 1.5 | SPM8 | p<0.05 AlphaSim corrected | 10.0 |
| Bai2018^28^ | 78 | 32 | 30.9 | 30.7 | NA | NA | NA | NA | NA | NA | ALFF | MRI 3.0 | SPM | p<0.05 AlphaSim corrected | 8.5 |
| Blackwood1999^29^ | 19 | 34 | 32.3 | 39.5 | NA | Yes | NA | NA | NA | NA | SPECT | SPECT | SPMs | p<0.0005 uncorrected | 8.5 |
| Boyer2012^30^ | 31 | 18 | 32.2 | 32.3 | 135.6 | Yes | Chronic | 63.6 | 15.1 | 23.3 | SPECT | SPECT | SPM8 | p<0.05 corrected | 9.5 |
| Cai2021^31^ | 72 | 74 | 36.6 | 36.1 | NA | Yes | NA | 54.8 | 14.6 | 12.6 | ALFF | MRI 3.0 | DPARSF, SPM8 | p<0.05 GRF corrected | 9.0 |
| Chen2015^32^ | 49 | 81 | 24.8 | 24.7 | 48.0 | Yes | Chronic | 86.0 | NA | NA | fALFF | MRI 3.0 | SPM8 | p<0.05 AlphaSim corrected | 9.0 |
|  | 23 | 81 | 24.7 | 24.7 | 41.0 | Yes | NA | 89.0 | NA | NA | fALFF | MRI 3.0 | SPM8 | p<0.05 AlphaSim corrected | 9.0 |
| Chen2017^33^ | 21 | 25 | 26.7 | 25.8 | 6.2 | No | FE | 83.5 | 21.8 | 20.1 | ALFF | MRI 3.0 | DPARSF | NA | 9.5 |
| Chen2022^34^ | 56 | 27 | 23.6 | 21.9 | 9.8 | No | FE | 107.9 | 15.4 | 24.6 | fALFF | MRI 3.0 | DPABI | p<0.05 TFCE corrected | 10.0 |
| Cheng2017^35^ | 19 | 20 | 26.8 | 26.2 | 40.4 | NA | NA | 71.5 | 25.0 | 14.5 | ASL | MRI 3.0 | SPM8 | p<0.05 AlphaSim corrected | 9.5 |
|  | 25 | 20 | 24.5 | 26.2 | 35.3 | NA | NA | 79.1 | 13.1 | 29.2 | ASL | MRI 3.0 | SPM8 | p<0.05 AlphaSim corrected | 9.5 |
| Cheng2017a^36^ | 32 | 37 | 26.9 | 29.5 | 11.3 | No | FE | 85.3 | NA | NA | fALFF | MRI 3.0 | SPM12 | p<0.05 AlphaSim corrected | 8.5 |
| Clark2001^37^ | 26 | 32 | 26.6 | 29.2 | NA | No | NA | NA | NA | NA | PET | PET | NA | Bonferroni corrected | 8.5 |
| Cui2016^38^ | 40 | 40 | 27.9 | 28.3 | NA | NA | NA | NA | NA | NA | ALFF | MRI 3.0 | DPARSF, SPM8 | p<0.05 AlphaSim corrected | 8.5 |
| Cui2016a^39^ | 17 | 19 | 21.2 | 23.8 | 6.5 | No | FE | 106.2 | 31.1 | 25.5 | ReHo | MRI 3.0 | DPARSFA | p<0.01 AlphaSim corrected | 10.0 |
|  | 15 | 19 | 22.5 | 23.8 | 10.2 | No | FE | 88.1 | 17.9 | 22.7 | ReHo | MRI 3.0 | DPARSFA | p<0.01 AlphaSim corrected | 10.0 |
|  | 17 | 19 | 21.2 | 23.8 | 6.5 | No | FE | 106.2 | 31.1 | 25.5 | ALFF | MRI 3.0 | DPARSFA | p<0.01 AlphaSim corrected | 10.0 |
|  | 15 | 19 | 22.5 | 23.8 | 10.2 | No | FE | 88.1 | 17.9 | 22.7 | ALFF | MRI 3.0 | DPARSFA | p<0.01 AlphaSim corrected | 10.0 |
| Cui2017^40^ | 45 | 51 | 26.0 | 27.0 | NA | No | FE | 96.0 | 24.0 | 23.0 | ASL | MRI 3.0 | SPM12 | p<0.005 AlphaSim corrected | 9.5 |
| Cui2017a^41^ | 25 | 25 | 24.0 | 26.0 | 18.0 | Yes | NA | 92.0 | 21.0 | 23.0 | ASL | MRI 3.0 | SPM12 | p<0.001 AlphaSim corrected | 10.0 |
| Deng2018^42^ | 70 | 84 | 20.0 | 21.9 | 5.7 | Yes | FE | NA | NA | NA | ReHo | MRI 3.0 | DPARSF, SPM8 | p<0.005 AlphaSim corrected | 8.5 |
| Deng2019^43^ | 69 | 74 | 24.2 | 26.3 | 13.7 | No | FE | 84.2 | 24.4 | 17.6 | ALFF | MRI 3.0 | DPABI | p<0.05 GRF corrected | 9.5 |
| Deng2023^44^ | 84 | 94 | 22.6 | 23.9 | 12.4 | Yes | FE | 90.8 | 14.8 | 20.9 | ALFF | MRI 3.0 | DPABI | p<0.05 GRF corrected | 10.0 |
|  | 84 | 94 | 22.6 | 23.9 | 12.4 | Yes | FE | 90.8 | 14.8 | 20.9 | ALFF | MRI 3.0 | DPABI | p<0.05 GRF corrected | 10.0 |
|  | 84 | 94 | 22.6 | 23.9 | 12.4 | Yes | FE | 90.8 | 14.8 | 20.9 | ReHo | MRI 3.0 | DPABI | p<0.05 GRF corrected | 10.0 |
|  | 84 | 94 | 22.6 | 23.9 | 12.4 | Yes | FE | 90.8 | 14.8 | 20.9 | ReHo | MRI 3.0 | DPABI | p<0.05 GRF corrected | 10.0 |
| Dong2019^45^ | 96 | 122 | 39.8 | 38.0 | 182.2 | Yes | Chronic | 62.4 | 13.4 | 20.7 | ReHo | MRI 3.0 | DPABI | p<0.05 FDR corrected | 10.0 |
| Dye1999^46^ | 6 | 10 | 48.8 | 51.5 | NA | Yes | NA | NA | NA | NA | PET | PET | SPM95 | p<0.05 corrected | 8.5 |
| Faget-Agius2012^47^ | 31 | 18 | 32.2 | 32.3 | 135.6 | Yes | Chronic | 63.6 | 15.1 | 23.3 | SPECT | SPECT | SPM5 | p<0.05 uncorrected | 9.5 |
| Fang2021^48^ | 35 | 34 | 22.3 | 21.4 | 10.7 | No | FE | 89.3 | 22.8 | 19.8 | fALFF | MRI 3.0 | REST | p<0.05 TFCE-FWE corrected | 10.0 |
|  | 34 | 34 | 22.7 | 21.4 | 13.6 | No | FE | 88.9 | 23.4 | 21.8 | fALFF | MRI 3.0 | REST | p<0.05 TFCE-FWE corrected | 10.0 |
|  | 35 | 34 | 22.3 | 21.4 | 10.7 | No | FE | 89.3 | 22.8 | 19.8 | fALFF | MRI 3.0 | REST | p<0.05 TFCE-FWE corrected | 10.0 |
|  | 34 | 34 | 22.7 | 21.4 | 13.6 | No | FE | 88.9 | 23.4 | 21.8 | fALFF | MRI 3.0 | REST | p<0.05 TFCE-FWE corrected | 10.0 |
|  | 35 | 34 | 22.3 | 21.4 | 10.7 | No | FE | 89.3 | 22.8 | 19.8 | ReHo | MRI 3.0 | REST | p<0.05 TFCE-FWE corrected | 10.0 |
|  | 34 | 34 | 22.7 | 21.4 | 13.6 | No | FE | 88.9 | 23.4 | 21.8 | ReHo | MRI 3.0 | REST | p<0.05 TFCE-FWE corrected | 10.0 |
| Feng2022^49^ | 34 | 34 | 31.0 | 28.3 | NA | No | FE | 85.7 | 23.5 | 19.9 | fALFF | MRI 3.0 | SPM12 | p<0.05 uncorrected | 9.5 |
| Foucher2018^50^ | 9 | 27 | 38.0 | 39.0 | 172.8 | Yes | Chronic | 66.7 | 16.8 | 16.8 | ASL | MRI 3.0 | SPM12 | p<0.05 uncorrected | 9.5 |
|  | 20 | 27 | 38.0 | 39.0 | 187.2 | Yes | Chronic | 65.7 | 11.7 | 19.6 | ASL | MRI 3.0 | SPM12 | p<0.05 uncorrected | 9.5 |
| Gao2015^51^ | 14 | 14 | 33.2 | 34.9 | 110.4 | Yes | Chronic | 74.1 | 16.4 | 22.6 | ReHo | MRI 1.5 | DPARSF, REST 1.5 | p<0.05 GRF corrected | 10.0 |
| Gao2018^52^ | 17 | 29 | 36.8 | 32.7 | 94.6 | Yes | Chronic | 37.3 | 9.5 | 8.4 | ReHo | MRI 3.0 | SPM8 | p<0.05 GRF corrected | 10.0 |
|  | 17 | 29 | 31.2 | 32.7 | 168.0 | Yes | Chronic | 97.8 | 27.5 | 21.1 | ReHo | MRI 3.0 | SPM8 | p<0.05 GRF corrected | 10.0 |
| Gao2020^53^ | 57 | 50 | 31.6 | 28.4 | 30.2 | No | FE | 91.8 | 26.4 | 20.7 | ReHo | MRI 3.0 | SPM12 | p<0.05 GRF corrected | 10.0 |
| Gao2022^54^ | 131 | 128 | 26.3 | 30.3 | 83.0 | NA | Chronic | 89.6 | NA | NA | ALFF | MRI 3.0 | REST | p<0.01 GRF corrected | 9.0 |
| Guo2018^55^ | 49 | 50 | 22.7 | 23.5 | 22.5 | No | FE | 91.3 | 22.3 | 22.8 | ALFF | MRI 3.0 | SPM8 | p<0.05 GRF corrected | 9.5 |
| Hare2017^56^ | 143 | 155 | 38.7 | 37.8 | 209.1 | Yes | Chronic | 57.6 | 15.3 | 14.2 | ALFF | MRI 3.0 | AFNI | p<0.05 FWE corrected | 10.0 |
|  | 143 | 155 | 38.7 | 37.8 | 209.1 | Yes | Chronic | 57.6 | 15.3 | 14.2 | ALFF | MRI 3.0 | AFNI | p<0.05 FWE corrected | 10.0 |
| Horacek2004^57^ | 67 | 18 | 28.3 | 32.3 | 46.9 | Yes | FE | 64.6 | 14.6 | 16.5 | PET | PET | SPM99 | p<0.05 corrected | 9.5 |
| Horga2014^58^ | 9 | 8 | 25.3 | 28.1 | 24.0 | Yes | NA | 92.0 | 26.3 | 20.7 | PET | PET | SPM5 | p<0.05 FWE corrected | 9.0 |
| Hu2016^59^ | 42 | 38 | 24.9 | 24.8 | 8.4 | No | FE | 91.9 | 25.6 | 18.2 | fALFF | MRI 3.0 | SPM | p<0.05 AlphaSim corrected | 9.5 |
|  | 42 | 38 | 24.9 | 24.8 | 8.4 | No | FE | 91.9 | 25.6 | 18.2 | ReHo | MRI 3.0 | DPARSFA | p<0.05 AlphaSim corrected | 9.5 |
| Huang2010^60^ | 66 | 66 | 24.2 | 24.5 | 8.8 | No | FE | 107.2 | 26.4 | 20.7 | ALFF | MRI 3.0 | SPM2 | p<0.05 corrected | 9.5 |
| Huang2022^61^ | 187 | 100 | 25.3 | 25.3 | 13.1 | No | FE | 91.9 | 22.7 | 24.2 | ReHo | MRI 3.0 | RESTplus | p<0.05 FWE corrected | 10.0 |
| Jin2021^62^ | 23 | 24 | 31.7 | 30.9 | NA | No | FE | NA | NA | NA | ReHo | MRI 3.0 | SPM8 | p<0.05 GRF corrected | 9.0 |
| Jing2023^63^ | 56 | 51 | 41.8 | 41.0 | NA | NA | NA | 72.2 | 15.1 | 23.2 | ReHo | MRI 3.0 | DPARSFA | p<0.05 GRF corrected | 10.0 |
| Kanahara2009^64^ | 23 | 22 | 38.0 | 36.1 | 186.0 | Yes | Chronic | NA | NA | NA | SPECT | SPECT | iSSP | p<0.001 uncorrected | 8.5 |
|  | 18 | 18 | 27.0 | 26.4 | 15.6 | Yes | FE | NA | NA | NA | SPECT | SPECT | iSSP | p<0.001 uncorrected | 8.5 |
| Kanahara2013^65^ | 33 | 45 | 35.0 | 34.8 | 156.0 | Yes | Chronic | NA | NA | NA | SPECT | SPECT | SPM5 | p<0.05 FWE corrected | 8.5 |
|  | 40 | 45 | 31.2 | 34.8 | 88.8 | Yes | Chronic | NA | NA | NA | SPECT | SPECT | SPM5 | p<0.05 FWE corrected | 8.5 |
| Kawakami2014^66^ | 44 | 37 | 40.0 | 37.5 | 63.6 | Yes | Chronic | 42.3 | NA | NA | SPECT | SPECT | SPM8 | p<0.05 uncorrected | 8.5 |
| Kim2000^67^ | 30 | 30 | 33.0 | 30.1 | 122.4 | No | Chronic | NA | NA | NA | PET | PET | Brain Research | NA | 8.5 |
| Kindler2018^68^ | 12 | 18 | 20.7 | 19.0 | NA | No | FE | NA | NA | NA | ASL | MRI 3.0 | SPM8 | p<0.05 FWE corrected | 9.0 |
| Kuang2019^69^ | 28 | 33 | 25.1 | 24.4 | 8.7 | Yes | FE | 65.1 | NA | NA | fALFF | MRI 3.0 | SPM12 | p<0.01 FDR corrected | 9.0 |
| Kuang2022^70^ | 28 | 33 | 25.1 | 24.2 | 8.7 | Yes | FE | 65.1 | 17.4 | 13.6 | ALFF | MRI 3.0 | RESTplus | p<0.05 AlphaSim corrected | 10.0 |
| Lei2015^71^ | 124 | 102 | 24.5 | 24.8 | 8.2 | No | FE | 87.6 | 14.7 | 16.6 | ALFF | MRI 3.0 | DPARSF | p<0.05 AlphaSim corrected | 9.5 |
| Li2016^72^ | 20 | 16 | 22.9 | 22.4 | 6.4 | No | FE | 101.6 | 25.1 | 18.8 | ALFF | MRI 3.0 | DPARSF, SPM8 | p<0.05 AlphaSim corrected | 10.0 |
| Li2020^73^ | 38 | 35 | 24.2 | NA | NA | No | FE | 86.5 | 23.6 | 20.9 | fALFF | MRI 3.0 | DPABI | p<0.05 GRF corrected | 9 |
| Li2021^74^ | 31 | 33 | 25.0 | 24.2 | 8.5 | NA | FE | 65.6 | NA | NA | ALFF | MRI 3.0 | SPM12 | p<0.01 AlphaSim corrected | 9.0 |
| Li2023^75^ | 88 | 81 | 28.8 | 30.9 | 74.8 | Yes | Chronic | 93.2 | 13.2 | 25.8 | ALFF | MRI 3.0 | RESTplus | p<0.05 FDR corrected | 10.0 |
|  | 88 | 81 | 28.8 | 30.9 | 74.8 | Yes | Chronic | 93.2 | 13.2 | 25.8 | ReHo | MRI 3.0 | RESTplus | p<0.05 FDR corrected | 10.0 |
| Lian2018^76^ | 18 | 30 | 20.4 | 20.5 | 7.9 | NA | FE | 80.5 | 19.1 | 21.5 | fALFF | MRI 3.0 | SPM8 | p<0.01 FDR corrected | 9.5 |
| Liang2013^77^ | 20 | 20 | 31.2 | 29.6 | 73.6 | Yes | Chronic | 76.0 | NA | NA | ReHo | MRI 3.0 | SPM8 | p<0.01 AlphaSim corrected | 8.5 |
| Liang2014^78^ | 36 | 19 | 33.2 | 30.0 | 100.2 | Yes | Chronic | 80.4 | 20.0 | 15.8 | ALFF | MRI 3.0 | DPARSF, SPM8 | p<0.01 AlphaSim corrected | 9.5 |
| Liu2006^79^ | 18 | 18 | 23.7 | 24.4 | 26.8 | NA | NA | 80.4 | NA | NA | ReHo | MRI 1.5 | SPM2 | p<0.05 AlphaSim corrected | 8.5 |
| Liu2010^80^ | 13 | 18 | 24.1 | 25.4 | 27.3 | No | NA | NA | 79.5 | 79.5 | ALFF | MRI 3.0 | SPM5 | p<0.001 uncorrected | 9.0 |
| Liu2016^15^ | 27 | 27 | 25.4 | 27.4 | 18.3 | Yes | FE | 85.8 | 21.6 | 23.2 | ReHo | MRI 1.5 | DPARSF, SPM8 | p<0.05 FWE corrected | 9.5 |
|  | 27 | 27 | 25.4 | 27.4 | 18.3 | Yes | FE | 85.8 | 21.6 | 23.2 | ALFF | MRI 1.5 | REST | p<0.05 FWE corrected | 10.0 |
| Liu2017^81^ | 60 | 32 | 32.6 | 33.0 | 20.9 | Yes | FE | 91.7 | NA | NA | ReHo | MRI 3.0 | SPM8 | p<0.05 AlphaSim corrected | 9.0 |
| Lui2010^82^ | 34 | 34 | 24.6 | 25.0 | 7.8 | No | FE | 104.2 | 26.9 | 19.1 | ALFF | MRI 3.0 | SPM2, REST | p<0.05 AlphaSim corrected | 9.5 |
| Lui2015^16^ | 37 | 59 | 36.0 | 34.0 | 177.7 | Yes | Chronic | 71.2 | 18.2 | 18.3 | ALFF | MRI 3.0 | SPM8 | p<0.05 FWE corrected | 10.0 |
| Ma2023^83^ | 44 | 40 | 20.5 | 19.0 | NA | No | FE | 83.3 | 20.8 | 20.0 | fALFF | MRI 3.0 | DPARSF, SPM8 | p<0.05 FDR corrected | 10.0 |
|  | 44 | 40 | 20.5 | 19.0 | NA | No | FE | 83.3 | 20.8 | 20.0 | ReHo | MRI 3.0 | DPARSF, SPM8 | p<0.05 FDR corrected | 10.0 |
| Malaspina2004^84^ | 10 | 9 | 37.4 | 29.9 | NA | NA | NA | NA | 14.1 | 24.3 | SPECT | SPECT | SPM96 | p<0.05 corrected | 8.5 |
|  | 16 | 9 | 29.6 | 29.9 | NA | NA | NA | NA | 11.6 | 17.0 | SPECT | SPECT | SPM96 | p<0.05 corrected | 8.5 |
| Mathew1982^85^ | 23 | 18 | 26.9 | 31.2 | NA | Yes | Chronic | NA | NA | NA | SPECT | SPECT | NA | NA | 8.5 |
| Mauri2008^86^ | 9 | 9 | 38.4 | 40.0 | 172.0 | Yes | Chronic | NA | 24.2 | 26.6 | SPECT | SPECT | SPM99 | p<0.001 uncorrected | 9.5 |
| Ota2014^87^ | 36 | 42 | 39.8 | 37.9 | 189.8 | Yes | Chronic | 61.8 | 15.3 | 14.9 | ASL | MRI 3.0 | SPM5 | p<0.05 FWE corrected | 9.5 |
| Park2009^88^ | 29 | 21 | 29.8 | 30.1 | 90.0 | Yes | Chronic | NA | 15.9 | 16.7 | PET | PET | SPM2 | p<0.05 corrected | 9.5 |
| Park2019^89^ | 16 | 15 | 34.4 | 34.3 | NA | No | FE | NA | NA | NA | PET | PET | SPM12 | p<0.001 uncorrected | 8.5 |
| Potkic2002^90^ | 7 | 7 | 38.6 | 38.0 | 174.0 | Yes | Chronic | NA | NA | NA | PET | PET | SPM99 | p<0.025 uncorrected | 8.5 |
|  | 7 | 7 | 40.6 | 38.0 | 226.8 | Yes | Chronic | NA | NA | NA | PET | PET | SPM99 | p<0.025 uncorrected | 8.5 |
| Ren2013^91^ | 100 | 100 | 24.3 | 24.4 | 6.3 | No | FE | 97.9 | 25.1 | 18.8 | ALFF | MRI 3.0 | DPARSF | p<0.05 AlphaSim corrected | 9.5 |
| Salvador2017^92^ | 116 | 122 | 36.8 | 36.5 | 178.6 | Yes | Chronic | 69.2 | 16.6 | 19.6 | ALFF | MRI 1.5 | FSL | p<0.05 FWE corrected | 9.5 |
| Scheef2010^93^ | 11 | 25 | NA | NA | NA | No | NA | NA | 20.2 | 21.4 | ASL | MRI 3.0 | SPM2 | p<0.05 FDR corrected | 8.5 |
| Schneider2019^94^ | 29 | 20 | 27.7 | 30.6 | NA | Yes | NA | 47.7 | 6.4 | 12.0 | ASL | MRI 3.0 | SPM12 | NA | 8.5 |
| Shan2020^95^ | 39 | 20 | 24.4 | 25.7 | NA | No | FE | NA | NA | NA | ReHo | MRI 3.0 | DPABI | p<0.05 FDR corrected | 8.5 |
| Shan2021^96^ | 39 | 20 | 24.4 | 25.7 | NA | Yes | NA | NA | NA | NA | ReHo | MRI 3.0 | REST | p<0.05 FDR corrected | 8.0 |
| Shao2020^97^ | 155 | 97 | NA | NA | NA | NA | NA | NA | NA | NA | ALFF | MRI 3.0 | DPABI | p<0.05 FWE corrected | 9.0 |
| Suazo2013^98^ | 23 | 29 | 38.4 | 34.0 | 83.8 | Yes | Chronic | 74.7 | 19.8 | 20.1 | SPECT | SPECT | SPM | p<0.001 uncorrected | 9.5 |
| Sui2015^99^ | 47 | 50 | 35.3 | 36.7 | NA | Yes | NA | NA | 15.4 | 15.1 | fALFF | MRI 3.0 | SPM8 | p<0.05 FDR corrected | 8.5 |
| Sun2020^100^ | 26 | 26 | 32.2 | 33.0 | NA | NA | NA | NA | NA | NA | ALFF | MRI 3.0 | SPM8 | p<0.001 FDR corrected | 8.0 |
| Sun2021^101^ | 40 | 42 | 28.2 | 29.2 | 7.9 | No | FE | NA | NA | NA | ReHo | MRI 3.0 | SPM8 | p<0.05 GRF corrected | 9.5 |
| Tan2017^102^ | 32 | 32 | 29.4 | 30.0 | NA | NA | NA | NA | NA | NA | ALFF | MRI 3.0 | DPARSF, SPM8 | p<0.05 AlphaSim corrected | 8.5 |
| Tang2019^103^ | 42 | 59 | 19.0 | 20.9 | NA | Yes | FE | NA | NA | NA | ALFF | MRI NA | SPM8 | p<0.01 uncorrected | 8.5 |
| Tian2018^104^ | 44 | 33 | 24.4 | 23.6 | NA | NA | NA | NA | NA | NA | ALFF | MRI 3.0 | SPM8 | p<0.05 AlphaSim corrected | 8.5 |
| Tong2022^105^ | 30 | 43 | 30.9 | 32.3 | 8.5 | Yes | FE | 77.5 | 20.0 | 20.7 | ReHo | MRI 3.0 | REST | p<0.001 uncorrected | 10.0 |
| Tong2023^106^ | 131 | 128 | 26.3 | 30.3 | 6.9 | Yes | Chronic | 89.6 | NA | NA | ALFF | MRI 3.0 | DPARSF | p<0.05 corrected | 9.5 |
| Turner2013^107^ | 146 | 160 | 38.0 | 37.0 | 205.1 | Yes | Chronic | 57.6 | 15.0 | 14.4 | fALFF | MRI 3.0 | SPM5 | p<0.05 FDR corrected | 9.5 |
|  | 146 | 160 | 38.0 | 37.0 | 205.1 | Yes | Chronic | 57.6 | 15.0 | 14.4 | ALFF | MRI 3.0 | SPM5 | p<0.05 FDR corrected | 9.5 |
| Wake2010^108^ | 30 | 37 | 36.5 | 37.5 | 15.6 | Yes | FE | 42.3 | NA | NA | SPECT | SPECT | SPM2 | p<0.001 uncorrected | 8.5 |
| Wake2016^109^ | 43 | 27 | 33.8 | 32.3 | 63.6 | Yes | Chronic | NA | 76.2 | 80.3 | SPECT | SPECT | SPM8 | p<0.05 corrected | 9.0 |
|  | 19 | 10 | 55.2 | 54.5 | 98.4 | Yes | Chronic | NA | 72.3 | 77.1 | SPECT | SPECT | SPM8 | p<0.05 corrected | 9.0 |
| Walther2011^110^ | 11 | 14 | 35.4 | 31.7 | 107.2 | Yes | Chronic | 54.3 | 11.7 | 18.0 | ASL | MRI 3.0 | SPM8 | p<0.01 FDR corrected | 9.5 |
| Walther2016^111^ | 42 | 41 | 35.9 | 38.6 | NA | Yes | NA | 77.1 | 16.0 | 21.9 | ASL | MRI 3.0 | SPM8 | p<0.001 FWE corrected | 8.5 |
| Wang2021^112^ | 31 | 115 | NA | NA | NA | NA | NA | NA | NA | NA | ReHo | MRI 3.0 | REST | p<0.05 GRF corrected | 8.5 |
| Wang2009^113^ | 24 | 39 | 35.7 | 34.1 | 133.7 | Yes | Chronic | 97.4 | 26.2 | 23.8 | ReHo | MRI 1.5 | SPM2 | p<0.001 uncorrected | 10.0 |
| Wang2019^114^ | 14 | 14 | 34.9 | 33.2 | 110.4 | Yes | Chronic | 74.1 | 16.4 | 22.6 | ALFF | MRI 1.5 | REST1.8 | p<0.05 AlphaSim corrected | 9.5 |
| Wei2018^115^ | 126 | 188 | 24.9 | 26.6 | 52.8 | Yes | Chronic | NA | NA | NA | ReHo | MRI 3.0 | DPARSF | p<0.05 AlphaSim corrected | 8.5 |
| Wu2018^116^ | 39 | 56 | 30.0 | 25.1 | NA | Yes | Chronic | 84.9 | 23.4 | 22.4 | ReHo | MRI 3.0 | SPM8 | p<0.05 FDR corrected | 9.5 |
|  | 43 | 56 | 26.4 | 25.1 | NA | No | FE | 87.1 | 25.2 | 21.4 | ReHo | MRI 3.0 | SPM8 | p<0.05 FDR corrected | 9.5 |
|  | 43 | 56 | 26.4 | 25.1 | NA | No | FE | 87.1 | 25.2 | 21.4 | ALFF | MRI 3.0 | SPM8 | p<0.05 FDR corrected | 9.5 |
|  | 39 | 56 | 30.0 | 25.1 | NA | Yes | Chronic | 84.9 | 23.4 | 22.4 | ALFF | MRI 3.0 | SPM8 | p<0.05 FDR corrected | 9.5 |
| Wu2019^117^ | 32 | 32 | 30.9 | 31.4 | 8.9 | No | FE | NA | NA | NA | fALFF | MRI 3.0 | SPM8 | p<0.05 GRF corrected | 8.5 |
| Xie2021^118^ | 30 | 33 | 30.3 | 32.0 | 21.4 | Yes | FE | 79.85 | 19.7 | NA | ALFF | MRI 3.0T | SPM8 | p<0.05 FDR corrected | 9.5 |
| Xie2023^119^ | 26 | 28 | 29.9 | 28.3 | 20.1 | NA | NA | 78.8 | 19.4 | 19.5 | ASL | MRI 3.0 | SPM 12 | p<0.05 GRF corrected | 10.0 |
| Xu2015^120^ | 24 | 21 | 31.9 | 34.1 | 166.8 | Yes | Chronic | 98.2 | 2.9 | 24.0 | ReHo | MRI 3.0 | SPM8 | P<0.001 uncorrected | 10.0 |
| Xu2017^121^ | 100 | 95 | 34.1 | 33.3 | 126.3 | Yes | Chronic | NA | 16.9 | 20.2 | ASL | MRI 3.0 | SPM8 | p<0.05 FWE corrected | 9.5 |
| Xu2022^122^ | 92 | 60 | 70.3 | 71.3 | 20.9 | Yes | FE | 80.8 | 15.0 | 19.1 | ALFF | MRI 3.0 | NA | p<0.05 uncorrected | 8.5 |
|  | 92 | 60 | 70.3 | 71.3 | 20.9 | Yes | FE | 80.8 | 15.0 | 19.1 | ReHo | MRI 3.0 | NA | p<0.05 uncorrected | 8.5 |
| Yan2020^123^ | 69 | 74 | 24.2 | 26.3 | 13.7 | No | FE | 84.2 | 24.4 | 17.6 | ReHo | MRI 3.0 | DPARSF | p<0.05 TFCE corrected | 10.0 |
| Yang2014^124^ | 9 | 9 | NA | NA | 209.3 | Yes | Chronic | 83.9 | 14.8 | 27.7 | ALFF | MRI 3.0 | DPARSF, SPM8 | p<0.05 AlphaSim corrected | 9.0 |
|  | 9 | 9 | NA | NA | 209.3 | Yes | Chronic | 83.9 | 14.8 | 27.7 | ReHo | MRI 3.0 | DPARSF, SPM8 | p<0.05 AlphaSim corrected | 9.0 |
| Yang2021^125^ | 17 | 30 | 31.1 | 34.6 | 64.8 | Yes | Chronic | 69.8 | 19.6 | 15.3 | ReHo | MRI 3.0 | SPM12 | p<0.05 GRF corrected | 10.0 |
| Yang2021a^126^ | 37 | 39 | 39.7 | 40.9 | 204.0 | Yes | Chronic | 76 | 13.6 | 23.9 | ReHo | MRI 3.0 | REST | p<0.05 corrected | 10.0 |
| Yang2021b^127^ | 28 | 27 | 33.7 | 30.2 | NA | Yes | Chronic | 57.5 | NA | NA | ALFF | MRI 3.0 | RESTplus | p<0.01 GRF corrected | 9.0 |
| Yang2022^128^ | 39 | 31 | 34.0 | 32.0 | 115.2 | NA | Chronic | 52.6 | 11.8 | 15.3 | ALFF | MRI 3.0 | SPM12 | p<0.05 FWE corrected | 9.5 |
|  | 39 | 31 | 34.0 | 32.0 | 115.2 | NA | Chronic | 52.6 | 11.8 | 15.3 | ALFF | MRI 3.0 | SPM12 | p<0.05 FWE corrected | 9.5 |
|  | 39 | 31 | 34.0 | 32.0 | 115.2 | NA | Chronic | 52.6 | 11.8 | 15.3 | fALFF | MRI 3.0 | SPM12 | p<0.05 FWE corrected | 9.5 |
|  | 39 | 31 | 34.0 | 32.0 | 115.2 | NA | Chronic | 52.6 | 11.8 | 15.3 | fALFF | MRI 3.0 | SPM12 | p<0.05 FWE corrected | 9.5 |
| Yang2022a^129^ | 57 | 138 | 25.9 | 31.0 | NA | Yes | FE | 81.5 | 23.0 | 18.5 | ReHo | MRI 3.0 | RESTplus | p<0.05 AlphaSim corrected | 9.5 |
|  | 51 | 138 | 27.2 | 31.0 | NA | Yes | Chronic | 82.7 | 23.4 | 19.4 | ReHo | MRI 3.0 | RESTplus | p<0.05 AlphaSim corrected | 9.5 |
| Yang2023^130^ | 20 | 19 | 48.6 | 43.2 | NA | Yes | Chronic | 68.5 | 12.0 | 22.2 | ReHo | MRI 3.0 | DPABI | p<0.05 GRF corrected | 10.0 |
| Yin2021^131^ | 30 | 30 | 19.8 | 20.8 | 8.5 | Yes | FE | 78.3 | 21.8 | 18.8 | ReHo | MRI 3.0 | REST | p<0.001 uncorrected | 10.0 |
|  | 30 | 30 | 19.8 | 20.8 | 8.5 | Yes | FE | 78.3 | 21.8 | 18.8 | ALFF | MRI 3.0 | REST | p<0.001 uncorrected | 10.0 |
| Yu2013^132^ | 69 | 62 | 31.7 | 29.9 | 85.2 | Yes | Chronic | 52.9 | 12.1 | 13.4 | ReHo | MRI 3.0 | SPM | p<0.05 FWE corrected | 10.0 |
| Yu2014^133^ | 69 | 62 | 31.7 | 29.9 | 85.2 | Yes | Chronic | 52.9 | 12.1 | 13.4 | fALFF | MRI 3.0 | SPM5 | p<0.05 FWE corrected | 10.0 |
|  | 69 | 62 | 31.7 | 29.9 | 85.2 | Yes | Chronic | 52.9 | 12.1 | 13.4 | ALFF | MRI 3.0 | SPM5 | p<0.05 FWE corrected | 10.0 |
| Yu2021^134^ | 22 | 60 | 33.4 | 32.9 | 15.5 | No | FE | 93.5 | 27.2 | 19.8 | ALFF | MRI 3.0 | SPM12 | p<0.05 FWE corrected | 10.0 |
|  | 22 | 60 | 33.4 | 32.9 | 15.5 | No | FE | 93.5 | 27.2 | 19.8 | ReHo | MRI 3.0 | SPM12 | p<0.05 FWE corrected | 10.0 |
| Yu2021a^135^ | 22 | 60 | 33.4 | 32.9 | 15.5 | No | FE | 93.5 | 27.2 | 19.8 | fALFF | MRI 3.0 | SPM12 | p<0.05 FWE corrected | 10.0 |
| Yu2021b^136^ | 31 | 31 | 30.6 | 26.4 | 46.7 | No | FE | 64.1 | 15.4 | 15.9 | fALFF | MRI 3.0 | DPARSF | p<0.05 FWE corrected | 10.0 |
| Zhang2017^137^ | 22 | 23 | 28.0 | 28.0 | NA | NA | NA | 79.0 | NA | NA | ReHo | MRI 3.0 | SPM12 | p<0.001 uncorrected | 8.5 |
| Zhang2018^138^ | 40 | 40 | 38.5 | 37.9 | NA | No | NA | 94.6 | 30.1 | 27.1 | ALFF | MRI 3.0 | SPM8 | p<0.05 GRF corrected | 9.0 |
| Zhang2018a^139^ | 26 | 30 | 45.5 | 46.4 | 267.6 | Yes | Chronic | 62.7 | 13.9 | 17.9 | fALFF | MRI 3.0 | SPM8 | p<0.01 AlphaSim corrected | 9.5 |
|  | 26 | 30 | 45.6 | 46.4 | 262.8 | Yes | Chronic | 68.5 | 15.4 | 21.9 | fALFF | MRI 3.0 | SPM8 | p<0.01 AlphaSim corrected | 9.5 |
| Zhang2018b^140^ | 16 | 18 | 23.8 | 23.2 | NA | NA | NA | 76.0 | 14.2 | 18.7 | ReHo | MRI 3.0 | SPM8 | p<0.001 uncorrected | 8.5 |
| Zhang2018c^141^ | 28 | 38 | 21.9 | 24.1 | NA | No | FE | 82.9 | 22.0 | 19.1 | ReHo | MRI 3.0 | DPABI | p<0.05 FWE corrected | 9.5 |
| Zhang2018d^142^ | 31 | 32 | 45.7 | 45.4 | 273.6 | Yes | Chronic | 68.0 | 14.0 | 22.0 | ALFF | MRI 3.0 | SPM5 | p<0.05 AlphaSim corrected | 10.0 |
|  | 32 | 32 | 47.2 | 45.4 | 271.2 | Yes | Chronic | 62.0 | 14.0 | 17.0 | ALFF | MRI 3.0 | SPM5 | p<0.05 AlphaSim corrected | 10.0 |
| Zhang2019^143^ | 55 | 27 | 23.8 | 21.9 | 10.1 | No | FE | 105.4 | 15.6 | 24.2 | fALFF | MRI 3.0 | SPM8 | p<0.05 TFCE corrected | 9.5 |
| Zhao2018^144^ | 58 | 39 | 20.4 | 24.1 | 24.0 | No | FE | 86.0 | 23.6 | 21.4 | ReHo | MRI 3.0 | DPARSF | p<0.001 uncorrected | 9.5 |
| Zhao2018a^145^ | 44 | 26 | 23.7 | 22.6 | 12.0 | No | FE | 102.0 | 15.3 | 24.7 | ReHo | MRI 3.0 | DPARSF | p<0.01 TFCE corrected | 9.5 |
| Zhou2017^146#^ | 41 | 50 | 32.3 | 32.0 | 118.8 | Yes | Chronic | 68.4 | 14.8 | 20.2 | ASL | MRI 3.0 | SPM8 | p<0.05 FDR corrected | 9.5 |
|  | 35 | 50 | 31.5 | 32.0 | 101.4 | Yes | Chronic | 73.2 | 20.1 | 18.8 | ASL | MRI 3.0 | SPM8 | p<0.05 FDR corrected | 9.5 |
| Zhou2019^147#^ | 33 | 40 | 48.7 | 46.0 | 319.3 | Yes | Chronic | NA | NA | NA | fALFF | MRI 3.0 | SPM8 | p<0.05 corrected | 8.5 |
|  | 41 | 40 | 46.0 | 46.0 | 282.1 | Yes | Chronic | NA | NA | NA | fALFF | MRI 3.0 | SPM8 | p<0.05 corrected | 8.5 |
| Zhu2015^148^ | 100 | 94 | 33.6 | 33.3 | 122.9 | Yes | Chronic | 71.3 | 17.0 | 20.1 | ASL | MRI 3.0 | SPM8 | p<0.05 FWE corrected | 9.0 |
| Zhu2016^149#^ | 19 | 30 | 34.6 | 35.3 | 105.8 | Yes | Chronic | 86.7 | 25.5 | 21.4 | ASL | MRI 3.0 | SPM8 | p<0.05 Bonferroni corrected | 9.5 |
|  | 30 | 30 | 35.9 | 35.3 | 151.6 | Yes | Chronic | 54.7 | 9.9 | 17.3 | ASL | MRI 3.0 | SPM8 | p<0.05 Bonferroni corrected | 9.5 |
| Zou2019^150^ | 46 | 50 | 23.4 | 23.4 | 4.3 | No | FE | 85.2 | 17.0 | 36.6 | fALFF | MRI 3.0 | SPM12 | p<0.05 FDR corrected | 10.0 |

*Abbreviations:* SCZ, schizophrenia; HCs, healthy controls; PANSS, The positive and negative syndrome scale; NA, not available; fMRI, functional magnetic resonance imaging; ALFF, amplitude of low-frequency fluctuation; fALFF, the fractional amplitude of low-frequency fluctuation; ReHo, Regional Homogeneity; PET, Positron Emission Computed Tomography; SPECT, Single-Photon Emission Computed Tomography; ASL, Arterial Spin Labeling; SPM, statistical parametric mapping; FWE, family wise error; FDR, false discovery rate; GRF, gaussian random field; FSL, FMRIB’s Software Library, the University of Oxford; DPARSF, data processing assistant for resting-State fMRI software; TFCE, the Threshold-Free Cluster Enhancement; REST, the resting-state fMRI data analysis toolkit.

Note: Unless otherwise indicated, data are means.

**Table S5. Demographic, clinical and imaging characteristics of the included studies of GMV for FDRs-SCZ**

| **Study** | **Demographic characteristics** | | | | **Imaging characteristics** | | | | **Quality**  **score^** |
| --- | --- | --- | --- | --- | --- | --- | --- | --- | --- |
|  | **Participants, n** | | **Mean age, year** | | **Imaging technique*** | **Scanner** | **Software** | **Threshold** |  |
|  | **FDRs-SCZ** | **HCs** | **FDRs-SCZ** | **HCs** |  |  |  |  |  |
| Boos2011^151^ | 186 | 122 | 27.50 | 27.50 | VBM | MRI 1.5 | SPM | p<0.01 FDR corrected | 10.0 |
| Borgwardt2010^152^ | 28 | 34 | 37.70 | 39.30 | VBM | MRI 1.5 | SPM2 | p<0.05 corrected | 9.5 |
| Guo2014^153^ | 45 | 43 | 23.11 | 23.67 | VBM | MRI 3.0 | SPM8 | P<0.001 uncorrected | 9.5 |
| Guo2015^12^ | 46 | 46 | 22.96 | 23.30 | VBM | MRI 3.0 | SPM8 | P<0.005 GRF corrected | 10.0 |
| Honea2008^154^ | 213 | 212 | 36.50 | 33.31 | VBM | MRI 1.5 | SPM2 | P<0.05 FWE corrected | 10.0 |
| Hu2013^155^ | 48 | 62 | 22.60 | 23.20 | VBM | MRI 3.0 | SPM8 | P<0.05 GRF corrected | 10.0 |
| Huang2015^156^ | 18 | 18 | 25.56 | 25.06 | VBM | MRI 3.0 | SPM8 | P<0.001 uncorrected | 8.5 |
| Huang2016^157^ | 26 | 27 | 25.56 | 27.44 | VBM | MRI 1.5 | SPM8 | P<0.001 uncorrected | 9.5 |
| Job2003^158^ | 146 | 36 | 21.18 | 21.17 | VBM | SPE 1.0 | SPM99 | p<0.05 corrected | 10.0 |
| Knochel2012^159^ | 29 | 37 | 40.34 | 39.36 | VBM | MRI 3.0 | SPM8 | p<0.05 uncorrected | 9.0 |
| Lei2015^160^ | 67 | 84 | 43.50 | 32.54 | VBM | MRI 3.0 | SPM8 | P<0.05 FWE corrected | 10.0 |
| Li2015^161^ | 10 | 10 | 22.70 | 24.10 | VBM | MRI 3.0 | SPM5 | P<0.001 uncorrected | 9.0 |
| Lin2022^162^ | 18 | 54 | 23.9 | 25.9 | VBM | MRI 3.0 | SPM12 | P<0.05 FWE corrected | 10.0 |
| Lui2009^163^ | 20 | 20 | 43.5 | 33.1 | VBM | MRI 3.0 | SPM2 | p<0.05 corrected | 10.0 |
| McIntosh2004^164^ | 72 | 49 | 35.92 | 35.27 | VBM | MRI 1.5 | SPM99 | p<0.01 corrected | 10.0 |
| Tian2011^165^ | 55 | 59 | 50.30 | 37.28 | VBM | MRI 3.0 | SPM5 | P<0.05 AlphaSim corrected | 10.0 |
| Vander2014^166^ | 89 | 69 | 32.10 | 33.50 | VBM | MRI 3.0 | SPM | P<0.05 FWE corrected | 10.0 |
| Wagshal2015^167^ | 14 | 46 | 12.07 | 12.87 | VBM | MRI 3.0 | FSL | P<0.05 FWE corrected | 9.5 |
| Wang2005^20^ | 26 | 39 | 36.46 | 34.05 | VBM | MRI 1.5 | SPM2 | P<0.001 uncorrected | 9.5 |

*Abbreviations:* FDRs-SCZ, first-degree relatives of probands with schizophrenia; HCs, healthy controls; NA, not available; GMV, gray matter volume; SPM, statistical parametric mapping; FWE, family wise error; FDR, false discovery rate; GRF, gaussian random field; FSL, FMRIB’s Software Library, the University of Oxford; TFCE, the Threshold-Free Cluster Enhancement; REST, the resting-state fMRI data analysis toolkit.

**Table S6. Demographic, clinical and imaging characteristics of the included studies of GMV for SCZ**

| **Study** | **Demographic characteristics** | | | | **Clinical characteristics (SCZ patients)** | | | | | | **Imaging characteristics** | | | | **Quality**  **score^** |
| --- | --- | --- | --- | --- | --- | --- | --- | --- | --- | --- | --- | --- | --- | --- | --- |
|  | **Participants,n** | | **Mean age, year** | | **Illness Duration**  **(months)** | **Medication** | **FE**  **/Chronic** | **PANSS**  **total**  **score** | **PANSS**  **positive**  **score** | **PANSS**  **negative score** | **Imaging technique** | **Scanner** | **Software** | **Threshold** |  |
|  | **SCZ** | **HCs** | **SCZ** | **HCs** |  |  |  |  |  |  |  |  |  |  |  |
| Amann2016^168^ | 45 | 45 | 43.2 | 43.3 | 258.0 | Yes | Chronic | NA | 13.3 | 17.8 | VBM | MRI 1.5T | FSL | p<0.01 FWE corrected | 9.5 |
| Ananth2002^169^ | 20 | 20 | 37.8 | 38.6 | 184.2 | Yes | Chronic | NA | NA | NA | VBM | MRI 2.0T | SPM99 | p<0.05 FWE corrected | 8.5 |
| Asami2012^170^ | 33 | 36 | 22.5 | 22.9 | 4.9 | Yes | FE | NA | NA | NA | VBM | MRI 1.5T | SPM5 | p<0.01 FDR corrected | 8.5 |
| Barsic2020^171^ | 61 | 63 | 30.3 | 33.0 | 60.0 | NA | Chronic | NA | NA | NA | VBM | MRI 1.5T | SPM8 | p<0.05 FWE corrected | 9.0 |
|  | 45 | 63 | 43.2 | 33.0 | 204.0 | NA | Chronic | NA | NA | NA | VBM | MRI 1.5T | SPM8 | p<0.05 FWE corrected | 9.0 |
| Barsic2021^172^ | 57 | 50 | 44.6 | 35.8 | 165.5 | NA | Chronic | 106.0 | 26.1 | 28.6 | VBM | MRI 1.5T | SPM12 | p<0.05 FDR corrected | 9.5 |
|  | 43 | 50 | 29.4 | 35.8 | 116.6 | NA | Chronic | 105.8 | 27.6 | 27.5 | VBM | MRI 1.5T | SPM12 | p<0.05 FDR corrected | 9.5 |
| Bassitt2007^173^ | 50 | 30 | 31.7 | 31.2 | 136.8 | Yes | Chronic | 59.1 | 12.9 | 19.8 | VBM | MRI 1.5T | SPM2 | p<0.05 FWE corrected | 9.5 |
| Berge2011^174^ | 21 | 20 | 24.8 | 25.3 | NA | No | FE | 84.4 | 26.2 | 17.5 | VBM | NA | SPM5 | p<0.01 uncorrected | 9.0 |
| Bonilha2008^175^ | 14 | 13 | 40.0 | 35.0 | NA | No | NA | 96.2 | 24.2 | 21.3 | VBM | MRI 3.0T | SPM5 | NA | 9.0 |
| Bose2009^176^ | 34 | 33 | 39.5 | 39.5 | 144.0 | NA | Chronic | NA | NA | NA | VBM | MRI 1.0T | SPM99 | NA | 8.5 |
| Brown2011^177^ | 17 | 21 | 44.8 | 45.0 | 229.2 | Yes | Chronic | NA | 12.3 | 13.5 | VBM | MRI 1.5T | SPM5 | p<0.05 corrected | 9.5 |
| Cascella2010^178^ | 50 | 90 | 39.8 | 46.3 | 189.0 | Yes | Chronic | NA | NA | NA | VBM | MRI 1.5T | SPM5 | p<0.05 FDR corrected | 8.5 |
| Chang2016^179^ | 60 | 71 | 18.3 | 20.6 | 8.1 | NA | FE | NA | NA | NA | VBM | MRI 3.0T | SPM8 | p<0.01 AlphaSim corrected | 8.5 |
| Chen2023^180^ | 150 | 119 | 19.1 | 18.8 | NA | No | FE | 85.3 | 20.2 | 22.4 | VBM | MRI 3.0T | SPM12 | p<0.05 GRF corrected | 10.0 |
| Cocchi2009^181^ | 21 | 41 | 21.6 | 22.5 | 1.2 | Yes | FE | NA | NA | NA | VBM | MRI 1.5T | SPM5 | NA | 8.5 |
| Corradi2012^182^ | 68 | 77 | 40.2 | 39.6 | 115.9 | Yes | Chronic | NA | NA | NA | VBM | MRI 1.5T | SPM5 | p<0.01 uncorrected | 8.5 |
| Cui2007^183^ | 20 | 20 | 27.2 | 28.1 | 62.2 | Yes | Chronic | NA | 18.8 | 15.7 | VBM | MRI 3.0T | SPM2 | p<0.001 uncorrected | 9.5 |
| Cui2011^184^ | 23 | 36 | 24.8 | 26.6 | 48.5 | Yes | Chronic | NA | 22.5 | 20.9 | VBM | MRI 3.0T | SPM5 | p<0.001 uncorrected | 9.5 |
| Delvecchio2017^185^ | 61 | 59 | 40.8 | 40.2 | 175.2 | Yes | Chronic | NA | NA | NA | VBM | MRI 1.5T | SPM12 | NA | 8.5 |
| DeRamus2020^186^ | 167 | 159 | 38.2 | NA | NA | NA | NA | NA | 14.0 | 14.0 | VBM | MRI 3.0T | SPM12 | p<0.05 FDR corrected | 8.5 |
| Egashira2014^187^ | 24 | 41 | 58.2 | 58.8 | 415.2 | Yes | Chronic | NA | 11.8 | 19.0 | VBM | MRI 1.5T | SPM8 | p<0.05 FWE corrected | 9.5 |
|  | 22 | 41 | 58.3 | 58.8 | 62.4 | Yes | Chronic | NA | 8.2 | 8.4 | VBM | MRI 1.5T | SPM8 | p<0.05 FWE corrected | 9.5 |
| Euler2009^188^ | 19 | 20 | 43.3 | 43.3 | NA | Yes | NA | NA | NA | NA | VBM | MRI 1.5T | SPM5 | NA | 8.5 |
| Fan2022^189^ | 30 | 39 | 12.3 | NA | NA | No | FE | NA | NA | NA | VBM | MRI 3.0T | SPM12 | p<0.05 GRF corrected | 10.0 |
|  | 64 | 61 | 15.7 | NA | NA | No | FE | NA | NA | NA | VBM | MRI 3.0T | SPM12 | p<0.05 GRF corrected | 10.0 |
| Fan2022a^190^ | 28 | 48 | 26.5 | 29.1 | NA | No | FE | 95.7 | NA | NA | VBM | MRI 3.0T | SPM12 | p<0.005 uncorrected | 10.0 |
|  | 28 | 48 | 26.1 | 29.1 | NA | No | FE | 74.9 | NA | NA | VBM | MRI 3.0T | SPM12 | p<0.005 uncorrected | 10.0 |
| Feng2022^49^ | 34 | 34 | 31.0 | 28.3 | NA | No | FE | 85.7 | 23.5 | 19.9 | VBM | MRI 3.0T | SPM12 | p<0.05 uncorrected | 10.0 |
| Filippi2014^191^ | 43 | 17 | 29.3 | 30.7 | 7.9 | No | FE | 100.8 | 28.2 | 23.0 | VBM | MRI 1.5T | NA | p<0.001 uncorrected | 9.5 |
| Fukuta2013^192^ | 40 | 50 | 45.6 | 45.0 | 276.0 | Yes | NA | NA | NA | NA | VBM | MRI 1.5T | SPM5 | p<0.05 FWE corrected | 8.5 |
| Giuliani2015^193^ | 41 | 34 | 39.0 | 34.7 | 207.6 | NA | NA | NA | NA | NA | VBM | MRI 1.5T | SPM2 | p<0.005 FDR corrected | 8.5 |
| Guo2014a^194^ | 19 | 19 | 24.0 | 25.2 | 9.4 | Yes | FE | 77.8 | 19.5 | 20.3 | VBM | MRI 3.0T | SPM8 | p<0.005 uncorrected | 10.0 |
| Guo2014b^153^ | 20 | 43 | 23.4 | 23.7 | 12.9 | No | FE | 88.9 | 22.8 | 22.2 | VBM | MRI 3.0T | SPM8 | p<0.001 uncorrected | 9.5 |
| Guo2014c^195^ | 51 | 41 | 22.5 | 22.8 | 8.4 | No | FE | 91.3 | 22.4 | 22.8 | VBM | MRI 3.0T | SPM8 | p<0.05 FDR corrected | 9.5 |
| Guo2015a^196^ | 49 | 50 | 22.69 | 23.48 | 22.5 | No | FE | 91.31 | 22.27 | 22.82 | VBM | MRI 3.0T | SPM8 | p<0.005 GRF corrected | 9.5 |
| Guo2019^197^ | 33 | 33 | 24.3 | 23.8 | NA | Yes | FE | 88.1 | 22.4 | 20.8 | VBM | MRI 3.0T | FSL | p<0.05 FWE corrected | 9.5 |
| Herold2009^198^ | 18 | 21 | 28.7 | 27.4 | 40.8 | Yes | Chronic | 68.3 | 14.2 | 19.6 | VBM | MRI 1.0T | SPM2 | p<0.005 uncorrected | 10.0 |
| Hirao2008^199^ | 20 | 20 | 36.7 | 35.0 | 127.2 | Yes | Chronic | NA | 14.6 | 16.2 | VBM | MRI 3.0T | SPM2 | p<0.05 FDR corrected | 9.5 |
| Horn2010^200^ | 20 | 20 | 31.1 | NA | NA | Yes | NA | NA | NA | NA | VBM | MRI 1.5T | SPM5 | p<0.05 FDR corrected | 8.5 |
| Hu2013^155^ | 51 | 59 | 22.3 | 23.2 | 11.1 | No | FE | 91.5 | 22.4 | 22.8 | VBM | MRI 3.0T | SPM8 | p<0.05 FDR corrected | 9.5 |
| Huang2009^201^ | 15 | 38 | 31.0 | 35.3 | NA | No | FE | NA | NA | NA | VBM | MRI 3.0T | SPM5 | NA | 8.5 |
| Huang2015^202^ | 18 | 18 | 22.6 | 25.1 | 5.9 | No | FE | 106.4 | 31.1 | 25.8 | VBM | MRI 3.0T | SPM8 | p<0.001 uncorrected | 9.5 |
|  | 18 | 18 | 22.7 | 25.1 | 12.4 | No | FE | 88.1 | 18.6 | 22.1 | VBM | MRI 3.0T | SPM8 | p<0.001 uncorrected | 9.5 |
| Hyza2014^203^ | 24 | 24 | 32.8 | 31.8 | 110.4 | Yes | Chronic | NA | NA | NA | VBM | MRI 1.5T | SPM8 | p<0.05 FWE corrected | 8.5 |
| Jayakumar2005^204^ | 18 | 18 | 24.9 | 25.7 | 10.3 | No | FE | 79.0 | 19.0 | 23.0 | VBM | MRI 1.5T | SPM2 | p<0.05 FDR corrected | 10.0 |
| Jin2020^205^ | 26 | 22 | 27.5 | 33.2 | 41.7 | NA | NA | 82.8 | NA | NA | VBM | MRI 3.0T | SPM12 | p<0.05 FDR corrected | 8.5 |
| Kang2022^206^ | 52 | 51 | 26.0 | 26.0 | NA | No | FE | NA | NA | NA | VBM | MRI 3.0T | SPM12 | p<0.05 GRF corrected | 8.5 |
| Kaspareka2007^207^ | 22 | 18 | 23.7 | 24.1 | 9.6 | No | FE | 51.2 | 9.1 | 15.4 | VBM | MRI 1.5T | SPM2 | p<0.001 uncorrected | 10.0 |
| Kawada2009^208^ | 26 | 26 | 36.7 | 36.3 | 118.8 | Yes | Chronic | 61.1 | 14.5 | 15.5 | VBM | MRI 3.0T | SPM5 | p<0.05 FDR corrected | 10.0 |
| Kim2017^209^ | 22 | 22 | 31.7 | 31.6 | 110.4 | Yes | Chronic | NA | 18.4 | 21.1 | VBM | MRI 3.0T | SPM8 | p<0.05 FWE corrected | 9.5 |
| Koelkebeck2013^210^ | 18 | 30 | 34.9 | 39.1 | 141.6 | Yes | Chronic | NA | 12.8 | 15.1 | VBM | MRI 3.0T | SPM8 | p<0.001 uncorrected | 9.5 |
| Kong2015^211^ | 22 | 20 | 54.0 | 52.8 | 378.5 | Yes | Chronic | NA | NA | NA | VBM | MRI 3.0T | SPM8 | p<0.05 FDR corrected | 8.5 |
| Kuhn2012^212^ | 29 | 45 | 27.6 | 31.3 | 45.6 | NA | NA | NA | NA | NA | VBM | MRI 3.0T | SPM5 | NA | 8.5 |
| Lee2020^213^ | 65 | 65 | 37.0 | 34.5 | 186.2 | NA | NA | NA | NA | NA | VBM | MRI 3.0T | SPM8 | p<0.017 FWE corrected | 8.5 |
| Lei2015^160^ | 88 | 44 | 23.0 | 22.6 | NA | No | FE | 92.4 | 23.3 | 22.1 | VBM | MRI 3.0T | SPM8 | p< 0.05 FWE corrected | 9.5 |
| Li2019^214^ | 86 | 86 | 23.5 | 24.0 | 14.2 | NA | FE | 86.8 | 21.7 | 20.6 | VBM | MRI 3.0T | FSL、SPM8 | p<0.05 FWE corrected | 9.5 |
| Li2022^215^ | 44 | 44 | 23.1 | 22.6 | 7.0 | No | FE | 92.2 | 23.7 | 22.1 | VBM | MRI 3.0T | SPM8 | p< 0.05 FWE corrected | 9.5 |
| Li2023^216^ | 343 | 342 | 23.8 | 24.3 | NA | No | FE | NA | NA | NA | VBM | MRI 3.0T | SPM8 | p<0.05 FDR corrected | 10.0 |
| Liao2015^217^ | 93 | 99 | 27.0 | 25.8 | 54.1 | Yes | NA | 77.3 | 23.4 | 18.3 | VBM | MRI 3.0T | SPM8 | p< 0.05 FWE corrected | 9.5 |
| Lin2022^162^ | 26 | 54 | 24.0 | 25.9 | 24.8 | Yes | FE | 90.9 | 24.9 | 23.8 | VBM | MRI 3.0T | SPM12 | p< 0.05 FWE corrected | 10.0 |
| Liu2020^218^ | 27 | 28 | 27.7 | 26.1 | 74.8 | Yes | NA | 83.8 | 21.3 | 19.9 | VBM | MRI 3.0T | SPM8 | p<0.05 FWE corrected | 9.5 |
|  | 48 | 28 | 29.0 | 26.1 | 86.0 | Yes | NA | 84.7 | 25.0 | 17.1 | VBM | MRI 3.0T | SPM8 | p<0.05 FWE corrected | 9.5 |
| Liu2022^219^ | 64 | 32 | 30.2 | 28.8 | 90.0 | Yes | Chronic | 90.0 | 23.0 | 25.1 | VBM | MRI | SPM12 | p<0.05 FDR corrected | 9.5 |
|  | 70 | 95 | 28.4 | 30.2 | 43.3 | Yes | Chronic | 78.5 | 22.7 | 19.9 | VBM | MRI | SPM12 | p<0.05 FDR corrected | 9.5 |
| Lui2009^220^ | 68 | 68 | 24.2 | 24.7 | 8.6 | No | FE | 107.4 | 26.6 | 20.6 | VBM | MRI 3.0T | SPM2 | p<0.05 FWE corrected | 10.0 |
| Lv2007^221^ | 13 | 13 | 24.0 | NA | NA | No | FE | NA | NA | NA | VBM | MRI 3.0T | SPM2 | p<0.05 corrected | 8.5 |
| Lyu2014^222^ | 51 | 59 | 22.3 | 23.2 | 11.1 | No | FE | 91.5 | 22.4 | 22.8 | VBM | MRI 3.0T | SPM8 | p<0.005 FDR corrected | 9.5 |
| Ma2021^223^ | 64 | 65 | 26.7 | 25.3 | NA | Yes | NA | NA | NA | NA | VBM | MRI 3.0T | SPM | p< 0.05 FWE corrected | 8.5 |
| Madeira2020^224^ | 20 | 20 | 31.5 | 31.5 | 72.0 | Yes | NA | NA | NA | NA | VBM | MRI 3.0T | SPM12 | p<0.05 FWE corrected | 8.5 |
| Maggioni2017^225^ | 243 | 383 | 33.2 | 30.4 | NA | Yes | NA | NA | NA | NA | VBM | MRI 3.0T | SPM12 | p<0.05 FWE corrected | 8.5 |
| Marti-Bonmati2007^226^ | 21 | 10 | 39.0 | 35.0 | 180.0 | Yes | Chronic | NA | NA | NA | VBM | MRI 1.5T | SPM2 | p<0.005 uncorrected | 8.5 |
| Martin2014^227^ | 17 | 50 | 46.1 | 46.5 | NA | Yes | Chronic | NA | 27.5 | 18.2 | VBM | MRI 3.0T | SPM8 | p<0.05 FWE corrected | 8.5 |
|  | 65 | 50 | 44.6 | 46.5 | NA | Yes | NA | NA | 27.6 | 16.7 | VBM | MRI 3.0T | SPM8 | p<0.05 FWE corrected | 8.5 |
| Mcdonald2005^228^ | 25 | 52 | 37.3 | 39.3 | NA | NA | NA | NA | NA | NA | VBM | MRI 1.5T | SPM99 | NA | 8.5 |
| Molina2010^229^ | 30 | 40 | 25.8 | 29.4 | 14.5 | No | FE | 41.5 | 21.9 | 19.2 | VBM | MRI 1.5T | SPM5 | p<0.05 FDR corrected | 9.5 |
| Molina2010a^230^ | 26 | 41 | 36.3 | 29.4 | 124.8 | Yes | Chronic | NA | 24.0 | 26.2 | VBM | MRI 1.5T | SPM8 | p<0.05 FWE corrected | 9.5 |
|  | 19 | 41 | 36.9 | 29.4 | 130.8 | Yes | Chronic | NA | 21.2 | 28.3 | VBM | MRI 1.5T | SPM8 | p<0.05 FWE corrected | 9.5 |
| Molina2011a^231^ | 38 | 24 | 34.4 | 34.6 | 117.6 | Yes | Chronic | NA | 23.2 | 27.0 | VBM | MRI 1.5T | SPM8 | p<0.05 FDR corrected | 9.5 |
| Molina2011b^232^ | 30 | 31 | 34.1 | 36.8 | 160.8 | Yes | Chronic | 97.3 | 28.3 | 24.1 | VBM | MRI 1.5T | SPM8 | NA | 9.5 |
| Morgan2007^233^ | 44 | 44 | 27.1 | NA | 16.8 | No | FE | NA | NA | NA | VBM | MRI 1.5T | NA | NA | 8.5 |
| Moriya2010^234^ | 19 | 19 | 29.9 | 29.7 | 9.4 | Yes | FE | 68.2 | 15.9 | 17.2 | VBM | MRI 3.0T | SPM5 | p<0.05 FDR corrected | 9.5 |
| Nagashima2012^235^ | 8 | 8 | 43.8 | 44.8 | 244.6 | Yes | Chronic | NA | 17.5 | 26.1 | VBM | MRI 1.5T | SPM5 | p<0.001 uncorrected | 9.5 |
| Nakamura2013^236^ | 34 | 51 | 24.7 | 23.9 | 1.7 | Yes | FE | NA | NA | NA | VBM | MRI 1.5T | SPM8 | p<0.001 FWE corrected | 8.5 |
| Narayanaswamy2015^237^ | 55 | 45 | 28.6 | 30.3 | 16.3 | No | FE | NA | 21.7 | 23.9 | VBM | MRI 1.5T | SPM8 | p<0.05 FWE corrected | 9.5 |
| Neckelmann2006^238^ | 12 | 12 | NA | NA | NA | Yes | NA | NA | NA | NA | VBM | MRI 1.5T | SPM99 | p<0.001 uncorrected | 8.5 |
| Nenadic2012^239^ | 99 | 113 | 36.2 | 32.4 | NA | Yes | Chronic | NA | NA | NA | VBM | MRI 3.0T | vbm2 | NA | 8.5 |
| Nenadic2015a^240^ | 24 | 49 | 24.9 | 23.8 | 4.1 | No | FE | 53.2 | 30.4 | 29.1 | VBM | MRI 3.0T | SPM8 | p<0.001 uncorrected | 10.0 |
| Nenadic2015b^241^ | 34 | 34 | 33.0 | 34.3 | 106.8 | Yes | Chronic | NA | NA | NA | VBM | MRI 3.0T | SPM8 | NA | 8.5 |
| Neugebauer2019^242^ | 18 | 19 | 36.9 | 35.8 | 151.0 | NA | NA | 84.8 | 15.3 | 23.6 | VBM | MRI 3.0T | SPM12 | p<0.001 uncorrected | 9.0 |
| Odaly2007^243^ | 28 | 32 | 33.0 | 34.0 | 96.0 | NA | NA | NA | NA | NA | VBM | MRI 1.5T | NA | NA | 8.5 |
| Oertel-Knochel2012^159^ | 37 | 31 | 38.0 | 39.4 | 164.5 | Yes | Chronic | 62.8 | 15.7 | 15.1 | VBM | MRI 3.0T | SPM8 | NA | 9.5 |
| Onay2017^244^ | 20 | 16 | 36.5 | 34.4 | 128.4 | Yes | Chronic | NA | NA | NA | VBM | MRI 1.5T | SPM8 | p<0.05 FWE corrected | 8.5 |
| Ota2011^245^ | 9 | 10 | 29.0 | 26.1 | NA | NA | FE | NA | NA | NA | VBM | MRI 1.5T | SPM5 | p<0.005 uncorrected | 8.5 |
| Paillere2001^246^ | 20 | 20 | 29.0 | 26.0 | 120.0 | NA | NA | NA | 17.3 | 27.6 | VBM | MRI 1.5T | SPMs | NA | 8.5 |
| Picado2015^247^ | 20 | 20 | 35.9 | 33.2 | NA | Yes | NA | 59.4 | 12.3 | 15.5 | VBM | MRI 1.5T | SPM5 | p< 0.05 FWE corrected | 9.0 |
| Plaze2009^248^ | 15 | 20 | 31.0 | 31.9 | 104.4 | NA | NA | NA | NA | NA | VBM | MRI 1.5T | SPM2 | p< 0.05 FWE corrected | 8.5 |
|  | 12 | 20 | 35.0 | 31.9 | 169.2 | NA | NA | NA | NA | NA | VBM | MRI 1.5T | SPM2 | p< 0.05 FWE corrected | 8.5 |
| Ren2013^91^ | 100 | 100 | 24.3 | 24.4 | NA | No | FE | 97.9 | 25.1 | 18.8 | VBM | MRI 3.0T | SPM8 | p<0.05 FDR corrected | 9.5 |
| Salgado-pineda2004^249^ | 14 | 14 | 25.1 | 25.1 | 17.5 | Yes | NA | NA | NA | NA | VBM | MRI 1.5T | SPM2 | p<0.05 FDR corrected | 8.5 |
| Salgado-Pineda2014^250^ | 14 | 14 | 37.3 | 34.6 | 168.0 | Yes | Chronic | 60.7 | 24.7 | 13.1 | VBM | MRI 3.0T | SPM5 | p< 0.05 FDR corrected | 9.5 |
| Sapara2016^251^ | 20 | 20 | 37.8 | 35.3 | NA | Yes | NA | 66.8 | 17.1 | 18.2 | VBM | MRI 1.5T | SPM8 | p< 0.05 FWE corrected | 9.0 |
| Schiffer2010^252^ | 24 | 27 | 37.8 | 36.7 | 201.6 | Yes | Chronic | NA | 14.6 | 20.5 | VBM | MRI 1.5T | SPM5 | p< 0.05 FDR corrected | 9.5 |
| Segarra2008^253^ | 28 | 28 | 27.3 | 28.8 | NA | Yes | NA | NA | NA | NA | VBM | MRI 1.5T | SPM2 | p<0.05 FDR corrected | 8.5 |
| Shen2021^254^ | 23 | 22 | 31.2 | 32.4 | 16.1 | NA | FE | 86.3 | NA | NA | VBM | MRI 3.0T | SPM12 | p<0.05 FDR corrected | 9.0 |
|  | 18 | 22 | 33.6 | 32.4 | 59.9 | NA | Chronic | 112.0 | NA | NA | VBM | MRI 3.0T | SPM12 | p<0.05 FDR corrected | 9.0 |
| Shivakumar2018^255^ | 30 | 60 | 32.8 | 26.3 | 32.0 | No | FE | NA | 24.8 | 24.7 | VBM | MRI 3.0T | SPM8 | p<0.05 FWE corrected | 9.5 |
| Singh2014^256^ | 14 | 14 | 34.1 | 32.6 | 115.2 | Yes | Chronic | NA | NA | NA | VBM | MRI 3.0T | SPM8 | p< 0.05 FWE corrected | 8.5 |
| Singh2015^257^ | 14 | 14 | 31.5 | 27.2 | 120.4 | Yes | Chronic | NA | NA | NA | VBM | MRI 3.0T | SPM8 | p< 0.05 FWE corrected | 8.5 |
| Singh2018^258^ | 28 | 28 | 33.9 | 31.4 | 112.9 | Yes | Chronic | NA | NA | NA | VBM | MRI 3.0T | SPM12 | p<0.05 FWE corrected | 8.5 |
| Spalthoff2018^259^ | 51 | 102 | 35.2 | 33.2 | 105.6 | Yes | Chronic | NA | NA | NA | VBM | MRI 3.0T | SPM12 | NA | 8.5 |
| Suazo2014^260^ | 17 | 13 | 33.3 | 30.9 | 63.3 | No | NA | 74.2 | 20.9 | 16.5 | VBM | MRI 1.5T | SPM8 | NA | 9.0 |
| Sun2020^261^ | 46 | 56 | 29.7 | 29.5 | 42.2 | Yes | Chronic | NA | NA | NA | VBM | MRI 3.0T | SPM8 | p<0.05 GRF corrected | 9.0 |
| Szendi2017^262^ | 21 | 13 | 39.0 | 34.0 | 192.0 | Yes | Chronic | 79.3 | 13.8 | 24.3 | VBM | MRI 1.5T | NA | p<0.036 corrected | 9.5 |
| Tomelleri2009^263^ | 70 | 79 | 39.7 | 40.3 | 169.6 | Yes | Chronic | NA | NA | NA | VBM | MRI 1.5T | SPM5 | p<0.05 FWE corrected | 8.5 |
| Torres2016^264^ | 161 | 151 | 30.4 | 30.6 | 60.0 | Yes | NA | 56.1 | NA | NA | VBM | NA | SPM8 | p<0.05 FWE corrected | 8.5 |
| Tregellas2007^265^ | 32 | 32 | 39.6 | 35.3 | NA | Yes | NA | NA | NA | NA | VBM | MRI 1.5T | SPM2 | p<0.05 FDR corrected | 8.5 |
| Tseng2021^266^ | 37 | 26 | 35.7 | 35.6 | 146.3 | Yes | Chronic | 55.8 | 13.4 | 16.7 | VBM | MRI 1.5T | SPM12 | p< 0.001 FWE corrected | 10.0 |
| Van2014^267^ | 51 | 51 | 34.0 | 36.1 | 105.2 | Yes | Chronic | NA | 11.5 | 14.4 | VBM | MRI 3.0T | SPM8 | p<0.005 uncorrected | 9.5 |
| Venkatasubramanian2010^268^ | 30 | 27 | 30.1 | 27.4 | 41.7 | No | FE | NA | 22.0 | 23.0 | VBM | MRI 1.5T | SPM2 | p<0.05 FDR corrected | 9.5 |
| Wei2021^269^ | 31 | 29 | 29.3 | 27.7 | NA | No | FE | 82.0 | 22.9 | 20.3 | VBM | MRI 3.0T | FSL-VBM toolbox | p< 0.05 FWE corrected | 9.5 |
| Whitford2005^270^ | 31 | 30 | 19.3 | 19.3 | 6.4 | Yes | FE | NA | NA | NA | VBM | MRI 1.5T | SPM99 | NA | 8.5 |
| Witthaus2009^271^ | 23 | 29 | 26.4 | 25.7 | NA | NA | FE | NA | 19.3 | 18.0 | VBM | MRI 1.5T | SPM2 | p<0.005 uncorrected | 8.5 |
| Wu2021^272^ | 90 | 90 | 35.4 | 35.3 | 3.1 | No | FE | 85.2 | NA | NA | VBM | MRI 3.0T | FSL | p< 0.05 TFCE corrected | 9.0 |
| Xie2021^118^ | 30 | 33 | 30.3 | 32.0 | 21.4 | Yes | FE | 79.9 | 19.7 | NA | VBM | MRI 3.0T | SPM8 | p<0.05 FDR corrected | 9.5 |
| Yang2015^273^ | 30 | 30 | 21.5 | 24.3 | 6.4 | Yes | FE | 82.4 | NA | NA | VBM | MRI 1.5T | HAMMER | p<0.05 FDR corrected | 9.0 |
| Yang2019^274^ | 37 | 28 | 42.0 | 40.5 | 220.9 | Yes | Chronic | NA | 10.1 | 15.3 | VBM | MRI 3.0T | SPM12 | p< 0.05 FDR corrected | 9.5 |
| Yang2022^275^ | 70 | 95 | 28.4 | 30.2 | 43.3 | Yes | Chronic | 78.5 | 22.7 | 19.9 | VBM | MRI 3.0T | SPM12 | p< 0.01 AlphaSim corrected | 10.0 |
| Yin2021^131^ | 30 | 30 | 19.8 | 20.8 | 8.5 | Yes | FE | 78.3 | 21.8 | 18.8 | VBM | MRI 3.0 | VBM8 | p<0.001 uncorrected | 10.0 |
| Yokoyama2018^276^ | 60 | 40 | 38.0 | 35.1 | 152.3 | Yes | Chronic | NA | 13.8 | 15.9 | VBM | MRI 3.0T | SPM8 | p< 0.05 FDR corrected | 9.5 |
| Yue2016^277^ | 20 | 24 | 24.5 | 24.8 | 22.9 | Yes | FE | 89.0 | 21.3 | 19.1 | VBM | MRI 3.0T | SPM8 | p< 0.05 FWE corrected | 10.0 |
| Zhang2017^278^ | 49 | 57 | 32.9 | 32.9 | 4.9 | No | FE | 89.2 | 25.9 | 16.7 | VBM | MRI 3.0T | SPM8 | NA | 9.5 |
| Zhang2022^279^ | 44 | 48 | 24.7 | 24.8 | NA | No | FE | 75.3 | 20.8 | 16.5 | VBM | MRI 3.0T | SPM12 | p<0.05 FDR corrected | 9.5 |
| Zhao2022^280^ | 52 | 29 | 23.4 | 22.1 | 10.9 | No | FE | 101.5 | 15.2 | 24.7 | VBM | MRI 3.0T | SPM8 | p< 0.005 TFCE corrected | 10.0 |
| Zierhut2013^281^ | 34 | 36 | 34.6 | 31.0 | 120.4 | Yes | Chronic | 71.1 | 16.4 | 19.0 | VBM | MRI 3.0T | SPM5 | p<0.05 FDR corrected | 10.0 |
| Zou2018^282^ | 38 | 40 | 28.9 | 28.2 | NA | No | FE | 40.4 | 25.2 | 21.6 | VBM | MRI 3.0T | SPM8 | p<0.001 uncorrected | 9.5 |

*Abbreviations:* GMV, Gray matter volume; VBM, voxel-based morphometry; SCZ, schizophrenia; HCs, healthy controls; NA, not available; PANSS, The positive and negative syndrome scale; SPM, statistical parametric mapping; FWE, family wise error; GRF, gaussian random field; FDR, false discovery rate; TFCE, the Threshold-Free Cluster Enhancement; FSL, FMRIB’s Software Library, the University of Oxford.

Note: Unless otherwise indicated, data are means.

**Table S7. GMV differences between SCZ and HCs**

|  | **Local Maximum** |  |  |  | **Cluster** |  |
| --- | --- | --- | --- | --- | --- | --- |
| **Comparison** | **Peak region** | **MNI coordinate**  **(x, y, z)** | **SDM-Z value** | ***p* value** | **No. of voxels** | **Breakdown (No. of voxels)** |
| ***SCZ* < *HCs*** | Left inferior frontal gyrus, BA 45 | -46, 16, 4 | -9.476 | <0.001 | 7100 | Left insula, BA 48 (1978)  Left superior temporal gyrus, BA 48 (1815)  Left inferior frontal gyrus, triangular part, BA 45 (1290)  Left middle temporal gyrus, BA 22 (324)  Left amygdala, BA 34 (53)  Left lenticular nucleus, putamen, BA 48 (21) |
|  | Right inferior frontal gyrus, BA 48 | 48, 10, 4 | -8.234 | <0.001 | 5953 | Right insula, BA 48 (1624)  Right superior temporal gyrus, BA 22 (1612)  Right middle temporal gyrus, BA 21 (991)  Right inferior frontal gyrus, opercular part, BA 48 (507)  Right lenticular nucleus, putamen, BA 48 (29)  Right amygdala, BA 36 (4) |
|  | Left superior frontal gyrus, medial, BA 10 | -6, 44, -8 | -6.367 | <0.001 | 3638 | Left anterior cingulate / paracingulate gyri, BA 32 (776)  Left superior frontal gyrus, medial, BA 32 (1146)  Right superior frontal gyrus, medial orbital, BA 11 (637)  Right anterior cingulate / paracingulate gyri, BA 32 (417)  Right median cingulate / paracingulate gyri, BA 32 (159)  Left median cingulate / paracingulate gyri, BA 24 (55) |
|  | Right median cingulate / paracingulate gyri, BA 23 | 2, -30, 36 | -6.246 | 0.002 | 1162 | Right median cingulate / paracingulate gyri (536)  Left median cingulate / paracingulate gyri (497) |
|  | Left fusiform gyrus, BA 37 | -26, -50, -16 | -4.562 | 0.029 | 70 | Left fusiform gyrus, BA 37 (56) |

*Abbreviations*: GMV, grey matter volume; SCZ, schizophrenia; HCs, healthy controls; MNI, Montreal Neurological Institute; SDM, the Seed-based d Mapping.

**Table S8. Overlapping of resting-state functional activity differences and GMV differences in SCZ**

| **Local Maximum** | **Cluster** |  |
| --- | --- | --- |
| **Region** | **No. of voxels** | **Breakdown (No. of voxels)** |
| ***Functional activity increase & GMV decrease (SCZ vs HCs)*** | | |
| Left lenticular nucleus, putamen | 20 | Left lenticular nucleus, putamen (20) |
| ***Functional activity decrease & GMV decrease (SCZ vs HCs)*** | | |
| Left anterior cingulate / paracingulate gyri | 953 | Left anterior cingulate / paracingulate gyri (394)  Right superior frontal gyrus, medial orbital (258)  Right anterior cingulate / paracingulate gyri (182)  Left superior frontal gyrus, medial orbital (137)  Right median cingulate / paracingulate gyri, BA 24 (23)  Left median cingulate / paracingulate gyri, BA 24 (13) |

***Abbreviations*:** SCZ, schizophrenia; HCs, healthy controls; MNI, Montreal Neurological Institute.

**Table S9. Resting-state functional brain activity differences between medical patients with SCZ and HCs**

|  | **Local Maximum** |  |  |  | **Cluster** |  |
| --- | --- | --- | --- | --- | --- | --- |
| **Comparison** | **Peak region** | **MNI coordinate**  **(x, y, z)** | **SDM-Z value** | ***p* value** | **No. of voxels** | **Breakdown (No. of voxels)** |
| **Resting-state functional brain activity** | | | | | | |
| ***SCZ >*** ***HCs*** | Left striatum | -26, -4, -8 | 5.546 | 0.008 | 169 | Left striatum (59)  Left lenticular nucleus, putamen, BA 48 (31)  Left caudate nucleus, BA 25 (4) |
| ***SCZ < HCs*** | Left anterior cingulate / paracingulate gyri, BA 10 | -2, 50, 6 | -6.226 | <0.001 | 1433 | Left anterior cingulate / paracingulate gyri, BA 10 (530) Right anterior cingulate / paracingulate gyri, BA 32 (264)  Left superior frontal gyrus, medial, BA 10 (254)  Right superior frontal gyrus, medial, BA 10 (86) |
|  | Right postcentral gyrus, BA 4 | 48, -14, 38 | -6.195 | <0.001 | 542 | Right postcentral gyrus, BA 4 (248)  Right precentral gyrus, BA 6 (235) |

*Abbreviations*: GMV, grey matter volume; SCZ, schizophrenia; HCs, healthy controls; MNI, Montreal Neurological Institute; SDM, the Seed-based *d* Mapping; BA, Brodmann area.

**Table S10. Resting-state functional brain activity differences between chronic patients with SCZ and HCs**

|  | **Local Maximum** |  |  |  | **Cluster** |  |
| --- | --- | --- | --- | --- | --- | --- |
| **Comparison** | **Peak region** | **MNI coordinate**  **(x, y, z)** | **SDM-Z value** | ***p* value** | **No. of voxels** | **Breakdown (No. of voxels)** |
| **Resting-state functional brain activity** | | | | | | |
| ***SCZ >*** ***HCs*** | Left striatum | -20, 2, -10 | 7.274 | <0.001 | 483 | Left striatum (168)  Left lenticular nucleus, putamen, BA 48 (86)  Left caudate nucleus, BA 25 (6) |
|  | Right inferior frontal gyrus, BA 47 | 38, 38, -12 | 6.522 | 0.016 | 89 | Right inferior frontal gyrus, BA 47 (75) |
| ***SCZ < HCs*** | Right postcentral gyrus, BA 6 | 50, -30, 46 | -6.845 | <0.001 | 368 | Right postcentral gyrus, BA 4 (173)  Right precentral gyrus, BA 6 (149) |
|  | Left anterior cingulate / paracingulate gyri, BA 10 | -2, 44, 4 | -5.609 | 0.009 | 190 | Left anterior cingulate / paracingulate gyri, BA 10 (99) Right anterior cingulate / paracingulate gyri, BA 32 (46)  Right superior frontal gyrus, medial, BA 10 (31)  Left superior frontal gyrus, medial, BA 10 (12) |
|  | Right middle temporal gyrus, BA 37 | 48, -68, -2 | -6.177 | 0.007 | 118 | Right middle temporal gyrus, BA 37 (73) |

*Abbreviations*: GMV, grey matter volume; SCZ, schizophrenia; HCs, healthy controls; MNI, Montreal Neurological Institute; SDM, the Seed-based *d* Mapping; BA, Brodmann area.

**Table S11. Resting-state functional brain activity differences between FDRs-SCZ and HCs in different imaging methodology**

|  | **Local Maximum** |  |  |  | **Cluster** |  |
| --- | --- | --- | --- | --- | --- | --- |
| **Comparison** | **Peak region** | **MNI coordinate**  **(x, y, z)** | **SDM-Z value** | ***p* value** | **No. of voxels** | **Breakdown (No. of voxels)** |
| **ALFF differences** | | | | | | |
| ***FDRs-SCZ > HCs*** | Left anterior cingulate / paracingulate gyri, BA 32 | 2, 40, 22 | 5.002 | <0.001 | 775 | Left anterior cingulate / paracingulate gyri, BA 32 (239)  Left superior frontal gyrus, medial, BA 32 (234)  Right anterior cingulate / paracingulate gyri, BA 32 (146)  Right median cingulate / paracingulate gyri, BA 32 (91)  Left median cingulate / paracingulate gyri, BA 24 (43)  Right superior frontal gyrus, medial, BA 32 (22) |

*Abbreviations*: FDRs-SCZ, first-degree relatives of probands with schizophrenia; HCs, healthy controls; ALFF, amplitude of low-frequency signal fluctuations; MNI, Montreal Neurological Institute; SDM, seed-based *d* mapping.

**Table S12. Resting-state functional brain activity differences between SCZ and HCs in different imaging methodology**

|  | **Local Maximum** |  |  |  | **Cluster** |  |
| --- | --- | --- | --- | --- | --- | --- |
| **Comparison** | **Peak region** | **MNI coordinate**  **(x, y, z)** | **SDM-Z value** | ***p* value** | **No. of voxels** | **Breakdown (No. of voxels)** |
| **ALFF differences** | | | | | | |
| ***SCZ > HCs*** | Right inferior frontal gyrus, BA 47 | 48, 40, -6 | 5.359 | <0.001 | 633 | Right inferior frontal gyrus (509) |
|  | Left inferior temporal gyrus, BA 20 | -50, -8, -38 | 3.675 | <0.001 | 73 | Left inferior temporal gyrus, BA 20 (68) |
|  | Left striatum | -8, 10, -2 | 2.699 | 0.003 | 21 | Left striatum (32) |
| ***SCZ < HCs*** | Right postcentral gyrus, BA 4 | 50, -16, 42 | -5.339 | <0.001 | 897 | Right precentral gyrus (411)  Right postcentral gyrus (368) |
|  | Left postcentral gyrus, BA 3 | -54, -18, 40 | -5.284 | <0.001 | 530 | Left postcentral gyrus (389)  Left precentral gyrus (32) |
| **fALFF differences** | | | | | | |
| ***SCZ > HCs*** | Left striatum | -8, 10, -1 | 3.863 | <0.001 | 126 | Left striatum (84) |
|  | Left angular gyrus, BA 39 | -50, -58, 36 | 3.807 | <0.001 | 106 | Left angular gyrus, BA 39 (60) |
| ***SCZ < HCs*** | Right anterior cingulate / paracingulate gyri, BA 24 | 6, 26, 28 | -4.179 | <0.001 | 1143 | Left anterior cingulate / paracingulate gyri, BA 32 (392)  Right anterior cingulate / paracingulate gyri, BA 32 (231)  Right median cingulate / paracingulate gyri, BA 32 (228)  Left median cingulate / paracingulate gyri, BA 24 (197)  Left superior frontal gyrus, medial, BA 32 (143)  Right superior frontal gyrus, medial, BA 32 (39) |
|  | Left calcarine fissure, BA 17 | -2, -74, 14 | -3.232 | <0.001 | 151 | Left calcarine fissure, BA 17 (118) |
|  | Left calcarine fissure, BA 18 | -12, -94, -8 | -3.230 | <0.001 | 117 | Left calcarine fissure, BA 18 (87) |
| **ReHo differences** | | | | | | |
| ***SCZ > HCs*** | Left striatum | -12, 10, -16 | 5.812 | <0.001 | 423 | Left striatum (311) |
|  | Left superior frontal gyrus, BA 9 | 0, 44, 38 | 3.405 | <0.001 | 131 | Left superior frontal gyrus (120) |
| ***SCZ < HCs*** | Left postcentral gyrus, BA 3 | -50, -16, 38 | -4.246 | <0.001 | 564 | Left postcentral gyrus (443)  Left precentral gyrus (54) |
|  | Left superior frontal gyrus, medial, BA 11 | -2, 46, -14 | -4.627 | <0.001 | 354 | Left superior frontal gyrus, medial, BA 32 (137)  Right superior frontal gyrus, medial, BA 32 (77)  Left anterior cingulate / paracingulate gyri (6)  Right anterior cingulate / paracingulate gyri, (1) |
|  | Right inferior temporal gyrus, BA 37 | 50, -64, -8 | -3.229 | <0.001 | 225 | Right inferior temporal gyrus (120)  Right middle temporal gyrus (104) |
|  | Right postcentral gyrus, BA 4 | 56, -4, 32 | -3.189 | <0.001 | 134 | Right postcentral gyrus (109)  Right precentral gyrus (24) |
|  | Right precuneus | 2, -38, 58 | -3.571 | <0.001 | 110 | Left precuneus (38)  Right precuneus (18) |
| **rCBF differences** | | | | | | |
| ***SCZ > HCs*** | Left striatum | -28, -2, -4 | 5.507 | <0.001 | 355 | Left putamen (168)  Left striatum (103) |
|  | Right striatum | 32, -6, -6 | 4.245 | <0.001 | 352 | Right putamen (249)  Right striatum (79) |
| ***SCZ < HCs*** | Left anterior cingulate / paracingulate gyri, BA 32 | 0, 42, 6 | -6.789 | <0.001 | 3704 | Left anterior cingulate / paracingulate gyri, BA 32 (911)  Left superior frontal gyrus, medial orbital, BA 10 (782)  Right anterior cingulate / paracingulate gyri, BA 32 (724)  Right superior frontal gyrus, medial orbital, BA 10 (584)  Right median cingulate / paracingulate gyri, BA 32 (304)  Left median cingulate / paracingulate gyri, BA 24 (190) |
|  | Left superior frontal gyrus, BA 10 | -34, 56, 0 | -4.653 | <0.001 | 608 | Left middle frontal gyrus (311)  Left superior frontal gyrus (248) |
|  | Right temporal pole, superior temporal gyrus, BA 38 | 54, 10, -12 | -3.165 | <0.001 | 140 | Right temporal pole, superior temporal gyrus (117) |

*Abbreviations*: SCZ, schizophrenia; HCs, healthy controls; ALFF, amplitude of low-frequency signal fluctuations; fALFF, fractional amplitude of low-frequency fluctuations; ReHo, regional homogeneity; rCBF, regional cerebral blood flow; MNI, Montreal Neurological Institute; SDM, seed-based *d* mapping.

**Table S13. Resting-state functional activity alterations in SCZ subgroup (excluded the studies of insufficient number of subjects [n < 10] or uncorrected for statistics)**

| **Local Maximum** |  |  |  | **Cluster** |  |
| --- | --- | --- | --- | --- | --- |
| **Region** | **Peak MNI coordinate**  **(x, y, z)** | **SDM-Z value** | ***p* value** | **No. of voxels** | **Breakdown (No. of voxels)** |
| ***SCZ > HCs*** | | | | | |
| Left striatum | -12, 12, -16 | 6.919 | <0.001 | 637 | Left striatum (232)  Left lenticular nucleus, putamen, BA 48 (176) |
| Right striatum | 26, 2, -6 | 7.015 | <0.001 | 612 | Right lenticular nucleus, putamen, BA 48 (328)  Right striatum (225) |
| ***SCZ < HCs*** | | | | | |
| Right postcentral gyrus, BA 3 | 54, -16, 42 | -7.144 | <0.001 | 676 | Right precentral gyrus, BA 6 (364)  Right postcentral gyrus, BA 3 (259) |
| Left postcentral gyrus, BA3 | -50, -16, 38 | -7.164 | <0.001 | 651 | Left postcentral gyrus, BA 3 (516)  Left precentral gyrus, BA 4 (53) |

*Abbreviations*: SCZ, schizophrenia; HCs, healthy controls; MNI, Montreal Neurological Institute; SDM, seed-based *d* mapping; BA, Brodmann area.

**Table S14. Results of heterogeneity, funnel plots and Egger’s test for resting-state functional brain activity and GMV meta-analysis**

| **Brain regions** | **MNI coordinates** | **Heterogeneity (*I^2^*), Jackknife sensitivity** | **Funnel plots** | **Egger’s test** |
| --- | --- | --- | --- | --- |
| **Resting-state functional activity alterations in FDRs-SCZ** | | | | |
| Left anterior cingulate / paracingulate gyri | -2, 28, 24 | 6.91%, 15/15 | 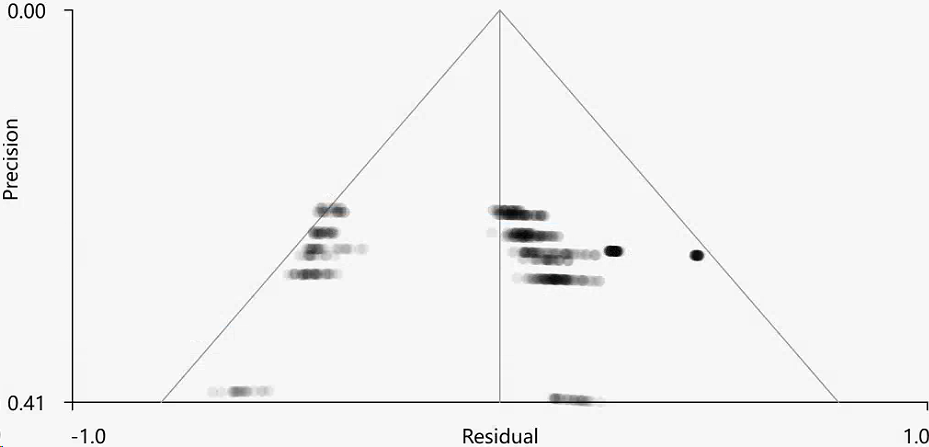 | *p* = 0.895 |
| **Resting-state functional activity alterations in SCZ** | | | | |
| Left striatum, BA 48 | -26, -2, -6 | 0.02%, 177/177 | 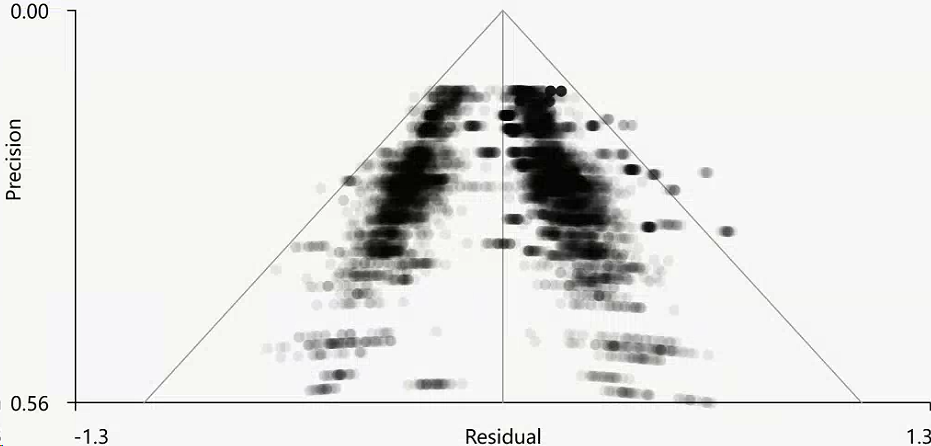 | *p* = 0.900 |
| Right striatum | 24, 2, 2 | 5.51%, 177/177 | 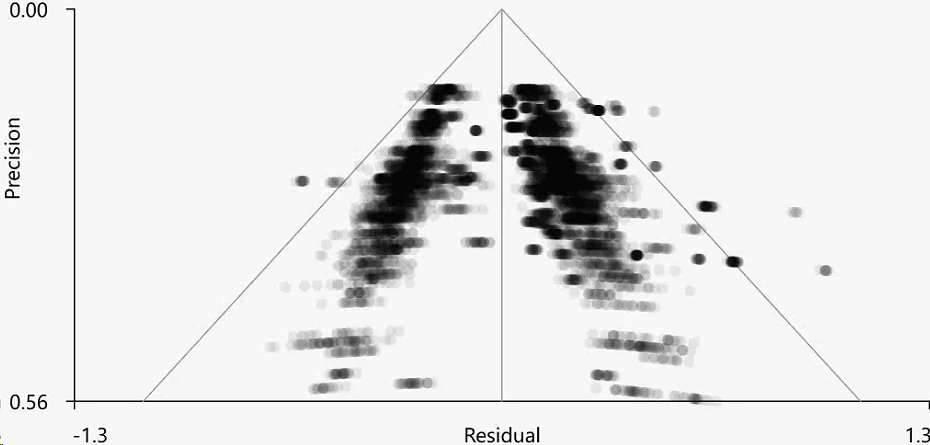 | *p* = 0.865 |
| Right superior frontal gyrus, medial orbital, BA 11 | 2, 50, -12 | 6.40%, 177/177 | 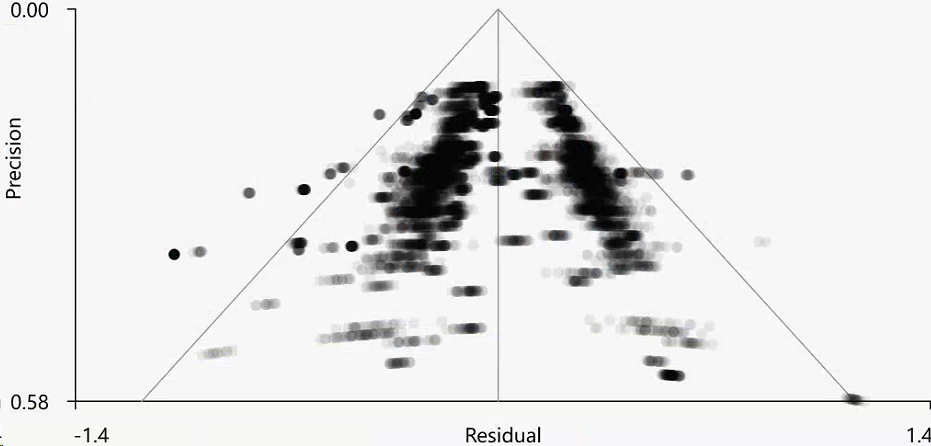 | *p* = 0.998 |
| Right postcentral gyrus, BA 3 | 46, -16, 38 | 4.03%, 177/177 | 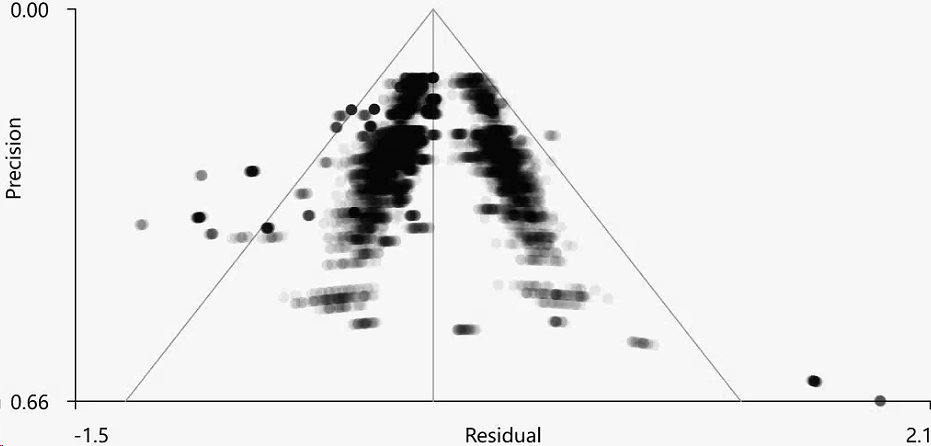 | *p* = 0.915 |
| Left postcentral gyrus, BA 3 | -58, -18, 36 | 16.60%, 177/177 | 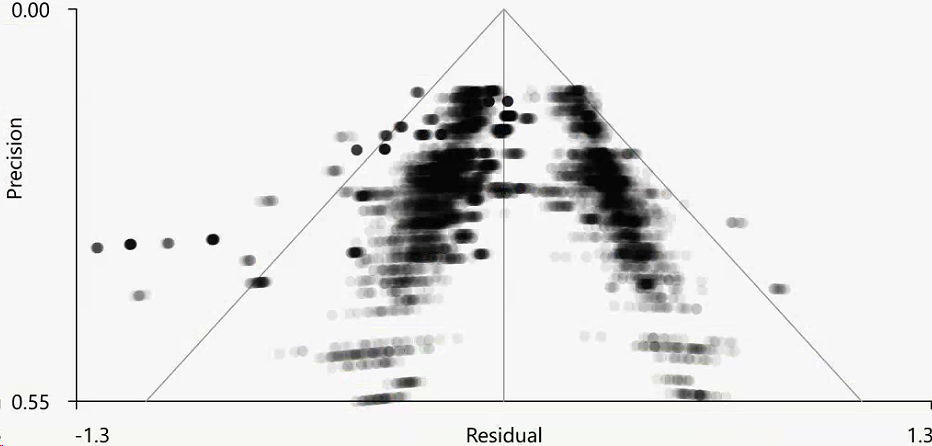 | *p =* 0.747 |
| Right middle temporal gyrus, BA 37 | 52, -64, 0 | 38.38%, 177/177 | 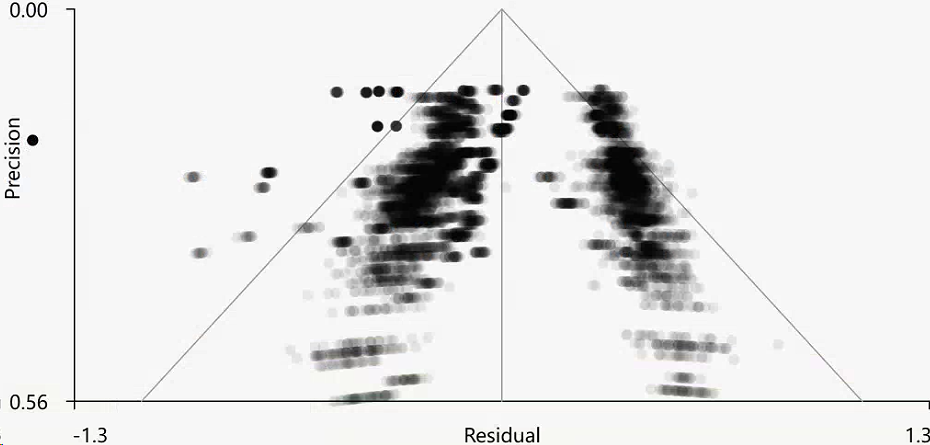 | *p =* 0.830 |
| **GMV alterations in SCZ** | | | | |
| Left inferior frontal gyrus, BA 45 | -46, 16, 4 | 9.60%, 136/136 | 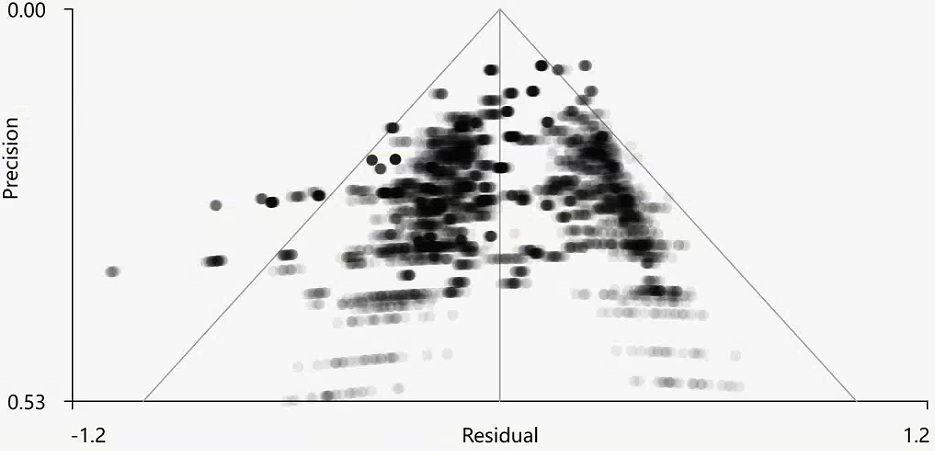 | *p =* 0.156 |
| Right inferior frontal gyrus, BA 48 | 48, 10, 4 | 14.43%, 136/136 | 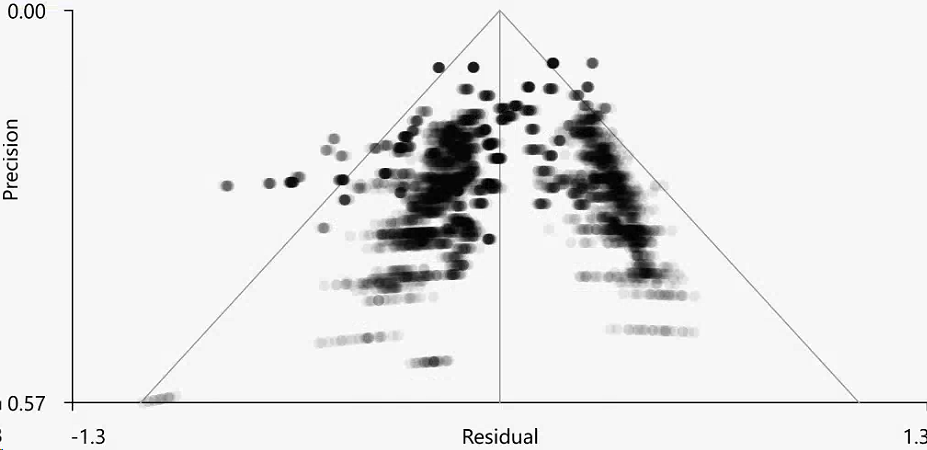 | *p =* 0.516 |
| Left superior frontal gyrus, medial, BA 10 | -6, 44, -8 | 31.28%, 136/136 | 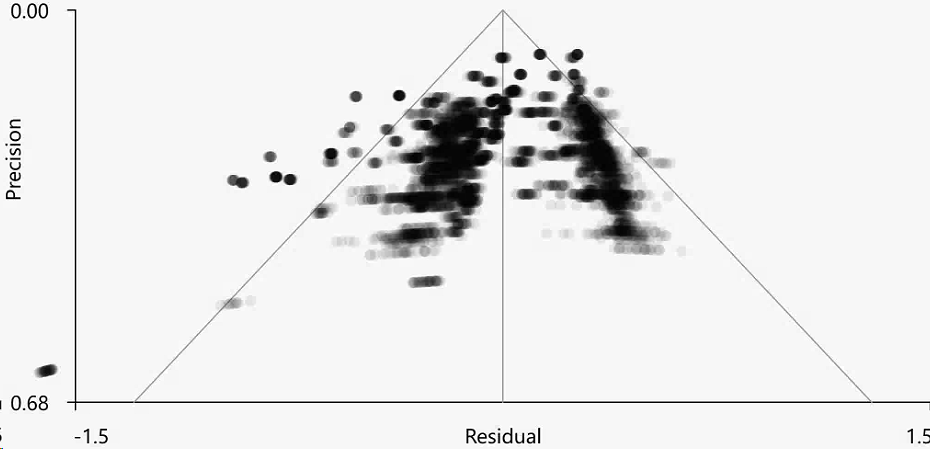 | *p =* 0.152 |
| Right median cingulate / paracingulate gyri, BA 23 | 2, -30, 36 | 36.39%, 136/136 | 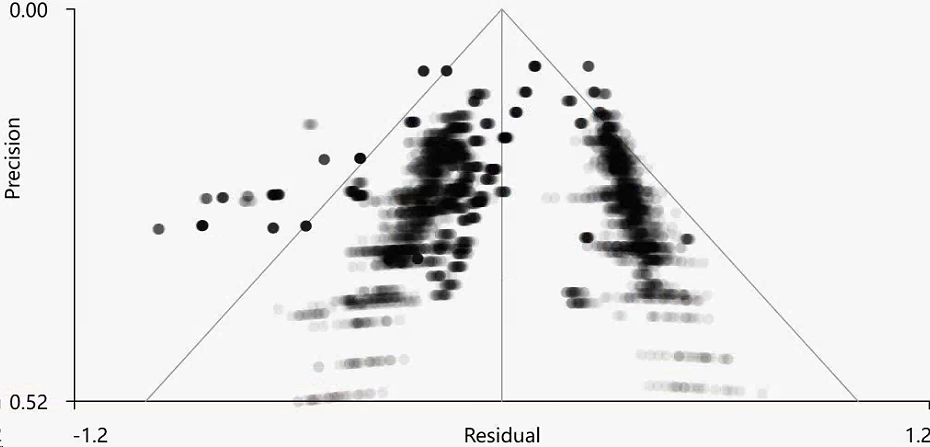 | *p* = 0.640 |
| Left fusiform gyrus, BA 37 | -26, -50, -16 | 10.70%, 136/136 | 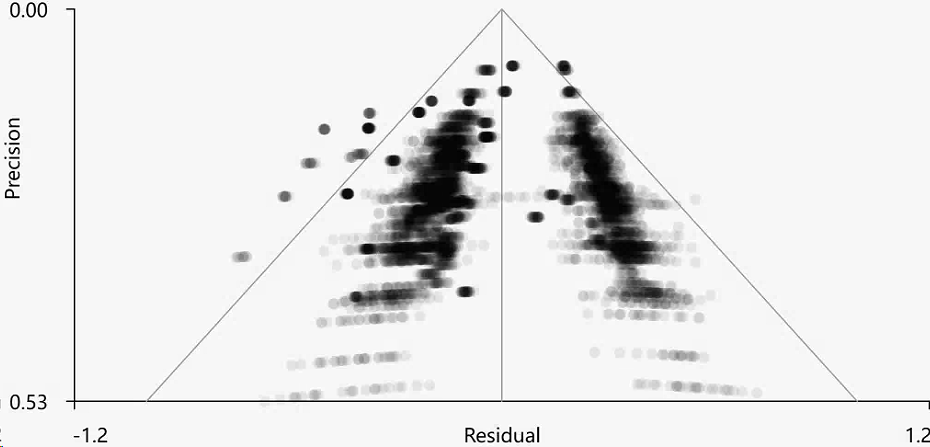 | *p* = 0.942 |

*Abbreviations*: FDRs-SCZ, first-degree relatives of SCZ; SCZ, schizophrenia; GMV, gray matter volume.

**Table S15.** Functional enrichment results of the genes related to brain changes in FDRs-SCZ and SCZ

| **Category** | **ID** | **Name** | P value | q value (FDR-BH correction) |
| --- | --- | --- | --- | --- |
| **Enrichment results of the genes related to functional changes in FDRs-SCZ** | | | | |
| GO: Biological Process | GO:0071804 | cellular potassium ion transport | 5.75E-08 | 7.88E-04 |
| GO: Biological Process | GO:0071805 | potassium ion transmembrane transport | 5.75E-08 | 3.94E-04 |
| GO: Biological Process | GO:0098662 | inorganic cation transmembrane transport | 6.98E-08 | 3.19E-04 |
| GO: Molecular Function | GO:0005261 | cation channel activity | 2.28E-12 | 8.85E-09 |
| GO: Cellular Component | GO:0034703 | cation channel complex | 3.48E-09 | 6.18E-06 |
| GO: Cellular Component | GO:0034702 | ion channel complex | 7.36E-09 | 6.55E-06 |
| **Enrichment results of the genes related to functional changes in SCZ** | | | | |
| GO: Biological Process | GO:0071804 | cellular potassium ion transport | 2.97E-11 | 4.06E-07 |
| GO: Biological Process | GO:0071805 | potassium ion transmembrane transport | 2.97E-11 | 2.03E-07 |
| GO: Biological Process | GO:0006813 | potassium ion transport | 7.89E-11 | 3.60E-07 |
| GO: Molecular Function | GO:0022839 | ion gated channel activity | 1.52E-12 | 5.91E-09 |
| GO: Molecular Function | GO:0022836 | gated channel activity | 4.81E-12 | 9.34E-09 |
| GO: Cellular Component | GO:1902495 | transmembrane transporter complex | 4.80E-13 | 8.54E-10 |
| GO: Cellular Component | GO:1990351 | transporter complex | 1.24E-12 | 1.10E-09 |
| GO: Cellular Component | GO:0034702 | ion channel complex | 2.91E-12 | 1.73E-09 |

Abbreviations: FDRs-SCZ, first-degree relatives of schizophrenia; SCZ, schizophrenia; GO, gene ontology; FDR-BH, Benjamini and Hochberg false discovery.

**Figure S1. GMV differences between SCZ and HCs.**

**
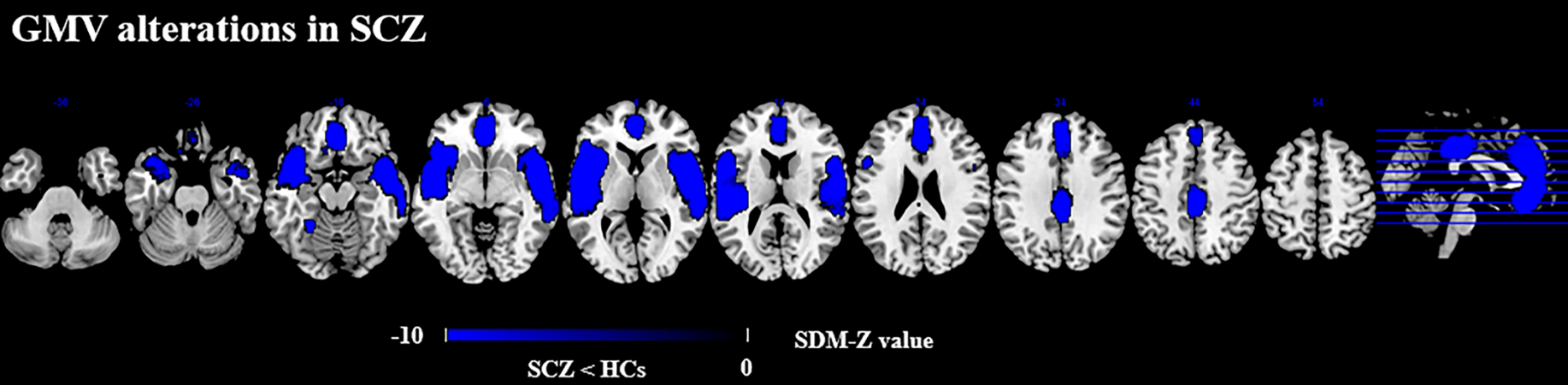
**

Areas with decreased GMV value are displayed in blue. The color bar indicates the maximum and minimum SDM-Z values. Abbreviations: GMV, gray matter volume; SCZ, schizophrenia; HCs, healthy controls; SDM, seed-based d mapping.

**Figure S2. Overlapping of abnormal resting-state functional brain activity and GMV in SCZ.**

**
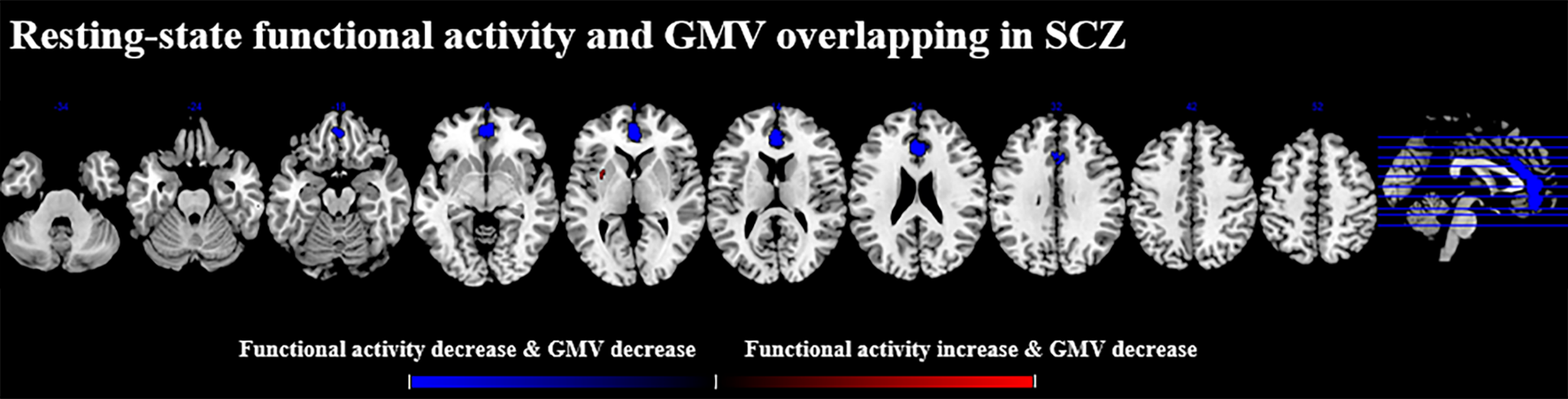
**

GMV, gray matter volume; SCZ, schizophrenia; HCs, healthy controls.

**Figure S3. Resting-state functional brain activity alterations in medicated patients with SCZ and chronic patients with SCZ.**

**
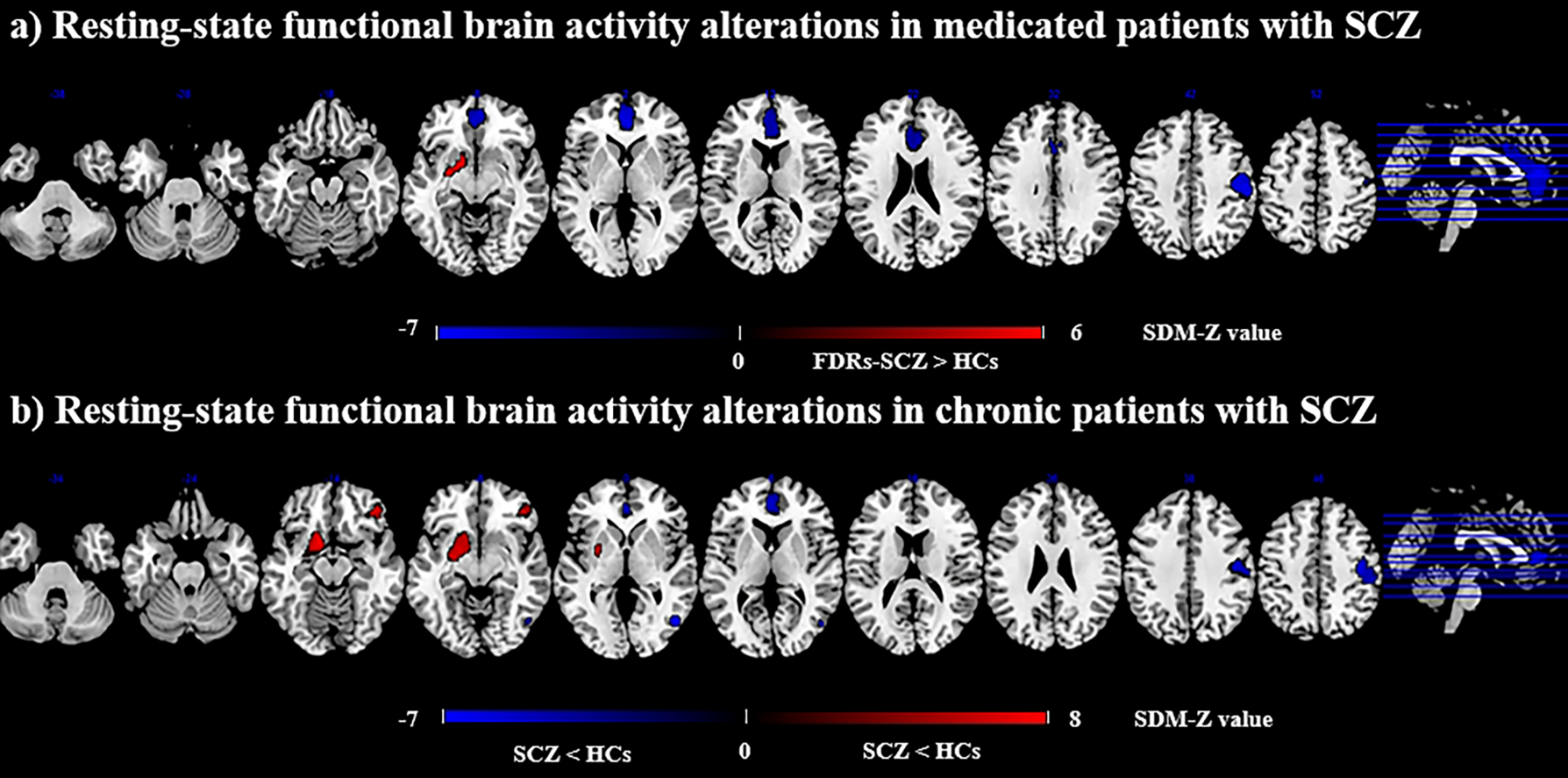
**

Areas with decreased resting-state functional brain activity value are displayed in blue, and areas with increased resting-state functional brain activity value are displayed in red. The color bar indicates the maximum and minimum SDM-Z values. *Abbreviations*: SCZ, schizophrenia; SDM, seed-based *d* mapping.

**Figure S4. Subgroup of different resting-state functional imaging methodology in FDRs-SCZ.**

**
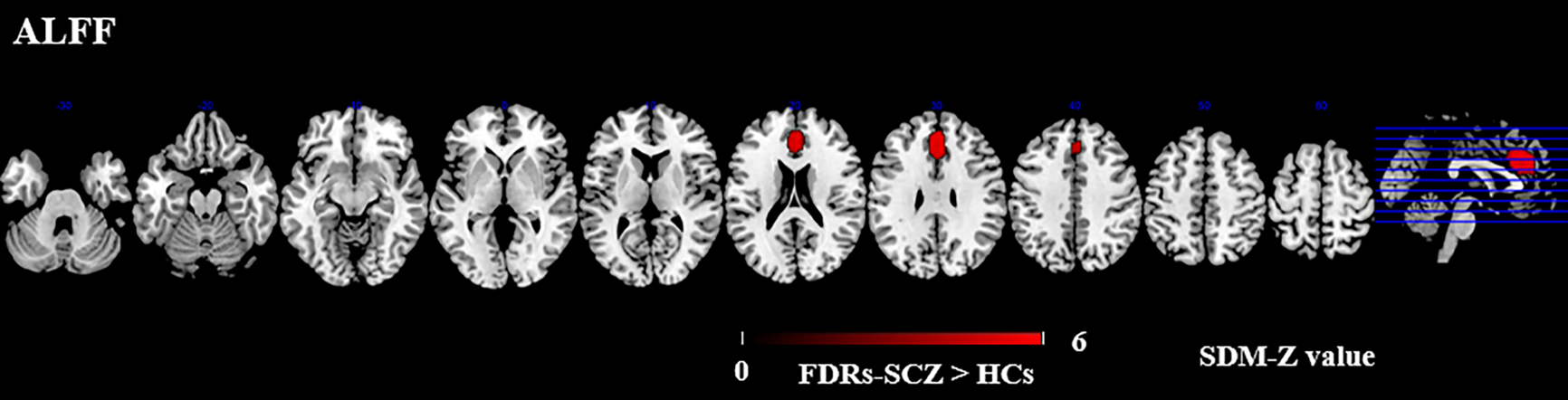
**

Areas with decreased resting-state functional brain activity value are displayed in blue, and areas with increased resting-state functional brain activity value are displayed in red. The color bar indicates the maximum and minimum SDM-Z values. *Abbreviations*: FDRs-SCZ, first-degree relatives of probands with schizophrenia; ALFF, amplitude of low-frequency signal fluctuations;SDM, seed-based *d* mapping.

**Figure S5. Subgroup of different resting-state functional imaging methodology in SCZ.**

**
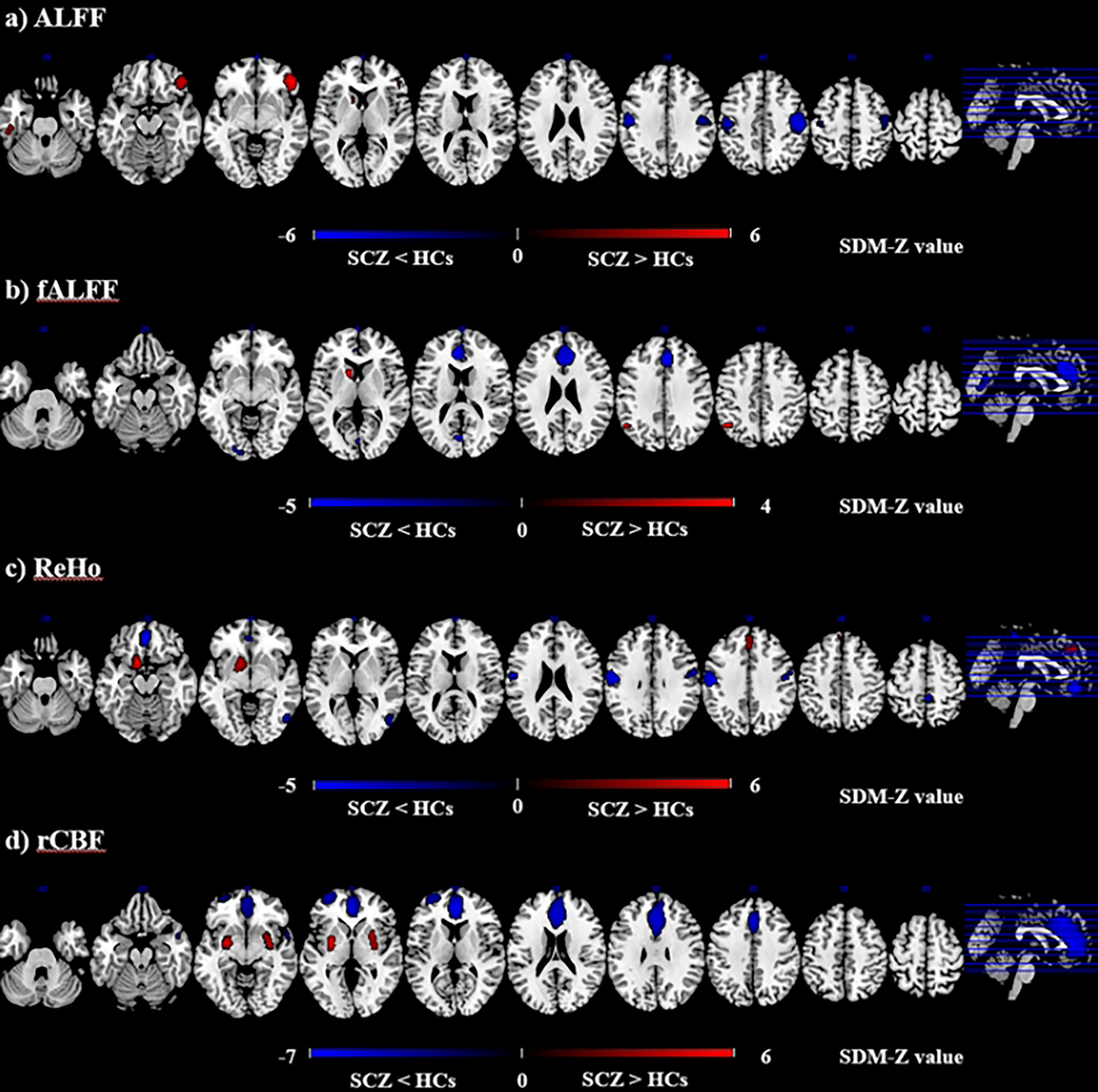
**a**)** ALFF alterations in SCZ; b) fALFF alterations in SCZ; c) ReHo alterations in SCZ; d) rCBF alterations in SCZ. Areas with decreased resting-state functional brain activity value are displayed in blue, and areas with increased resting-state functional brain activity value are displayed in red. The color bar indicates the maximum and minimum SDM-Z values. *Abbreviations*: SCZ, schizophrenia; ALFF, amplitude of low-frequency signal fluctuations; fALFF, fractional amplitude of low-frequency fluctuations; ReHo, regional homogeneity; rCBF, regional cerebral blood flow; SDM, seed-based *d* mapping.

**Figure S6. Results of resting-state functional brain activity alterations in SCZ subgroup (excluded the studies of insufficient number of subjects [n < 10] or uncorrected for statistics).**

**
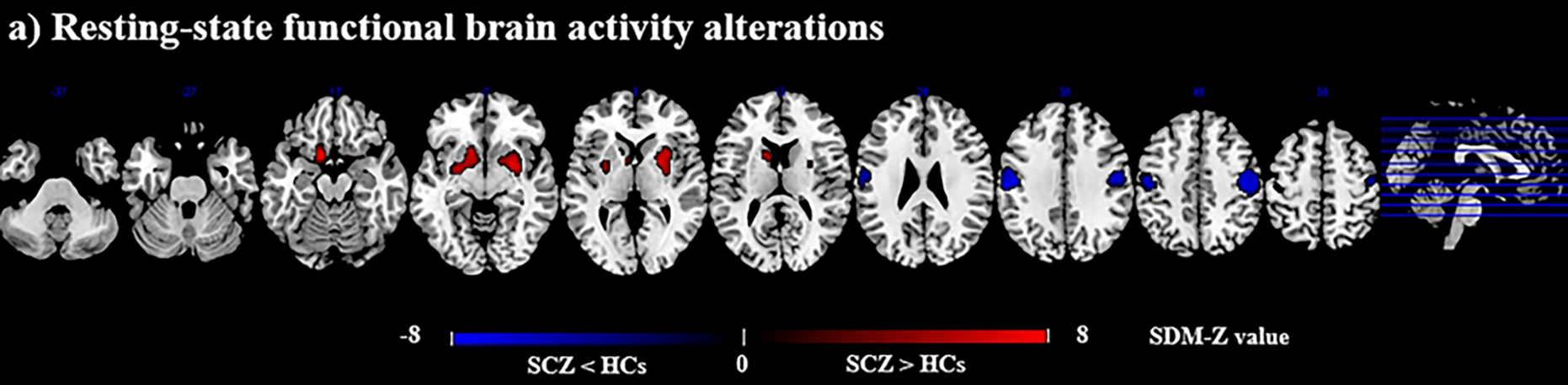
**

Areas with decreased resting-state functional brain activity value are displayed in blue, areas with increased resting-state functional brain activity value are displayed in red. The color bar indicates the maximum and minimum SDM-Z values. *Abbreviations*: SCZ, schizophrenia; SDM, seed-based *d* mapping.

**References:**

1. Arloth J, Bader DM, Röh S, Altmann A. Re-Annotator: Annotation Pipeline for Microarray Probe Sequences. *PLoS One.* 2015;10(10):e0139516.

2. Hawrylycz MJ, Lein ES, Guillozet-Bongaarts AL, et al. An anatomically comprehensive atlas of the adult human brain transcriptome. *Nature.* 2012;489(7416):391-399.

3. Fang Q, Cai H, Jiang P, et al. Transcriptional substrates of brain structural and functional impairments in drug-naive first-episode patients with major depressive disorder. *J Affect Disord.* 2023;325:522-533.

4. Xu X, Li Q, Qian Y, et al. Genetic mechanisms underlying gray matter volume changes in patients with drug-naive first-episode schizophrenia. *Cerebral cortex (New York, NY : 1991).* 2023;33(5):2328-2341.

5. Xue K, Guo L, Zhu W, et al. Transcriptional signatures of the cortical morphometric similarity network gradient in first-episode, treatment-naive major depressive disorder. *Neuropsychopharmacology : official publication of the American College of Neuropsychopharmacology.* 2023;48(3):518-528.

6. Abdi H, Williams LJ. Partial least squares methods: partial least squares correlation and partial least square regression. *Methods Mol Biol.* 2013;930:549-579.

7. Xia M, Liu J, Mechelli A, et al. Connectome gradient dysfunction in major depression and its association with gene expression profiles and treatment outcomes. *Mol Psychiatry.* 2022;27(3):1384-1393.

8. Burt JB, Helmer M, Shinn M, Anticevic A, Murray JD. Generative modeling of brain maps with spatial autocorrelation. *Neuroimage.* 2020;220:117038.

9. Fiore A, Preziosa P, Tedone N, et al. Correspondence among gray matter atrophy and atlas-based neurotransmitter maps is clinically relevant in multiple sclerosis. *Molecular psychiatry.* 2023;28(4):1770-1782.

10. Dukart J, Holiga S, Rullmann M, et al. JuSpace: A tool for spatial correlation analyses of magnetic resonance imaging data with nuclear imaging derived neurotransmitter maps. *Human brain mapping.* 2021;42(3):555-566.

11. Guo W, Su Q, Yao D, et al. Decreased regional activity of default-mode network in unaffected siblings of schizophrenia patients at rest. *European neuropsychopharmacology : the journal of the European College of Neuropsychopharmacology.* 2014;24(4):545-552.

12. Guo W, Song Y, Liu F, et al. Dissociation of functional and anatomical brain abnormalities in unaffected siblings of schizophrenia patients. *Clinical neurophysiology : official journal of the International Federation of Clinical Neurophysiology.* 2015;126(5):927-932.

13. Guo W, Liu F, Chen J, et al. Hyperactivity of the default-mode network in first-episode, drug-naive schizophrenia at rest revealed by family-based case-control and traditional case-control designs. *Medicine.* 2017;96(13):e6223.

14. Liao H, Wang L, Zhou B, et al. A resting-state functional magnetic resonance imaging study on the first-degree relatives of persons with schizophrenia. *Brain imaging and behavior.* 2012;6(3):397-403.

15. Liu C, Xue Z, Palaniyappan L, et al. Abnormally increased and incoherent resting-state activity is shared between patients with schizophrenia and their unaffected siblings. *Schizophrenia research.* 2016;171(1-3):158-165.

16. Lui S, Yao L, Xiao Y, et al. Resting-state brain function in schizophrenia and psychotic bipolar probands and their first-degree relatives. *Psychological medicine.* 2015;45(1):97-108.

17. Tang Y, Chen K, Zhou Y, et al. Neural activity changes in unaffected children of patients with schizophrenia: A resting-state fMRI study. *Schizophrenia research.* 2015;168(1-2):360-365.

18. Chen K. A resting--state functional magnetic resonance imaging study in offspring of schizophrenia subjects. *Chinese Medical Sciences University.* 2013.

19. Tian L, Yan H, Zhao Q, Zhang Y, Yue W, Zhang D. Amplitude of low-frequency fluctuations of the first-degree relatives of schizophrenia patients: a resting-state functional magnetic resonance imaging study. *Chin J Psychiatry.* 2014;47(3):137-141.

20. Wang J. The analysis of clinical characters and MRI study on brain functional and structural changes in patients with treatment-resistant schizophrenia and their relatives. *Central South University.* 2005.

21. Wang Y, Wang D, Wei S, Wang F, Tang Y. A resting—state function magnetic imaging study in first-episode drug-naive schizophrenia and offspring of schizophrenia patient. *ChinJ Nerv Ment Dis.* 2016;42(6):342-346.

22. Wang Y, Wang X, Long Y, et al. Family-based case-control study of regional homogeneity abnormalities in resting-state patients with first-episode, drug-naïve schizophrenia. *Chin J Psychiatry.* 2018;51(4):235-241.

23. Wang D, Jiang X, Sun T, Tang Y. Regional homogeneity of brain in schizophrenics and the individuals with high genetic risk of schizophrenia by resting-state functional magnetic resonance imaging. *Journal of China Medical University.* 2022;50(4):318-321.

24. Algumaei AH, Algunaid RF, Rushdi MA, Yassine IA. Feature and decision-level fusion for schizophrenia detection based on resting-state fMRI data. *PloS one.* 2022;17(5 May).

25. Alonso-Solís A, Vives-Gilabert Y, Portella MJ, et al. Altered amplitude of low frequency fluctuations in schizophrenia patients with persistent auditory verbal hallucinations. *Schizophrenia research.* 2017;189:97-103.

26. Andreasen NC, O'Leary DS, Flaum M, et al. Hypofrontality in schizophrenia: distributed dysfunctional circuits in neuroleptic-naïve patients. *Lancet (London, England).* 1997;349(9067):1730-1734.

27. Bai Y, Wang W, Xu J, et al. Altered resting-state regional homogeneity after 13 weeks of paliperidone injection treatment in schizophrenia patients. *Psychiatry research Neuroimaging.* 2016;258:37-43.

28. Bai Y, Yng F, Sun J, Vogel, Hua J, Yu D. Electrostatic Gunctional Magmetic Resonance of Patients with Chronic Schizophrenia with Impulsive Attack Behavior. *Inner Mongolia Med J.* 2018;50(12):1411-1414.

29. Blackwood DH, Glabus MF, Dunan J, O'Carroll RE, Muir WJ, Ebmeier KP. Altered cerebral perfusion measured by SPECT in relatives of patients with schizophrenia. Correlations with memory and P300. *The British journal of psychiatry : the journal of mental science.* 1999;175:357-366.

30. Boyer L, Richieri R, Faget C, et al. Functional involvement of superior temporal sulcus in quality of life of patients with schizophrenia. *Psychiatry research.* 2012;202(2):155-160.

31. Cai Q, Chen Y, Li J, Zhao Z, Wang Y. Study on the identification of schizophrenia patients with abnormal amplitude of resting low frequency oscillation. *Chinese Journal of Magnetic Resonance Imaging.* 2021;12(10):45-48.

32. Chen C. Schizophrenic patients with or without auditory hallucination in the study of fraction amplitude of low-frequency fluctuation of resting state functional magnetic resonance imaging. *Chin J Psychiatry.* 2015;Vol. 48, No. 4(4):232-238.

33. Chen F, Yao Q, Zhan D, et al. The abnomalities of amplitude of low-frequency fluctuation in the resting-state functional magnetic resonance imaging of first-episode medication-naive schizophrenia before and after antipsychotic drugs therapy. *Chin J Psychiatry.* 2017;50(02):133-138.

34. Chen C, Yao J, Lv Y, et al. Aberrant Functional Connectivity of the Orbitofrontal Cortex Is Associated With Excited Symptoms in First-Episode Drug-Naïve Patients With Schizophrenia. *Frontiers in Psychiatry.* 2022;13.

35. Cheng D. *A comparative study of resting-state function MRI and 3D ASL in schizophrenia patients of different subtype* [硕士]: Southern Medical University, Southern Medical University; 2017.

36. Cheng L. *Resting state fMRI study of fractional amplitude of low-frequency fluctuations and functional connectivity in early and adult onset schizophrenia* [硕士], Shanxi Medical University; 2017.

37. Clark C, Kopala L, Li DK, Hurwitz T. Regional cerebral glucose metabolism in never-medicated patients with schizophrenia. *Canadian journal of psychiatry Revue canadienne de psychiatrie.* 2001;46(4):340-345.

38. Cui W, Zhou Q, Wang F, Tang Y. Comparison of Amplitude of Low Frequency Fluctuation between Schizophrenia and Bipolar Disorder Patient: a Resting-state Functional Magnetic Resonance Imaging Study. 2016;45(11):977-981+984.

39. Cui LB, Liu K, Li C, et al. Putamen-related regional and network functional deficits in first-episode schizophrenia with auditory verbal hallucinations. *Schizophrenia research.* 2016;173(1-2):13-22.

40. Cui LB, Wang LX, Tian P, et al. Aberrant perfusion and its connectivity within default mode network of first-episode drug-naïve schizophrenia patients and their unaffected first-degree relatives. *Scientific reports.* 2017;7(1):16201.

41. Cui LB, Chen G, Xu ZL, et al. Cerebral blood flow and its connectivity features of auditory verbal hallucinations in schizophrenia: A perfusion study. *Psychiatry research Neuroimaging.* 2017;260:53-61.

42. Deng X. *Comparative Study of Regional Homogeneity in First-Episode Schizophrenia and Individuals with a Genetic High Risk of Schizophrenia: A Resting-State Functional Magnetic Resonance Imaging Study* [硕士], China Medical University; 2018.

43. Deng L, Zhang L, Luo YL, Liu X, Hu J. Study of ampIitude of low frequency fluctuation in first-episode and untreated patients with schizophrenia. *Journal of Clinical Psychiatry.* 2019;29(4):243-246.

44. Deng L, Wei W, Qiao C, Yin Y, Qian L, Li T. Frequency-Specific Alterations of Spontaneous Brain Activity in First-Episode Drug-Naïve Schizophrenia. *J Sichuan Univ (Med Sci).* 2023;54(2):281-286.

45. Dong DB, Duan MJ, Wang YL, et al. Reconfiguration of Dynamic Functional Connectivity in Sensory and Perceptual System in Schizophrenia. *Cerebral Cortex.* 2019;29(8):3577-3589.

46. Dye SM, Spence SA, Bench CJ, et al. No evidence for left superior temporal dysfunction in asymptomatic schizophrenia and bipolar disorder. PET study of verbal fluency. *The British journal of psychiatry : the journal of mental science.* 1999;175:367-374.

47. Faget-Agius C, Boyer L, Padovani R, et al. Schizophrenia with preserved insight is associated with increased perfusion of the precuneus. *J Psychiatry Neurosci.* 2012;37(5):297-304.

48. Fang X, Zhang R, Bao C, et al. Abnormal regional homogeneity (ReHo) and fractional amplitude of low frequency fluctuations (fALFF) in first-episode drug-naïve schizophrenia patients comorbid with depression. *Brain imaging and behavior.* 2021;15(5):2627-2636.

49. Feng A, Luo N, Zhao W, et al. Multimodal brain deficits shared in early-onset and adult-onset schizophrenia predict positive symptoms regardless of illness stage. *Human brain mapping.* 2022;43(11):3486-3497.

50. Foucher JR, Zhang YF, Roser M, et al. A double dissociation between two psychotic phenotypes: Periodic catatonia and cataphasia. *Progress in neuro-psychopharmacology & biological psychiatry.* 2018;86:363-369.

51. Gao B, Wang Y, Liu W, et al. Spontaneous Activity Associated with Delusions of Schizophrenia in the Left Medial Superior Frontal Gyrus: A Resting-State fMRI Study. *PLoS One.* 2015;10(7):e0133766.

52. Gao S, Lu S, Shi X, et al. Distinguishing between treatment-resistant and non-treatment-resistant schizophrenia using regional homogeneity. *Frontiers in Psychiatry.* 2018;9(AUG).

53. Gao S, Ming Y, Wang J, et al. Enhanced Prefrontal Regional Homogeneity and Its Correlations With Cognitive Dysfunction/Psychopathology in Patients With First-Diagnosed and Drug-Naive Schizophrenia. *Frontiers in psychiatry.* 2020;11:580570.

54. Gao Y, Tong X, Hu J, et al. Decreased resting-state neural signal in the left angular gyrus as a potential neuroimaging biomarker of schizophrenia: An amplitude of low-frequency fluctuation and support vector machine analysis. *Frontiers in Psychiatry.* 2022;13.

55. Guo W, Zhang F, Liu F, et al. Cerebellar abnormalities in first-episode, drug-naive schizophrenia at rest. *Psychiatry research Neuroimaging.* 2018;276:73-79.

56. Hare SM, Ford JM, Ahmadi A, et al. Modality-Dependent Impact of Hallucinations on Low-Frequency Fluctuations in Schizophrenia. *Bulletin.* 2017;43(2):389-396.

57. Horáček J, Kopeček M, Španiel F, et al. Resting regional brain metabolism in patients with schizophrenia. 18FDG PET study. *Psychiatrie.* 2004;8(SUPPL. 3):43-50.

58. Horga G, Fernández-Egea E, Mané A, et al. Brain metabolism during hallucination-like auditory stimulation in schizophrenia. *PloS one.* 2014;9(1):e84987.

59. Hu ML, Zong XF, Zheng JJ, et al. Short-term Effects of Risperidone Monotherapy on Spontaneous Brain Activity in First-episode Treatment-naive Schizophrenia Patients: A Longitudinal fMRI Study. *Sci Rep.* 2016;6:34287.

60. Huang XQ, Lui S, Deng W, et al. Localization of cerebral functional deficits in treatment-naive, first-episode schizophrenia using resting-state fMRI. *NeuroImage.* 2010;49(4):2901-2906.

61. Huang Y, Wang W, Hei G, et al. Altered regional homogeneity and cognitive impairments in first-episode schizophrenia: A resting-state fMRI study. *Asian journal of psychiatry.* 2022;71.

62. Jin K, Xu D, Shen Z, et al. Distinguishing hypochondriasis and schizophrenia using regional homogeneity: a resting-state fMRI study and support vector machine analysis. *Acta neuropsychiatrica.* 2021;33(4):182-190.

63. Jing H, Zhang C, Yan H, et al. Deviant spontaneous neural activity as a potential early-response predictor for therapeutic interventions in patients with schizophrenia. *Frontiers in neuroscience.* 2023;17:1243168.

64. Kanahara N, Shimizu E, Sekine Y, et al. Does hypofrontality expand to global brain area in progression of schizophrenia?: a cross-sectional study between first-episode and chronic schizophrenia. *Progress in neuro-psychopharmacology & biological psychiatry.* 2009;33(3):410-415.

65. Kanahara N, Sekine Y, Haraguchi T, et al. Orbitofrontal cortex abnormality and deficit schizophrenia. *Schizophrenia research.* 2013;143(2-3):246-252.

66. Kawakami K, Wake R, Miyaoka T, Furuya M, Liaury K, Horiguchi J. The effects of aging on changes in regional cerebral blood flow in schizophrenia. *Neuropsychobiology.* 2014;69(4):202-209.

67. Kim JJ, Mohamed S, Andreasen NC, et al. Regional neural dysfunctions in chronic schizophrenia studied with positron emission tomography. *The American journal of psychiatry.* 2000;157(4):542-548.

68. Kindler J, Schultze-Lutter F, Hauf M, et al. Increased Striatal and Reduced Prefrontal Cerebral Blood Flow in Clinical High Risk for Psychosis. *Schizophrenia bulletin.* 2018;44(1):182-192.

69. Kuang Q, Liu Y, Song Z, et al. The correlation between cognitive function and fraction amplitude of low-frequency fluctuation of patients with first-episode schizophrenia. *Chin J Ment Dis.* 2019;45(09):529-534.

70. Kuang QJ, Zhou SM, Liu Y, et al. Prediction of Facial Emotion Recognition Ability in Patients With First-Episode Schizophrenia Using Amplitude of Low-Frequency Fluctuation-Based Support Vector Regression Model. *Frontiers in Psychiatry.* 2022;13.

71. Lei W, Li M, Deng W, et al. Sex-Specific Patterns of Aberrant Brain Function in First-Episode Treatment-Naive Patients with Schizophrenia. *International journal of molecular sciences.* 2015;16(7):16125-16143.

72. Li F, Lui S, Yao L, et al. Longitudinal Changes in Resting-State Cerebral Activity in Patients with First-Episode Schizophrenia: A 1-Year Follow-up Functional MR Imaging Study. *Radiology.* 2016;279(3):867-875.

73. Li Y, Fan Y, Ma Q, et al. An exploratory study on brain imaging and inflammatory cytokines in first-episode schizophrenia. *Journal of Xi'an Jiaotong University.* 2020;41(06):910-914.

74. Li H, She S, Kuang Q, Zheng Y. Prediction of visual information processing speed in patients with early schizophrenia using amplitude of low -frequency fluctuation based resting -state functional magnetic resonance imaging. *Chin J Nerv Ment Dis.* 2021;47(2):5.

75. Li X, Liu Q, Chen Z, et al. Abnormalities of Regional Brain Activity in Patients With Schizophrenia: A Longitudinal Resting-State fMRI Study. *Schizophr Bull.* 2023;49(5):1336-1344.

76. Lian N, Lv H, Guo W, et al. A comparative study of magnetic resonance imaging on the gray matter and resting-state function in prodromal and first-episode schizophrenia. *American journal of medical genetics Part B, Neuropsychiatric genetics : the official publication of the International Society of Psychiatric Genetics.* 2018;177(6):537-545.

77. Liang J, XIE S, DU J, Lv W. Regional homogeneity of functional magnetic resonance imaging in male schizophrenia patients with delusion symptoms. *The Journal of clinical psychiatry.* 2013;23(006):379-382.

78. Liang J, XIE S. Changes of resting state functional magnetic resonance brain sPontaneous low-frequency amPlitude in male Patients with Paranoid schizoPhrenia. *The Journal of clinical psychiatry.* 2014;24(02):77-79.

79. Liu H, Liu Z, Liang M, et al. Decreased regional homogeneity in schizophrenia: a resting state functional magnetic resonance imaging study. *Neuroreport.* 2006;17(1):19-22.

80. Liu H, Fan G, Xu K, Li H, Shao J. Amplitude of low-frequency fluctuation study of resting-state functional MRI in schizophrenia. *Chin J Med Imaging Technol.* 2010;26(09):1659-1662.

81. Liu L, Gao Y, Xie S, Wang R. Resting state functional magnetic resonance imageing study with regional homogeneity in first-episode schizophrenic patients. *Radio Practic Jul.* 2017;32(7).

82. Lui S, Li T, Deng W, et al. Short-term effects of antipsychotic treatment on cerebral function in drug-naive first-episode schizophrenia revealed by "resting state" functional magnetic resonance imaging. *Arch Gen Psychiatry.* 2010;67(8):783-792.

83. Ma X, Yang WFZ, Zheng W, et al. Neuronal dysfunction in individuals at early stage of schizophrenia, A resting-state fMRI study. *Psychiatry research.* 2023;322:115123.

84. Malaspina D, Harkavy-Friedman J, Corcoran C, et al. Resting neural activity distinguishes subgroups of schizophrenia patients. *Biological psychiatry.* 2004;56(12):931-937.

85. Mathew RJ, Duncan GC, Weinman ML, Barr DL. Regional cerebral blood flow in schizophrenia. *Archives of general psychiatry.* 1982;39(10):1121-1124.

86. Mauri MC, Gaietta M, Dragogna F, Valli I, Cerveri G, Marotta G. Hallucinatory disorder, an original clinical picture? Clinical and imaging data. *Prog Neuropsychopharmacol Biol Psychiatry.* 2008;32(2):523-530.

87. Ota M, Ishikawa M, Sato N, et al. Pseudo-continuous arterial spin labeling MRI study of schizophrenic patients. *Schizophrenia research.* 2014;154(1-3):113-118.

88. Park IH, Kim JJ, Chun J, et al. Medial prefrontal default-mode hypoactivity affecting trait physical anhedonia in schizophrenia. *Psychiatry research.* 2009;171(3):155-165.

89. Park JH, Hong JS, Kim SM, Min KJ, Chung US, Han DH. Effects of Amisulpride Adjunctive Therapy on Working Memory and Brain Metabolism in the Frontal Cortex of Patients with Schizophrenia: A Preliminary Positron Emission Tomography/Computerized Tomography Investigation. *Clinical Psychopharmacology and Neuroscience.* 2019;17(2):250-260.

90. Potkin SG, Alva G, Fleming K, et al. A PET study of the pathophysiology of negative symptoms in schizophrenia. *American Journal of Psychiatry.* 2002;159(2):227-237.

91. Ren W, Lui S, Deng W, et al. Anatomical and functional brain abnormalities in drug-naive first-episode schizophrenia. *The American journal of psychiatry.* 2013;170(11):1308-1316.

92. Salvador R, Landin-Romero R, Anguera M, et al. Non redundant functional brain connectivity in schizophrenia. *Brain imaging and behavior.* 2017;11(2):552-564.

93. Scheef L, Manka C, Daamen M, et al. Resting-state perfusion in nonmedicated schizophrenic patients: a continuous arterial spin-labeling 3.0-T MR study. *Radiology.* 2010;256(1):253-260.

94. Schneider K, Michels L, Hartmann-Riemer MN, et al. Cerebral blood flow in striatal regions is associated with apathy in patients with schizophrenia. *Journal of psychiatry & neuroscience : JPN.* 2019;44(2):102-110.

95. Shan XX, Liao RY, Ou YP, et al. Increased regional homogeneity modulated by metacognitive training predicts therapeutic efficacy in patients with schizophrenia. *European archives of psychiatry and clinical neuroscience.* 2020;271(4):783-798.

96. Shan X, Liao R, Ou Y, et al. Increased regional homogeneity modulated by metacognitive training predicts therapeutic efficacy in patients with schizophrenia. *European archives of psychiatry and clinical neuroscience.* 2021;271(4):783-798.

97. Shao T, Wang W, Hei G, et al. Identifying and revealing different brain neural activities of cognitive subtypes in early course schizophrenia. *Frontiers in molecular neuroscience.* 2022;15:983995.

98. Suazo V, Díez Á, Tamayo P, Montes C, Molina V. Limbic hyperactivity associated to verbal memory deficit in schizophrenia. *Journal of psychiatric research.* 2013;47(6):843-850.

99. Sui J, Pearlson GD, Du Y, et al. In search of multimodal neuroimaging biomarkers of cognitive deficits in schizophrenia. *Biological psychiatry.* 2015;78(11):794-804.

100. Sun H, Liu H, Zhang Y. Analysis of brain structure and function in patients with schizophrenia by low frequency amplitude combined with voxel-based morphological analysis. *Journal of Qiqihar Medical University.* 2020;41(20):3.

101. Sun D, Guo H, Womer FY, et al. Frontal-posterior functional imbalance and aberrant function developmental patterns in schizophrenia. *Translational psychiatry.* 2021;11(1).

102. Tan Z, Chen W, Pang Y, Xia J, Luo S. Applications of resting⁃state functional MRI in low⁃frequency fluctuation combined with voxel⁃based mor⁃ phometry in schizophrenia. 2017;33(14):2380-2385.

103. Tang YQ, Zhou Q, Chang M, et al. Altered functional connectivity and low-frequency signal fluctuations in early psychosis and genetic high risk. *Schizophrenia research.* 2019;210:172-179.

104. Tian H. *Application of ALFF and brain connections methods in brain imaging data analysis* [硕士], Hunan Normal University; 2018.

105. Tong W, Dong Z, Guo W, et al. Progressive Changes in Brain Regional Homogeneity Induced by Electroconvulsive Therapy Among Patients With Schizophrenia. *The journal of ECT.* 2022;38(2):117-123.

106. Tong X. An amplitude of low-frequency fluctuation and support vector machine analysis potential neuroimaging biomarker of schizophrenia. *Anhui Medical University.* 2023.

107. Turner JA, Damaraju E, van Erp TM, et al. A multi-site resting state fMRI study on the amplitude of low frequency fluctuations in schizophrenia. *Frontiers in Neuroscience.* 2013;7.

108. Wake R, Miyaoka T, Kawakami K, et al. Characteristic brain hypoperfusion by 99mTc-ECD single photon emission computed tomography (SPECT) in patients with the first-episode schizophrenia. *European psychiatry : the journal of the Association of European Psychiatrists.* 2010;25(6):361-365.

109. Wake R, Miyaoka T, Araki T, et al. Regional cerebral blood flow in late-onset schizophrenia: a SPECT study using 99mTc-ECD. *European archives of psychiatry and clinical neuroscience.* 2016;266(1):3-12.

110. Walther S, Federspiel A, Horn H, et al. Resting state cerebral blood flow and objective motor activity reveal basal ganglia dysfunction in schizophrenia. *Psychiatry Research-Neuroimaging.* 2011;192(2):117-124.

111. Walther S, Schäppi L, Federspiel A, et al. Resting-State Hyperperfusion of the Supplementary Motor Area in Catatonia. *Schizophrenia bulletin.* 2016;43(5):972-981.

112. Wang D, Jiang X, Sun T, Tang Y. Regional homogeneity of brain in schizophrenics and the individuals with high genetic risk of schizophrenia by resting-state functional magnetic resonance imaging. *Journal of China Medical University.* 2021;50(4):318-321.

113. Wang J. *The analysis of clinical characters and MRI study on brain functional and structural changes in patients with treatment-resistant schizophrenia and their relatives* 2009.

114. Wang Y. Study on low frequency amplitude of resting brain function in patients with paranoid schizophrenia. *Zhejiang clinical medicine.* 2019;21(8).

115. Wei YE, Chang M, Fay YW, et al. Local functional connectivity alterations in schizophrenia, bipolar disorder, and major depressive disorder. *Journal of affective disorders.* 2018;236:266-273.

116. Wu FC, Zhang Y, Yang YZ, et al. Structural and functional brain abnormalities in drug-naive, first-episode, and chronic patients with schizophrenia: a multimodal MRI study. *Neuropsychiatric Disease and Treatment.* 2018;14:2889-2904.

117. Wu RR, Ou YP, Liu F, et al. Reduced Brain Activity in the Right Putamen as an Early Predictor for Treatment Response in Drug-Naive, First-Episode Schizophrenia. *Frontiers in Psychiatry.* 2019;10.

118. Xie Y, Guan M, Wang Z, et al. rTMS Induces Brain Functional and Structural Alternations in Schizophrenia Patient With Auditory Verbal Hallucination. *Frontiers in neuroscience.* 2021;15:722894-722894.

119. Xie Y, Guan M, Wang Z, Ma Z, Fang P, Wang H. Cerebral blood flow changes in schizophrenia patients with auditory verbal hallucinations during low-frequency rTMS treatment. *European archives of psychiatry and clinical neuroscience.* 2023;273(8):1851-1861.

120. Xu X, Shi X, Peng Z, Wang X, Sun J, Yao H. A study of resting- state functional magnetic resonance imaging in treatment-refractory schizophrenia patients using regional homogeneity. *The Journal of clinical psychiatry.* 2015;25(06):377-380.

121. Xu L, Qin W, Zhuo C, Liu H, Zhu J, Yu C. Combination of volume and perfusion parameters reveals different types of grey matter changes in schizophrenia. *Scientific reports.* 2017;7(1):435.

122. Xu Z, Gao C, Zou F. Brain Resting-state MRI Features of Elderly Patients with First-episode Schizophrenia. *CHINESE JOURNAL OF CT AND MRI.* 2022;20(8):154.

123. Yan W, Zhang R, Zhou M, et al. Relationships between abnormal neural activities and cognitive impairments in patients with drug-naive first-episode schizophrenia. *BMC psychiatry.* 2020;20(1).

124. Yang F, Yang T, Kang C, et al. The chang of resting state functional magnetic resonance imaging in chronic schizophrenia patients combined riperidone treatment *The Journal of clinical psychiatry.* 2014;24(02):73-76.

125. Yang M, Jia X, Zhou H, et al. Brain dysfunction of methamphetamine-associated psychosis in resting state: Approaching schizophrenia and critical role of right superior temporal deficit. *Addiction biology.* 2021;26(6).

126. Yang F, Ma H, Yuan J, et al. Correlation of abnormalities in resting state fMRI with executive functioning in chronic schizophrenia. *Psychiatry research.* 2021;299.

127. Yang Y, Xue FF. Resting-state functional brain imaging of amplitude of low-frequency fluctuation in patients with refractory schizophrenia. *China Modern Doctor.* 2021;59(30):115-118,122,封113.

128. Yang W, Li Y, Xu L, et al. Multi-band low-frequency fluctuations of resting-state in patients with schizophrenia. *Chinese Mental Health Journal.* 2022;36(8):663-668.

129. Yang Y, Sun Y, Zhang Y, et al. Abnormal patterns of regional homogeneity and functional connectivity across the adolescent first-episode, adult first-episode and adult chronic schizophrenia. *NeuroImage Clinical.* 2022;36:103198-103198.

130. Yang P, Qiu J, Zhu X, Gao Z. Regional homogeneity and cognitive function in patients with chronic schizophrenia. *JOURNAL OF INTERNATIONAL PSYCHIATRY.* 2023;50(1):32.

131. Yin P, Zhao C, Li Y, Liu X, Chen L, Hong N. Changes in Brain Structure, Function, and Network Properties in Patients With First-Episode Schizophrenia Treated With Antipsychotics. *Frontiers in Psychiatry.* 2021;12.

132. Yu R, Hsieh MH, Wang HL, et al. Frequency dependent alterations in regional homogeneity of baseline brain activity in schizophrenia. *PloS one.* 2013;8(3):e57516.

133. Yu R, Chien YL, Wang HL, et al. Frequency-specific alternations in the amplitude of low-frequency fluctuations in schizophrenia. *Human brain mapping.* 2014;35(2):627-637.

134. Yu XM, Qiu LL, Huang HX, et al. Comparison of resting-state spontaneous brain activity between treatment-naive schizophrenia and obsessive-compulsive disorder. *BMC psychiatry.* 2021;21(1).

135. Yu X, Huang H, Tian L, et al. Contrast study using the resting-state fractional amplitude of low-frequency fluctuations between treatment-naive patients with schizophrenia and obsessive-compulsive disorder. *Chin J Magn Reson Imaging.* 2021;12(06):16-21.

136. Yu L, Guo L, Fang X, et al. Altered brain activity in the bilateral frontal cortices and neural correlation with cognitive impairment in schizophrenia. *Brain imaging and behavior.* 2021;16(1):415-423.

137. Zhang X, Li J, Liao J, et al. Abnormal gray matter volume and regional homogeneity of resting fMI in patients with schizophrenia and obsessive-compulsive disorder. 2017;31(10):768-774.

138. Zhang L. *The Correlation between the BDNF Gene Polymorphism and Resting-state Function MRI of Brain in Schizophrenia* [硕士], Xinxiang Medical University; 2018.

139. Zhang P, Li Y, Fan F, et al. Resting-state Brain Activity Changes Associated with Tardive Dyskinesia in Patients with Schizophrenia: Fractional Amplitude of Low-frequency Fluctuation Decreased in the Occipital Lobe. *Neuroscience.* 2018;385:237-245.

140. Zhang S, Hu Q, Tang T, et al. Changes in Gray Matter Density, Regional Homogeneity, and Functional Connectivity in Methamphetamine-Associated Psychosis: A Resting-State Functional Magnetic Resonance Imaging (fMRI) Study. *Medical science monitor : international medical journal of experimental and clinical research.* 2018;24:4020-4030.

141. Zhang R, Yan W, Lv L, Lu S, Xie S. Regional homogeneity and cognitive function in first-episode patients with schizophrenia. *The Journal of clinical psychiatry.* 2018;28.

142. Zhang P, Li Y, Fan F, et al. Altered amplitude of low-frequency fluctuations in patients with schizophrenia and tardive dyskinesia: A resting state fMRIstudy. *Chinese Mental Health Journa.* 2018;32(05):442-448.

143. Zhang X. *Study on the relationship between fractional low frequency amplitude of resting state functional magnetic resonance imaging and cognitive function in patients with first-episode schizophrenia* [硕士], Nanjing Medical University; 2019.

144. Zhao C, Zhu J, Liu X, et al. Structural and functional brain abnormalities in schizophrenia: A cross-sectional study at different stages of the disease. *Prog Neuropsychopharmacol Biol Psychiatry.* 2018;83:27-32.

145. Zhao XX, Yao JJ, Lv YD, et al. Abnormalities of regional homogeneity and its correlation with clinical symptoms in Naive patients with first-episode schizophrenia. *Brain imaging and behavior.* 2018;13(2):503-513.

146. Zhuo C, Zhu J, Qin W, Qu H, Ma X, Yu C. Cerebral blood flow alterations specific to auditory verbal hallucinations in schizophrenia. *The British journal of psychiatry : the journal of mental science.* 2017;210(3):209-215.

147. Zhou C, Tang XW, You W, et al. Altered Patterns of the Fractional Amplitude of Low-Frequency Fluctuation and Functional Connectivity Between Deficit and Non-Deficit Schizophrenia. *Frontiers in Psychiatry.* 2019;10.

148. Zhu J, Zhuo C, Qin W, et al. Altered resting-state cerebral blood flow and its connectivity in schizophrenia. *Journal of psychiatric research.* 2015;63:28-35.

149. Zhu J, Zhuo C, Liu F, Xu L, Yu C. Neural substrates underlying delusions in schizophrenia. *Scientific reports.* 2016;6:33857.

150. Zou W, Chen J, Huang S, Li R, Huang K, Chen J. Contrast study using resting-state fractional amplitude of low-frequency fluctuation between patients with first episode schizophrenia and first episode of bipolar disorder with psychotic symptoms. *Rdiol Practice.* 2019;34(04):399-404.

151. Boos HB, Cahn W, van Haren NE, et al. Focal and global brain measurements in siblings of patients with schizophrenia. *Schizophr Bull.* 2012;38(4):814-825.

152. Borgwardt SJ, Picchioni MM, Ettinger U, Toulopoulou T, Murray R, McGuire PK. Regional gray matter volume in monozygotic twins concordant and discordant for schizophrenia. *Biol Psychiatry.* 2010;67(10):956-964.

153. Guo W, Hu M, Fan X, et al. Decreased gray matter volume in the left middle temporal gyrus as a candidate biomarker for schizophrenia: a study of drug naive, first-episode schizophrenia patients and unaffected siblings. *Schizophrenia research.* 2014;159(1):43-50.

154. Honea RA, Meyer-Lindenberg A, Hobbs KB, et al. Is gray matter volume an intermediate phenotype for schizophrenia? A voxel-based morphometry study of patients with schizophrenia and their healthy siblings. *Biol Psychiatry.* 2008;63(5):465-474.

155. Hu M, Li J, Eyler L, et al. Decreased left middle temporal gyrus volume in antipsychotic drug-naive, first-episode schizophrenia patients and their healthy unaffected siblings. *Schizophrenia research.* 2013;144(1-3):37-42.

156. Huang P. Study of differences in the brain gray matter volume of first-episode schizophrenic patints and high-risk group and pattern recognition based on brain networks. *Fourth Military Medical University.* 2015.

157. Huang C, Liu C, Xue Z, Pu W. Abnormality of Brain Gray Matter in First-episode Schizophrenia Patients and Their Healthy Siblings. *Chinese Journal of Clinical Psychology.* 2016;24(2):207-212.

158. Job DE, Whalley HC, McConnell S, Glabus M, Johnstone EC, Lawrie SM. Voxel-based morphometry of grey matter densities in subjects at high risk of schizophrenia. *Schizophrenia research.* 2003;64(1):1-13.

159. Oertel-Knochel V, Knochel C, Matura S, et al. Cortical-basal ganglia imbalance in schizophrenia patients and unaffected first-degree relatives. *Schizophrenia research.* 2012;138(2-3):120-127.

160. Lei W, Deng W, Li M, et al. Gray matter volume alterations in first-episode drug-naive patients with deficit and nondeficit schizophrenia. *Psychiatry research.* 2015;234(2):219-226.

161. Li H, Fan M, Zhou C, Ning Y. Brain morphology of population at high risk of and patients with schizophrenia. *J Clin Psychiatry.* 2015;25(3):166-168.

162. Lin B, Li XB, Ruan S, et al. Convergent and divergent gray matter volume abnormalities in unaffected first-degree relatives and ultra-high risk individuals of schizophrenia. *Schizophrenia (Heidelberg, Germany).* 2022;8(1):55.

163. Lui S, Deng W, Huang X, et al. Neuroanatomical differences between familial and sporadic schizophrenia and their parents: an optimized voxel-based morphometry study. *Psychiatry research.* 2009;171(2):71-81.

164. McIntosh AM, Job DE, Moorhead TW, et al. Voxel-based morphometry of patients with schizophrenia or bipolar disorder and their unaffected relatives. *Biol Psychiatry.* 2004;56(8):544-552.

165. Tian L, Meng C, Yan H, et al. Convergent evidence from multimodal imaging reveals amygdala abnormalities in schizophrenic patients and their first-degree relatives. *PloS one.* 2011;6(12):e28794.

166. van der Velde J, Gromann PM, Swart M, et al. Grey matter, an endophenotype for schizophrenia? A voxel-based morphometry study in siblings of patients with schizophrenia. *Journal of psychiatry & neuroscience : JPN.* 2015;40(3):207-213.

167. Wagshal D, Knowlton BJ, Cohen JR, et al. Cognitive correlates of gray matter abnormalities in adolescent siblings of patients with childhood-onset schizophrenia. *Schizophrenia research.* 2015;161(2-3):345-350.

168. Amann BL, Canales-Rodríguez EJ, Madre M, et al. Brain structural changes in schizoaffective disorder compared to schizophrenia and bipolar disorder. *Acta psychiatrica Scandinavica.* 2016;133(1):23-33.

169. Ananth H, Popescu I, Critchley HD, Good CD, Frackowiak RS, Dolan RJ. Cortical and subcortical gray matter abnormalities in schizophrenia determined through structural magnetic resonance imaging with optimized volumetric voxel-based morphometry. *The American journal of psychiatry.* 2002;159(9):1497-1505.

170. Asami T, Bouix S, Whitford TJ, Shenton ME, Salisbury DF, McCarley RW. Longitudinal loss of gray matter volume in patients with first-episode schizophrenia: DARTEL automated analysis and ROI validation. *NeuroImage.* 2012;59(2):986-996.

171. Baršić AR, Rubeša G, Mance D, Miletić D, Gudelj L, Antulov R. Influence of psychotic episodes on grey matter volume changes in patients with schizophrenia. *Psychiatria Danubina.* 2020;32(3-4):359-366.

172. Barsic AR, Gudelj L, Antulov R, Miletic D, Knezevic K, Rubesa G. Onset of Schizophrenia prior to the end of brain maturation alters grey matter volume loss. *Psychiatria Danubina.* 2021;33:719-731.

173. Bassitt DP, Neto MR, de Castro CC, Busatto GF. Insight and regional brain volumes in schizophrenia. *European archives of psychiatry and clinical neuroscience.* 2007;257(1):58-62.

174. Berge D, Carmona S, Rovira M, Bulbena A, Salgado P, Vilarroya O. Gray matter volume deficits and correlation with insight and negative symptoms in first-psychotic-episode subjects. *Acta psychiatrica Scandinavica.* 2011;123(6):431-439.

175. Bonilha L, Molnar C, Horner MD, et al. Neurocognitive deficits and prefrontal cortical atrophy in patients with schizophrenia. *Schizophrenia research.* 2008;101(1-3):142-151.

176. Bose SK, Mackinnon T, Mehta MA, et al. The effect of ageing on grey and white matter reductions in schizophrenia. *Schizophrenia research.* 2009;112(1-3):7-13.

177. Brown GG, Lee JS, Strigo IA, Caligiuri MP, Meloy MJ, Lohr J. Voxel-based morphometry of patients with schizophrenia or bipolar I disorder: A matched control study. *Psychiatry Research-Neuroimaging.* 2011;194(2):149-156.

178. Cascella NG, Fieldstone SC, Rao VA, Pearlson GD, Sawa A, Schretlen DJ. Gray-matter abnormalities in deficit schizophrenia. *Schizophrenia research.* 2010;120(1-3):63-70.

179. Chang M, Womer FY, Bai C, et al. Voxel-Based Morphometry in Individuals at Genetic High Risk for Schizophrenia and Patients with Schizophrenia during Their First Episode of Psychosis. *PloS one.* 2016;11(10):e0163749.

180. Chen J, Wei Y, Xue K, et al. The interaction between first-episode drug-naive schizophrenia and age based on gray matter volume and its molecular analysis: a multimodal magnetic resonance imaging study. *Psychopharmacology.* 2023;240(4):813-826.

181. Cocchi L, Walterfang M, Testa R, et al. Grey and white matter abnormalities are associated with impaired spatial working memory ability in first-episode schizophrenia. *Schizophrenia research.* 2009;115(2-3):163-172.

182. Corradi-Dell'Acqua C, Tomelleri L, Bellani M, et al. Thalamic-insular dysconnectivity in schizophrenia: evidence from structural equation modeling. *Human brain mapping.* 2012;33(3):740-752.

183. Cui L, Deng W, Jiang L, et al. A Comparative Study of Voxel-based Morphometry in Patients with Paranoid Schizophrenia and Bipolar Mania. *J Sichuan Univ (Med Sci Edi).* 2010;41(01):5-9.

184. Cui L, Li M, Deng W, et al. Overlapping clusters of gray matter deficits in paranoid schizophrenia and psychotic bipolar mania with family history. *Neuroscience letters.* 2011;489(2):94-98.

185. Delvecchio G, Lorandi A, Perlini C, et al. Brain anatomy of symptom stratification in schizophrenia: a voxel-based morphometry study. *Nordic journal of psychiatry.* 2017;71(5):348-354.

186. DeRamus TP, Silva RF, Iraji A, et al. Covarying structural alterations in laterality of the temporal lobe in schizophrenia: A case for source-based laterality. *Nmr in Biomedicine.* 2020;33(6).

187. Egashira K, Matsuo K, Mihara T, et al. Different and shared brain volume abnormalities in late- and early-onset schizophrenia. *Neuropsychobiology.* 2014;70(3):142-151.

188. Euler M, Thoma RJ, Gangestad SW, Cañive JM, Yeo RA. The impact of developmental instability on Voxel-Based Morphometry analyses of neuroanatomical abnormalities in schizophrenia. *Schizophr Res.* 2009;115(1):1-7.

189. Fan YS, Xu Y, Li Q, et al. Systematically mapping gray matter abnormal patterns in drug-naive first-episode schizophrenia from childhood to adolescence. *Cerebral cortex (New York, NY : 1991).* 2023;33(4):1452-1461.

190. Fan Y, Gao Y, Ma Q, et al. Grey matter volume and its association with cognitive impairment and peripheral cytokines in excited individuals with schizophrenia. *Brain imaging and behavior.* 2022;16(6):2618-2626.

191. Filippi M, Canu E, Gasparotti R, et al. Patterns of brain structural changes in first-contact, antipsychotic drug-naïve patients with schizophrenia. *American Journal of Neuroradiology.* 2014;35(1):30-37.

192. Fukuta H, Ito I, Tateno A, et al. Effects of menopause on brain structural changes in schizophrenia. *Psychiatry Clin Neurosci.* 2013;67(1):3-11.

193. Giuliani NR, Calhoun VD, Pearlson GD, Francis A, Buchanan RW. Voxel-based morphometry versus region of interest: a comparison of two methods for analyzing gray matter differences in schizophrenia. *Schizophrenia research.* 2005;74(2-3):135-147.

194. Guo Q, Tang Y, Li H, et al. Both volumetry and functional connectivity of Heschl's gyrus are associated with auditory P300 in first episode schizophrenia. *Schizophrenia research.* 2014;160(1-3):57-66.

195. Guo XF, Li J, Wang J, et al. Hippocampal and orbital inferior frontal gray matter volume abnormalities and cognitive deficit in treatment-naive, first-episode patients with schizophrenia. *Schizophrenia research.* 2014;152(2-3):339-343.

196. Guo W, Liu F, Liu J, et al. Abnormal causal connectivity by structural deficits in first-episode, drug-naive schizophrenia at rest. *Schizophrenia bulletin.* 2015;41(1):57-65.

197. Guo F, Zhu YQ, Li C, et al. Gray matter volume changes following antipsychotic therapy in first-episode schizophrenia patients: A longitudinal voxel-based morphometric study. *J Psychiatr Res.* 2019;116:126-132.

198. Herold R, Feldmann A, Simon M, et al. Regional gray matter reduction and theory of mind deficit in the early phase of schizophrenia: a voxel-based morphometric study. *Acta psychiatrica Scandinavica.* 2009;119(3):199-208.

199. Hirao K, Miyata J, Fujiwara H, et al. Theory of mind and frontal lobe pathology in schizophrenia: a voxel-based morphometry study. *Schizophrenia research.* 2008;105(1-3):165-174.

200. Horn H, Federspiel A, Wirth M, et al. Gray matter volume differences specific to formal thought disorder in schizophrenia. *Psychiatry research.* 2010;182(2):183-186.

201. Huang C. Brain structure abnormality as genetic endophenotype of schizophrenia. *Chin J Med Genet.* 2009;26(5):490-494.

202. Huang P, Xi Y, Lu ZL, et al. Decreased bilateral thalamic gray matter volume in first-episode schizophrenia with prominent hallucinatory symptoms: A volumetric MRI study. *Scientific reports.* 2015;5:14505.

203. Hýža M, Huttlová J, Keřkovský M, Kašpárek T. Psychosis effect on hippocampal reduction in schizophrenia. *Progress in neuro-psychopharmacology & biological psychiatry.* 2014;48:186-192.

204. Jayakumar PN, Venkatasubramanian G, Gangadhar BN, Janakiramaiah N, Keshavan MS. Optimized voxel-based morphometry of gray matter volume in first-episode, antipsychotic-naïve schizophrenia. *Progress in Neuro-Psychopharmacology and Biological Psychiatry.* 2005;29(4):587-591.

205. Jin L. *Study on grey matter volume and resting state brain function of schizophrenia and first-degree relatives* [硕士], Xinjiang medical university; 2020.

206. Kang Y, Zhang Y, Huang K, Wang Z. The genetic influence of the DRD3 rs6280 polymorphism (Ser9Gly) on functional connectivity and gray matter volume of the hippocampus in patients with first-episode, drug-naïve schizophrenia. *Behavioural brain research.* 2022:114124.

207. Kaspárek T, Prikryl R, Mikl M, Schwarz D, Cesková E, Krupa P. Prefrontal but not temporal grey matter changes in males with first-episode schizophrenia. *Progress in neuro-psychopharmacology & biological psychiatry.* 2007;31(1):151-157.

208. Kawada R, Yoshizumi M, Hirao K, et al. Brain volume and dysexecutive behavior in schizophrenia. *Progress in neuro-psychopharmacology & biological psychiatry.* 2009;33(7):1255-1260.

209. Kim GW, Kim YH, Jeong GW. Whole brain volume changes and its correlation with clinical symptom severity in patients with schizophrenia: A DARTEL-based VBM study. *PloS one.* 2017;12(5):e0177251.

210. Koelkebeck K, Hirao K, Miyata J, et al. Impact of gray matter reductions on theory of mind abilities in patients with schizophrenia. *Social neuroscience.* 2013;8(6):631-639.

211. Kong L, Herold CJ, Zöllner F, et al. Comparison of grey matter volume and thickness for analysing cortical changes in chronic schizophrenia: a matter of surface area, grey/white matter intensity contrast, and curvature. *Psychiatry research.* 2015;231(2):176-183.

212. Kühn S, Romanowski A, Schubert F, Gallinat J. Reduction of cerebellar grey matter in Crus I and II in schizophrenia. *Brain structure & function.* 2012;217(2):523-529.

213. Lee DK, Lee H, Park K, Joh E, Kim CE, Ryu S. Common gray and white matter abnormalities in schizophrenia and bipolar disorder. *PLoS One.* 2020;15(5):e0232826.

214. Li C, Liu W, Guo F, et al. Voxel-based morphometry results in first-episode schizophrenia: a comparison of publicly available software packages. *Brain imaging and behavior.* 2020;14(6):2224-2231.

215. Li Q, Ran Y, Wei W, et al. An MRI study of brain structure of deficit and non-deficit types of first-episode drug-naïve patients with schizophrenia. *Chin J Nerv Ment Dis.* 2022;48(3):139-143.

216. Li M, Deng W, Li Y, et al. Ameliorative patterns of grey matter in patients with first-episode and treatment-naive schizophrenia. *Psychological medicine.* 2023;53(8):3500-3510.

217. Liao J, Yan H, Liu Q, et al. Reduced paralimbic system gray matter volume in schizophrenia: Correlations with clinical variables, symptomatology and cognitive function. *Journal of psychiatric research.* 2015;65:80-86.

218. Liu FJ, Shao Y, Li X, et al. Volumetric Abnormalities in Violent Schizophrenia Patients on the General Psychiatric Ward. *Frontiers in Psychiatry.* 2020;11.

219. Liu K. *Correlation between Changes in Brain Gray Matter Volume and Clinical Symptoms in Patients with Schizophrenia* [A Thesis Submitted for the Degree of Master], Xinxiang Medical University; 2022.

220. Lui S, Deng W, Huang X, et al. Association of cerebral deficits with clinical symptoms in antipsychotic-naive first-episode schizophrenia: an optimized voxel-based morphometry and resting state functional connectivity study. *The American journal of psychiatry.* 2009;166(2):196-205.

221. Lv S, Ouyang L, Huang X, et al. An optimized voxel-based morphometry MRI study of the brain inpatients with first episode schizophrenia. *CHIN J Radiol.* 2007;41(05):495-498.

222. Lyu H, Hu M, Eyler LT, et al. Regional white matter abnormalities in drug-naive, first-episode schizophrenia patients and their healthy unaffected siblings. *The Australian and New Zealand journal of psychiatry.* 2015;49(3):246-254.

223. Ma M, Zhang Y, Zhang X, Yan H, Zhang D, Yue W. Common and Distinct Alterations of Cognitive Function and Brain Structure in Schizophrenia and Major Depressive Disorder: A Pilot Study. *Front Psychiatry.* 2021;12:705998.

224. Madeira N, Duarte JV, Martins R, Costa GN, Macedo A, Castelo-Branco M. Morphometry and gyrification in bipolar disorder and schizophrenia: A comparative MRI study. *NeuroImage Clinical.* 2020;26:102220.

225. Maggioni E, Crespo-Facorro B, Nenadic I, et al. Common and distinct structural features of schizophrenia and bipolar disorder: The European Network on Psychosis, Affective disorders and Cognitive Trajectory (ENPACT) study. *PloS one.* 2017;12(11):e0188000.

226. Martí-Bonmatí L, Lull JJ, García-Martí G, et al. Chronic auditory hallucinations in schizophrenic patients: MR analysis of the coincidence between functional and morphologic abnormalities. *Radiology.* 2007;244(2):549-556.

227. Kenneth Martin A, Robinson G, Reutens D, Mowry B. Cognitive and structural neuroimaging characteristics of schizophrenia patients with large, rare copy number deletions. *Psychiatry research.* 2014;224(3):311-318.

228. McDonald C, Bullmore ET, Sham P, et al. Regional volume deviations of brain structure in schizophrenia and psychotic bipolar disorder - Computational morphometry study. *British Journal of Psychiatry.* 2005;186:369-377.

229. Molina V, Sanz J, Villa R, et al. Voxel-based morphometry comparison between first episodes of psychosis with and without evolution to schizophrenia. *Psychiatry research.* 2010;181(3):204-210.

230. Molina V, Hernández JA, Sanz J, et al. Subcortical and cortical gray matter differences between Kraepelinian and non-Kraepelinian schizophrenia patients identified using voxel-based morphometry. *Psychiatry research.* 2010;184(1):16-22.

231. Molina V, Galindo G, Cortés B, et al. Different gray matter patterns in chronic schizophrenia and chronic bipolar disorder patients identified using voxel-based morphometry. *European archives of psychiatry and clinical neuroscience.* 2011;261(5):313-322.

232. Molina V, Martín C, Ballesteros A, de Herrera AG, Hernández-Tamames JA. Optimized voxel brain morphometry: association between brain volumes and the response to atypical antipsychotics. *European archives of psychiatry and clinical neuroscience.* 2011;261(6):407-416.

233. Morgan KD, Dazzan P, Orr KG, et al. Grey matter abnormalities in first-episode schizophrenia and affective psychosis. *The British journal of psychiatry Supplement.* 2007;51:s111-116.

234. Moriya J, Kakeda S, Abe O, et al. Gray and white matter volumetric and diffusion tensor imaging (DTI) analyses in the early stage of first-episode schizophrenia. *Schizophrenia research.* 2010;116(2-3):196-203.

235. Nagashima T, Inoue M, Kitamura S, et al. Brain structural changes and neuropsychological impairments in male polydipsic schizophrenia. *BMC psychiatry.* 2012;12:210.

236. Nakamura K, Takahashi T, Nemoto K, et al. Gray matter changes in subjects at high risk for developing psychosis and first-episode schizophrenia: A voxel-based structural MRI study. *Frontiers in Psychiatry.* 2013;4(MAR).

237. Narayanaswamy JC, Kalmady SV, Venkatasubramanian G, Gangadhar BN. Clinical correlates of superior temporal gyrus volume abnormalities in antipsychotic-naïve schizophrenia. *The Journal of neuropsychiatry and clinical neurosciences.* 2015;27(2):e128-133.

238. Neckelmann G, Specht K, Lund A, et al. Mr morphometry analysis of grey matter volume reduction in schizophrenia: association with hallucinations. *Int J Neurosci.* 2006;116(1):9-23.

239. Nenadić I, Sauer H, Smesny S, Gaser C. Aging effects on regional brain structural changes in schizophrenia. *Schizophrenia bulletin.* 2012;38(4):838-844.

240. Nenadic I, Dietzek M, Schönfeld N, et al. Brain structure in people at ultra-high risk of psychosis, patients with first-episode schizophrenia, and healthy controls: a VBM study. *Schizophrenia research.* 2015;161(2-3):169-176.

241. Nenadic I, Maitra R, Langbein K, et al. Brain structure in schizophrenia vs. psychotic bipolar I disorder: A VBM study. *Schizophrenia research.* 2015;165(2-3):212-219.

242. Neugebauer K, Hammans C, Wensing T, et al. Nerve Growth Factor Serum Levels Are Associated With Regional Gray Matter Volume Differences in Schizophrenia Patients. *Frontiers in Psychiatry.* 2019;10.

243. O'Daly OG, Frangou S, Chitnis X, Shergill SS. Brain structural changes in schizophrenia patients with persistent hallucinations. *Psychiatry research.* 2007;156(1):15-21.

244. Onay A, Yapıcı Eser H, Ulaşoğlu Yıldız Ç, Aslan S, Talı ET. A combined VBM and DTI study of schizophrenia: bilateral decreased insula volume and cerebral white matter disintegrity corresponding to subinsular white matter projections unlinked to clinical symptomatology. *Diagnostic and interventional radiology (Ankara, Turkey).* 2017;23(5):390-397.

245. Ota M, Obu S, Sato N, Asada T. Neuroimaging study in subjects at high risk of psychosis revealed by the Rorschach test and first-episode schizophrenia. *Acta neuropsychiatrica.* 2011;23(3):125-131.

246. Paillère-Martinot M-L, Caclin A, Artiges E, Poline JB, Martinot JLJSR. Cerebral gray and white matter reductions and clinical correlates in patients with early onset schizophrenia. 2001;50(1-2):19-26.

247. Picado M, Carmona S, Hoekzema E, et al. The neuroanatomical basis of panic disorder and social phobia in schizophrenia: a voxel based morphometric study. *PloS one.* 2015;10(3):e0119847.

248. Plaze M, Paillere-Martinot ML, Penttila J, et al. "Where Do Auditory Hallucinations Come From?"-A Brain Morphometry Study of Schizophrenia Patients With Inner or Outer Space Hallucinations. *Schizophrenia bulletin.* 2011;37(1):212-221.

249. Salgado-Pineda P, Junqué C, Vendrell P, et al. Decreased cerebral activation during CPT performance: structural and functional deficits in schizophrenic patients. *NeuroImage.* 2004;21(3):840-847.

250. Salgado-Pineda P, Landin-Romero R, Fakra E, Delaveau P, Amann BL, Blin O. Structural abnormalities in schizophrenia: further evidence on the key role of the anterior cingulate cortex. *Neuropsychobiology.* 2014;69(1):52-58.

251. Sapara A, Ffytche DH, Cooke MA, Williams SCR, Kumari V. Voxel-based magnetic resonance imaging investigation of poor and preserved clinical insight in people with schizophrenia. *World Journal of Psychiatry.* 2016;6(3):311-321.

252. Schiffer B, Müller BW, Scherbaum N, et al. Impulsivity-related brain volume deficits in schizophrenia-addiction comorbidity. *Brain : a journal of neurology.* 2010;133(10):3093-3103.

253. Segarra N, Bernardo M, Valdes M, et al. Cerebellar deficits in schizophrenia are associated with executive dysfunction. *Neuroreport.* 2008;19(15):1513-1517.

254. Shen D, Li Q, Liu J, et al. The Deficits of Individual Morphological Covariance Network Architecture in Schizophrenia Patients With and Without Violence. *Front Psychiatry.* 2021;12:777447.

255. Shivakumar V, Kalmady SV, Rajasekaran A, et al. Telomere length and its association with hippocampal gray matter volume in antipsychotic-naïve/free schizophrenia patients. *Psychiatry research Neuroimaging.* 2018;282:11-17.

256. Singh S, Goyal S, Modi S, et al. Motor function deficits in schizophrenia: an fMRI and VBM study. *Neuroradiology.* 2014;56(5):413-422.

257. Singh S, Modi S, Goyal S, et al. Functional and structural abnormalities associated with empathy in patients with schizophrenia: An fMRI and VBM study. *Journal of biosciences.* 2015;40(2):355-364.

258. Singh S, Khushu S, Kumar P, Goyal S, Bhatia T, Deshpande SN. Evidence for regional hippocampal damage in patients with schizophrenia. *Neuroradiology.* 2018;60(2):199-205.

259. Spalthoff R, Gaser C, Nenadić I. Altered gyrification in schizophrenia and its relation to other morphometric markers. *Schizophrenia research.* 2018;202:195-202.

260. Suazo V, Díez Á, Montes C, Molina V. Structural correlates of cognitive deficit and elevated gamma noise power in schizophrenia. *Psychiatry and clinical neurosciences.* 2014;68(3):206-215.

261. Sun T, Zhao PF, Jiang XW, et al. Distinct Associations of Cognitive Impairments and Reduced Gray Matter Volumes in Remitted Patients with Schizophrenia and Bipolar Disorder. *Neural plasticity.* 2020;2020.

262. Szendi I, Szabo N, Domjan N, et al. A New Division of Schizophrenia Revealed Expanded Bilateral Brain Structural Abnormalities of the Association Cortices. *Frontiers in Psychiatry.* 2017;8.

263. Tomelleri L, Jogia J, Perlini C, et al. Brain structural changes associated with chronicity and antipsychotic treatment in schizophrenia. *European neuropsychopharmacology : the journal of the European College of Neuropsychopharmacology.* 2009;19(12):835-840.

264. Torres US, Duran FL, Schaufelberger MS, et al. Patterns of regional gray matter loss at different stages of schizophrenia: A multisite, cross-sectional VBM study in first-episode and chronic illness. *NeuroImage Clinical.* 2016;12:1-15.

265. Tregellas JR, Shatti S, Tanabe JL, et al. Gray matter volume differences and the effects of smoking on gray matter in schizophrenia. *Schizophrenia research.* 2007;97(1-3):242-249.

266. Tseng HH, Chiu CD, Chen KC, Lee IH, Chen PS, Yang YK. Absence of negative associations of insular and medial frontal gray matter volume with dissociative symptoms in schizophrenia. *Journal of psychiatric research.* 2021;138:485-491.

267. van Tol MJ, van der Meer L, Bruggeman R, Modinos G, Knegtering H, Aleman A. Voxel-based gray and white matter morphometry correlates of hallucinations in schizophrenia: The superior temporal gyrus does not stand alone. *NeuroImage Clinical.* 2014;4:249-257.

268. Venkatasubramanian G. Neuroanatomical correlates of psychopathology in antipsychotic-naive schizophrenia. *Indian Journal of Psychiatry.* 2010;52(1):28-36.

269. Wei GX, Ge L, Chen LZ, Cao B, Zhang X. Structural abnormalities of cingulate cortex in patients with first-episode drug-naïve schizophrenia comorbid with depressive symptoms. *Human brain mapping.* 2021;42(6):1617-1625.

270. Whitford TJ, Farrow TF, Gomes L, Brennan J, Harris AW, Williams LM. Grey matter deficits and symptom profile in first episode schizophrenia. *Psychiatry research.* 2005;139(3):229-238.

271. Witthaus H, Kaufmann C, Bohner G, et al. Gray matter abnormalities in subjects at ultra-high risk for schizophrenia and first-episode schizophrenic patients compared to healthy controls. *Psychiatry research.* 2009;173(3):163-169.

272. Wu Y, Jiao J, Zhang X, Han W. Observation of structural changes of cerebral gray matter in schizophrenia by magnetic resonance imaging. *Guizhou Medical Journal.* 2021;45(09):1474-1475.

273. Yang C, Wu S, Lu W, Bai Y, Gao H. Brain differences in first-episode schizophrenia treated with quetiapine: a deformation-based morphometric study. *Psychopharmacology.* 2015;232(2):369-377.

274. Yang ZY, Wang SK, Li Y, et al. Neural correlates of prospection impairments in schizophrenia: Evidence from voxel-based morphometry analysis. *Psychiatry research Neuroimaging.* 2019;293:110987.

275. Yang Y, Li X, Cui Y, et al. Reduced Gray Matter Volume in Orbitofrontal Cortex Across Schizophrenia, Major Depressive Disorder, and Bipolar Disorder: A Comparative Imaging Study. *Front Neurosci.* 2022;16:919272.

276. Yokoyama N, Sasaki H, Mori Y, et al. Additive Effect of Cigarette Smoking on Gray Matter Abnormalities in Schizophrenia. *Schizophrenia bulletin.* 2018;44(3):535-541.

277. Yue Y, Kong L, Wang J, et al. Regional Abnormality of Grey Matter in Schizophrenia: Effect from the Illness or Treatment? *PloS one.* 2016;11(1):e0147204.

278. Zhang C, Wang Q, Ni P, et al. Differential Cortical Gray Matter Deficits in Adolescent- and Adult-Onset First-Episode Treatment-Naïve Patients with Schizophrenia. *Scientific reports.* 2017;7(1):10267.

279. Zhang M, Xiang H, Yang F, et al. Structural brain imaging abnormalities correlate with positive symptom in schizophrenia. *Neuroscience letters.* 2022;782:136683.

280. Zhao X, Yao J, Lv Y, et al. Facial emotion perception abilities are related to grey matter volume in the culmen of cerebellum anterior lobe in drug-naïve patients with first-episode schizophrenia. *Brain imaging and behavior.* 2022.

281. Zierhut KC, Schulte-Kemna A, Kaufmann J, Steiner J, Bogerts B, Schiltz K. Distinct structural alterations independently contributing to working memory deficits and symptomatology in paranoid schizophrenia. *Cortex; a journal devoted to the study of the nervous system and behavior.* 2013;49(4):1063-1072.

282. Zou W, Li R, Huang J, Huang K, Chen J, Huang S. Voxel-based morphological analysis of brain gray matter Volume changes and resting-state fractional amplitude of low-frequency analysis in young patients with first episode schizophrenia. *Chin J Med Imaging Technol.* 2018;34(12):1782-1786.
